# Beta-Strand Interfaces of Non-Dimeric Protein Oligomers Are Characterized by Scattered Charged Residue Patterns

**DOI:** 10.1371/journal.pone.0032558

**Published:** 2012-04-09

**Authors:** Giovanni Feverati, Mounia Achoch, Jihad Zrimi, Laurent Vuillon, Claire Lesieur

**Affiliations:** 1 Université de Savoie, Annecy le Vieux Cedex, France; 2 Laboratoire de Chimie Bioorganique et Macromoléculaire (LCBM), Faculté des Sciences et Techniques-Guéliz, Université Cadi Ayyad, Marrakech, Maroc; 3 LAMA, Université de Savoie, Le Bourget du Lac, France; 4 AGIM, Université Joseph Fourier, Archamps, France; Ecole Polytechnique Federale de Lausanne, Switzerland

## Abstract

Protein oligomers are formed either permanently, transiently or even by default. The protein chains are associated through intermolecular interactions constituting the protein interface. The protein interfaces of 40 soluble protein oligomers of stœchiometries above two are investigated using a quantitative and qualitative methodology, which analyzes the x-ray structures of the protein oligomers and considers their interfaces as interaction networks. The protein oligomers of the dataset share the same geometry of interface, made by the association of two individual β-strands (β-interfaces), but are otherwise unrelated. The results show that the β-interfaces are made of two interdigitated interaction networks. One of them involves interactions between main chain atoms (backbone network) while the other involves interactions between side chain and backbone atoms or between only side chain atoms (side chain network). Each one has its own characteristics which can be associated to a distinct role. The secondary structure of the β-interfaces is implemented through the backbone networks which are enriched with the hydrophobic amino acids favored in intramolecular β-sheets (MCWIV). The intermolecular specificity is provided by the side chain networks via positioning different types of charged residues at the extremities (arginine) and in the middle (glutamic acid and histidine) of the interface. Such charge distribution helps discriminating between sequences of intermolecular β-strands, of intramolecular β-strands and of β-strands forming β-amyloid fibers. This might open new venues for drug designs and predictive tool developments. Moreover, the β-strands of the cholera toxin B subunit interface, when produced individually as synthetic peptides, are capable of inhibiting the assembly of the toxin into pentamers. Thus, their sequences contain the features necessary for a β-interface formation. Such β-strands could be considered as ‘assemblons’, independent associating units, by homology to the foldons (independent folding unit). Such property would be extremely valuable in term of assembly inhibitory drug development.

## Introduction

Most proteins are made of more than one polypeptide chain to carry out their biological function [1], [2]. They are referred to as protein oligomers and have what is called a quaternary structure. In addition, numerous monomeric proteins associate transiently in binary or in higher stœchiometries (number of chains associated in a protein oligomer) during their life span. The formation of protein oligomer, known as protein assembly, is also a common reaction used by pathogens to produce killing “machineries”. One good example is the pore forming toxins produced by pathogenic bacteria such as *Bacillus anthracis*, *Staphylococcus aurus* and *Aeromonas hydrohilae*. This mechanism is also responsible for protein misfolding diseases through the production of “amyloid” oligomers and fibers (e.g. Alzheimer, Parkinson, Creuzfeld Jacob) [3], [4], [5], [6], [7], [8], [9].

Intermolecular contacts (contacts between chains) exist only in multiple chain proteins. These contacts constitute what is called the protein interface and are formed through particular interaction patterns. Unfortunately, despite extensive analyses, the identification of the patterns responsible for permanent contacts remains difficult. This is due to the broad diversity of the contact solutions [10], [11]. The rationalization of known patterns of protein interfaces is also far from accomplished.

The patterns result from geometrical and chemical complementarities between the two partners. Numerous reports on protein interfaces, based on theoretical and experimental approaches, allow understanding some of the general rules underlying intermolecular contacts (for reviews see [2], [10], [12]).

First, one needs to distinguish within the interface, the amino acids involved in intermolecular contacts, the so called “hot spots”, from those who are not. Several programs can identify theoretical hot spot residues at interfaces based on: (i) distance cuts-off combined or not with some chemical selection, (ii) solvent accessible surfaces, (iii) geometrical selection (e.g. Voronoi cells) or (iv) evolutionary conserved residues [2], [13], [14], [15]. All require the atomic structure of the protein oligomer. Experimental evidences have also confirmed the presence of hot spot residues in interfaces (for review see [2]). One beautiful example is the selective effect of the mutation of only some of the residues of the interface on the protein assembly of the heptameric co-chaperone cpn10 [16].

Second, the interaction patterns of protein interfaces are related to their secondary and tertiary structures as it was initially described by Sir Francis Crick for α-coiled interfaces with the discovery of the heptaed sequences [17], [18], [19], [20], [21], [22], [23], [24]. The importance of the structure of the interface in the implementation of a particular motif has been now generalized with high-throughput interaction discovery [25], [26].

Third, at the amino acid level, a versatile solution has to be sought rather than a specific one. In fact, even for identical secondary structures, the geometry (triple helix, α-coiled, β-sandwich…) and/or the symmetry of the protein interfaces also affect the patterns at the amino acid levels [11], [17], [18], [20], [27], [28], [29], [30].

For a geometry of interface made of interacting β-strands (β-interfaces), dimers are the main stœchiometry studied, particularly when considering dataset analysis [21], [31], [32], [33], [34].

Here, we report the analysis of the β-interfaces of 40 soluble protein oligomers whose stoechiometries are from trimers to octamers. We used our tailor made program Gemini to select hot spots and to produce an interaction network -or a graph- of the subset of interactions that composes an interface [15]. Gemini quantitative and qualitative analyses reveal relatively long β-interfaces enriched with charged residues scattered within the interface. More precisely, arginine residues are preferred at N- and C- terminal extremities whereas histidine and glutamic acid residues are more frequent in the middle of the interfaces. Such a broad charge distribution has never been observed previously in dimeric β-interfaces or in intramolecular β-interactions.

## Materials and Methods

### Interfaces by Gemini

**Figure 1:**
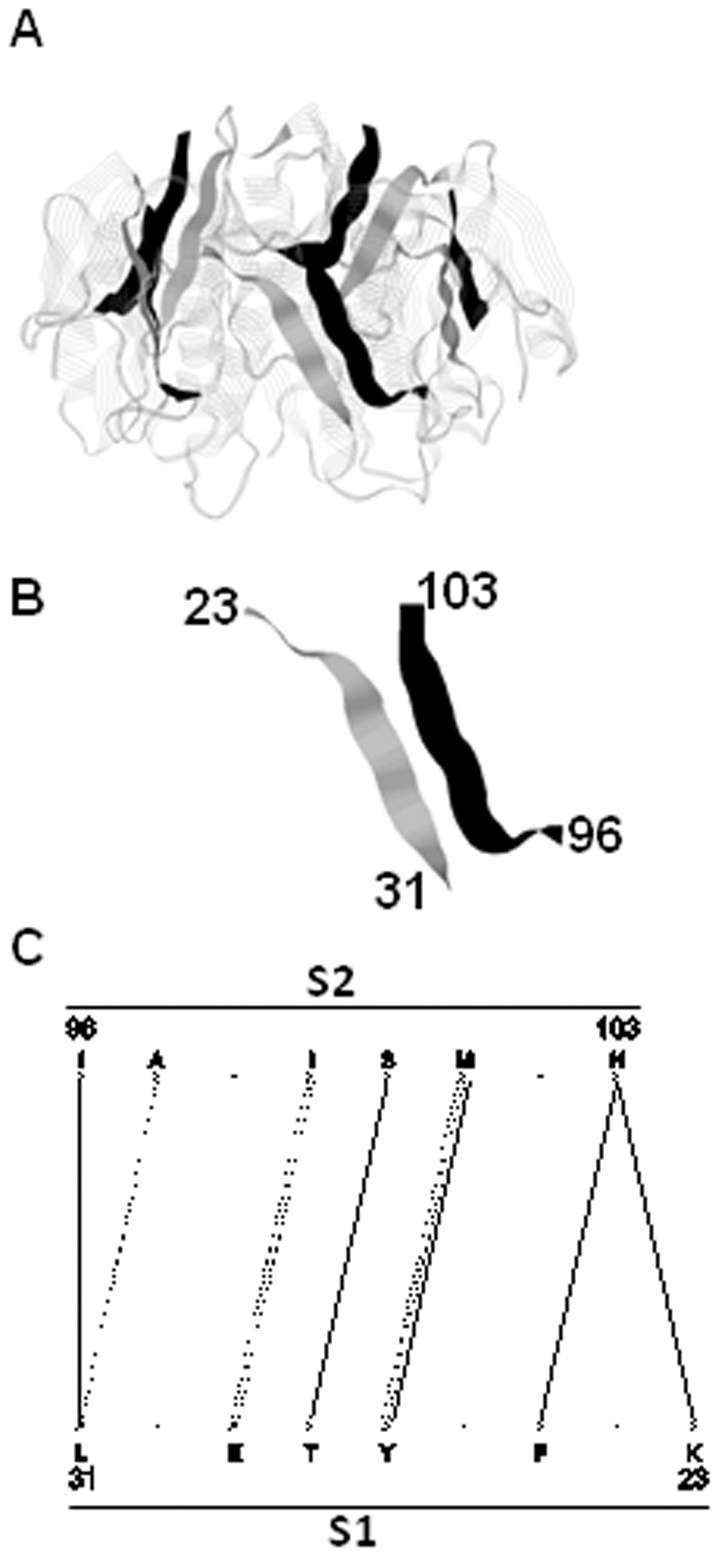
Example of one β-interface geometry. **A.** The x-ray structure of the whole cholera toxin B pentamer (CtxB_5_) is shown in strands (PDB code: 1EEI) [66]. The two strands of the β-interface are highlighted in black and grey in ribbons. The image has been generated using Rasmol. **B.** The β-interface is made of the association of the segment composed of amino acids 23 to 31 on one chain (segment 1) and of the segment composed of the amino acids 96 to 103 on the adjacent chain (segment 2). **C.** Gemini graph of the CtxB β-interface. S1 and S2 stand for segments 1 and 2.

The computer programs (Gemini) relevant to the present paper have been described previously [15]. In summary, Gemini characterizes an interface as a subset of amino acids in interaction, or “hot spots”. They emerge after a purely geometrical analysis of the 3D atomic structure of the protein, well described in the indicated publication. Gemini is equipped with an effective tool (GeminiGraph) that represents interfaces by (bipartite) graphs (Fig. 1). Throughout the paper, the graphs -and so the interfaces- are also referred to as ‘interaction networks’ or simply as ‘networks’. Briefly, the two segments S1 and S2, of an interface are represented by two parallel rows. The interacting amino acids selected by Gemini are indicated by ‘X’ and the non interacting ones by dots ‘.’ (Fig. 1C). The ‘X’ amino acids are the hot spots of the interface. The interactions (I) are illustrated by lines connecting two ‘X’. The version used here includes the name of the amino acids at positions ‘X’, following the one-letter code. In few cases, the β-interface is so intimately close to a different interface geometry that Gemini keeps them together in the same interface region (see Table S2 and Dataset S1). In the present work only the β-interface part has been used; the corresponding graphs have therefore been manually annotated (supplementary material).

A supplementary feature has been added to Gemini, which describes the interfaces as two interaction sub-networks. One of them only includes interactions between backbone atoms (BB sub-network), the other interactions with at least one side chain atom (SC sub-network). The interactions of the BB sub-network (I_BB_) are represented with dashed lines whereas those of the SC sub-network (I_SC_) are represented with solid lines. X_SC_ and X_BB_ are the side chain and backbone hot spots, respectively.

### Circular proteins

This is also a new addition to Gemini especially relevant to the present work. The goal of this part of the code is to recognize circular homo-oligomers (oligomers made of the same protein chain). The program classifies proteins into two classes: circular homo-oligomers and the rest that can contain hetero-oligomers and non circular homo-oligomers. For short, we call it non-circular (NC). The input information is the three-dimensional structure of PDB. No other database or author's annotation is used. The first step in the classification recognizes as NC those proteins whose chains are composed of different numbers of residues. Actually, given that in PDB files there can be additional or missing residues, an error of 25% is tolerated on the differences in the number of residues. The remaining proteins are therefore good candidates to be homo-oligomeric. In a second step, the program tries to find the first amino acid common to all the subunits. From it, five other common amino acids must be found, located at 15%, 30% and so on, of the sequence. If this step fails, the protein is NC. If it succeeds, the protein is very likely to be a homo-oligomer so a third step is needed to evaluate the spatial organization of the subunits. This is simply done by comparing the distances of the Cα of the six common amino acids already found. If the protein is a circular n-oligomer, there must be n identical distances (a tolerance of 5 Angstrom is used) otherwise the protein is NC. This algorithm is effective in finding circular homo-oligomers but is not enough to fully discriminate within the NC class. There are some false negatives, namely proteins that are circular homo-oligomers but are recognized as NC. This has the only effect of slightly reducing the size of our dataset. We did not observe false positives.

### Cytoscape (http://www.cytoscape.org/)

It is an open source bioinformatics software platform for visualizing molecular interaction networks and biological pathways and integrating these networks with annotations, gene expression profiles and other data. Although Cytoscape was originally designed for biological research, now it is a general platform for complex network analysis and visualization. Among the several types of interaction data supported, the format SIF (simple interaction format) was used for the present paper.

### RING (Residue Interaction Network Generator)

It is a web server with software for transforming a protein structure (in PDB format) into a network of interactions. Nodes represent single amino acids in the protein structure, while the edges represent the non-covalent bonding interactions that exist between them [35], [36], [37]. The interaction network and the edge attributes are stored in files with the SIF format. These files can then be easily loaded into CYTOSCAPE to visualize and manipulate the network [35], [36], [37]. In the present study, RING and CYTOSCAPE were used to produce and visualize the network of hydrogen bonds for the proteins of the dataset.

### Statistics

Median, quartile- The median is the value that splits the dataset into two equally populated subsets (above and below the median). For example, for 40 cases and a median of 180 amino acids in size, there are 50% of the cases with a length above 180 and 50% with a length below 180 amino acids. The quartile is the value at which the dataset it divided into four parts, equally populated with the 25% of the samples. The lower separation point is the first quartile, the middle one is the median and the higher is the third quartile.

### Global and Local propensity

The ratio between the amino acid frequency in a domain and the amino acid frequency in a database is called “*global propensity*”. If the global propensity is above 1, the amino acid is “preferred” in the domain and if the propensity is below 1, the amino acid is “disfavored” in the domain. The “*local propensity*” is defined by the ratio between the amino acid frequency in a particular position (e.g. corner) of a sub-domain (e.g. β-interface) and its frequency in all the other positions in the sub-domain. A local propensity above 1 means the amino acid is preferred in that position than anywhere else in the sub-domain [38]. On the contrary, a local propensity below 1 means the amino acid is disfavored in that position compared to elsewhere in the sub-domain. The corner positions are the amino acids located at the four outer positions on a segment: two outer positions on each side of the segment. So each segment has four amino acids positioned on corners and two outer interactions. The central positions are anywhere else on the segment.

### Secondary-structure prediction

GOR IV software was used to perform the secondary structure prediction of the segments of the proteins of the dataset. The secondary structure of each segment of the dataset was predicted (40×2 cases) considering all the wild-type amino acids of the segments and not only the -X-. Then, a residue was mutated and the secondary structure prediction was performed again. When a mutation affected the wild-type original secondary structure prediction, the mutated residue was considered important for the secondary structure of the segment. Hydrophobic residues of the BB or of the SC sub-networks, centrally located or at corners were mutated to charged residues (e.g. K, D, R, E, H). If one of the mutations affected the secondary structure prediction, mutation to other charged amino acids was not essayed. Polar and charged residues of the BB sub-networks centrally located in the full network, were also mutated to either polar or hydrophobic residues.

### Probability

Let's call p_c_ the probability to find in an interface, a charged amino acid. We now evaluate p_cc_, the probability to have at least one charged amino acid in (at least) one of the corners. This is evaluated as follows:
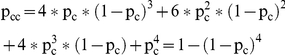
where each addendum is respectively the probability to find: a charged amino acid in one corner only, a charged amino acid in two corners, a charger amino acid in three corners, a charged amino acid in all corners. Everything holds true for the corner probability within one of the sub-networks, provided p_c_ is the corresponding probability.

### Reagents and buffers

Cholera toxin B pentamer (CtxB_5_) and all other chemicals were obtained from Sigma. McIlvaine buffer (0.2 M disodium hydrogen phosphate, 0.1 M citric acid, pH 7.0), PBS and 0.1 M KCl/HCl at pH 1.0 were used. All buffers were filtered through sterile 0.22 µm filter before use. Synthetic peptides were ordered from proteogenix (www.proteogenix.fr).

### SDS-PAGE analysis

SDS-PAGE (15% or 12%) were performed with a Bio-Rad mini-Protean 3 system using the Laemli method [39]. The gels were stained with Coomassie blue. 1 µg of sample was loaded on each lane of the gel.

### Reassembly of CtxB into native pentamer

The conditions used for reassembly were adapted from elsewhere [40]. Briefly, native CxtB_5_ was acidified in 0.1 M HCl/KCl at pH 1.0 for 15 min at a final toxin concentration of 86 µM, to induce the toxin dissociation into monomers (MW∼11 600 kDa). The toxin was subsequently diluted to a final concentration of 8,6 µM, in McIlVaine buffers at pH 7.0 to promote reassembly. The samples were incubated for 15 min at 23°C before analysis by SDS-PAGE. The reassembly into native CtxB pentamer was inferred from SDS-PAGE analyses since CtxB_5_ is stable in SDS-containing buffers and migrates in a gel, run on ice,with an apparent molecular weight characteristic of the B-subunit pentamer (MW∼55 000 kDa). Only the native pentamer is SDS-resistant. The CtxB concentration for all experiments refers to the monomeric concentration.

### Reassembly of CtxB in presence of peptides

The toxin reassembly was measured in presence of synthetic peptides whose sequences correspond to the toxin β-interfaces sequences (segments 1 and 2). The peptides were added in the neutralizing buffer at a molar ratio peptide to protein of 20. The reassembly conditions were identical to the one used for the toxin alone.

## Results

The primary goal of the analysis is to seek protein interface features within a dataset of protein oligomers sharing only a common geometry of interfaces. This is inspired by the success obtained for α-coiled interfaces [17], [18], [19]. The second objective is to see if the features can be rationalized in term of assembly mechanisms. The interfaces are analyzed using our tailor made program Gemini, which considers interfaces as interaction networks and allows both quantitative and qualitative studies [15].

### The dataset

The dataset was built by screening the Protein DataBank (PDB) [41]. First, cyclic protein oligomers were selected so all the cases had identical symmetry (circular, C_n_). To this purpose a program called “Circular” (materials and methods) was made. In total 502 protein oligomers were identified with stœchiometries from 3 (trimer) to 8 (octamer) (Table 1). Stœchiometries above 8 contained too few cases to be considered. Second, the secondary structure of the protein interface was chosen as two interacting β-strands at least 4 amino acids apart on the individual chain. The two interacting β-strands had to be different in their amino acid sequences (Fig. 1). Each strand is called a segment. Segment 1 (S1) appears first (N-terminal side) followed by segment 2 (S2) (C-terminal side) on the primary sequence. This geometry is referred to as a β-interface throughout the paper. Third, dimers, hetero-oligomers, transient oligomers, viral and membrane proteins were discarded from the dataset as their interfaces are likely to be differently programmed. After selection, the dataset was made of 40 protein interfaces but the list is non exhaustive.

**Table 1:** Circular protein oligomers containing a β-interface.

Category	Trimer	Tetramer	Pentamer	Hexamer	Heptamer	Octamer	Total
**Circular oligomers**	339	39	54	43	22	5	502
**β-interface**	13	6	11	4	4	2	40
**Circular oligomers (%)**	67 (339/502)	8	11	9	4	1	100

### Properties of the whole chain proteins of the dataset

**Figure 2:**
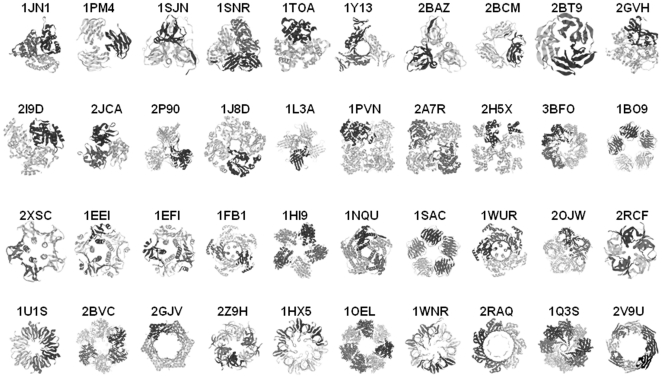
x-ray structures of the protein oligomers of the dataset. The respective PDB codes are indicated above the structures. The figure was made using RasMol. Each chain is shown in a different color.

The protein oligomers are produced by organisms from the three super-kingdoms of life with 2% of archea, 75% of bacteria and 23% of eukaryotes (Table S1). For comparison, there are 8%, 54% and 38% of archea, bacteria and eukaryotic protein oligomers for the stœchiometries from 3 to 8 in the PDB. The atomic structures (PDB) of the protein oligomers of the dataset are shown in figure 2 to illustrate the diversity of their quaternary, tertiary (folds) and secondary structures. The folds are also represented by the SCOP superfamily codes in Table S1 [42].

**Figure 3:**
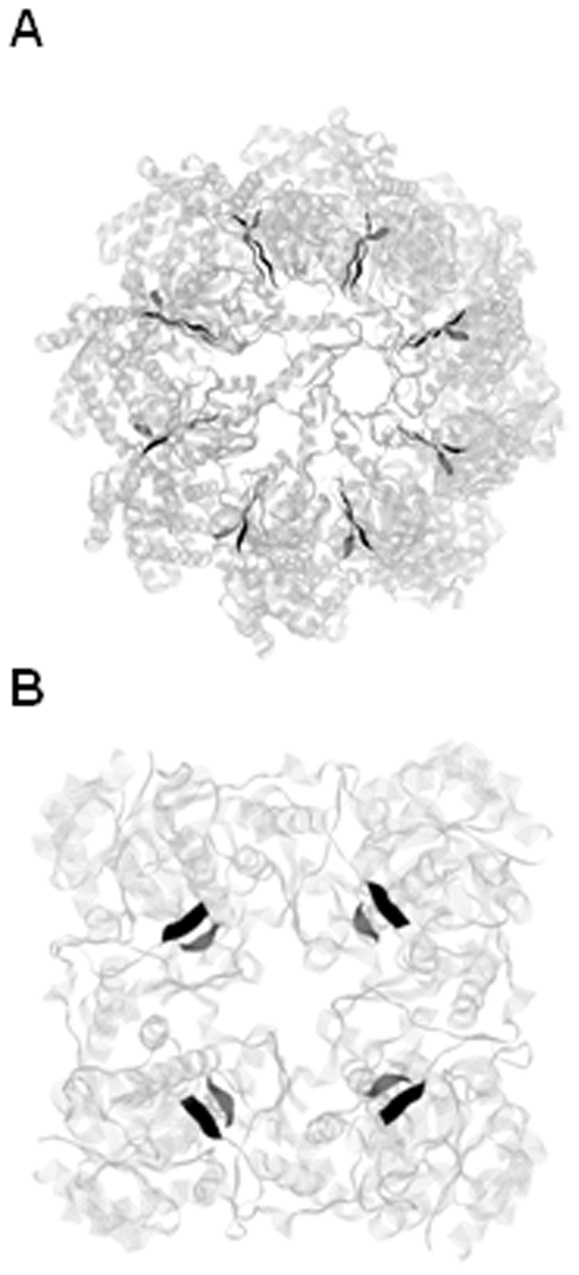
Protein oligomers containing a β-interface. **A.** The 1Q3S octameric bacterial chaperone [67] and **B.** The 1PVN tetrameric protozoa oxidoreductase [68]. Both structures are represented using RasMol. The chains are colored in light grey and the secondary-structures are represented by helices and strands. The β-strands of the interfaces are colored in black and dark grey in ribbons.

The secondary structure content of the whole chains is also extensively variable with on average on the dataset 30±20; 40±20 and 30±10% of α-, β- and random coiled structures. This is illustrated in figure 3 with the structures of the chaperone 1Q3S and of the oxidoreductase 1PVN which have a high content of α-structures (60 and 46%, respectively).

**Figure 4:**
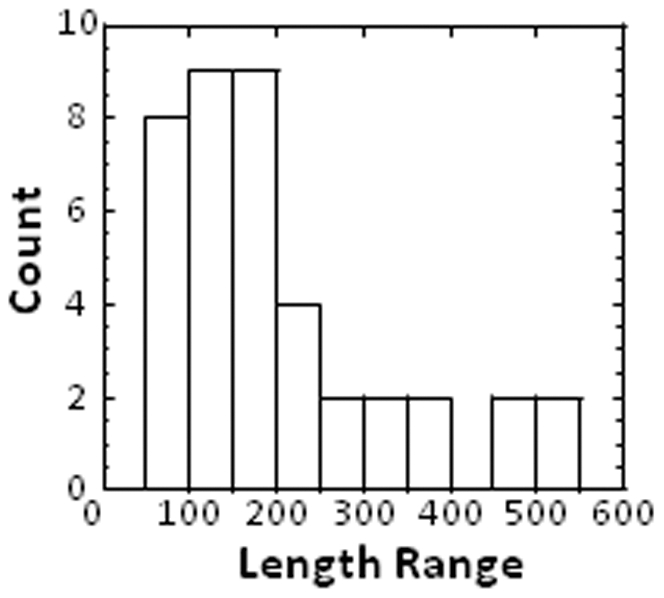
Histogram of the whole chain lengths. The length of the whole chain (range) is indicated on the x axis as the total number of amino acids.

The distribution of the whole chain lengths is broad as can be seen on the histogram on figure 4. The median length is 160 amino acids for an interquartile of 148 amino acids. The average length is 203±127 amino acids, value slightly smaller than the average length of monomeric proteins (∼300 amino acids) (Tables S1) [1]. This might be due to the measurement of the protein lengths from the PDB sequences which contain gaps due to crystallization or diffraction issues.

The circular trimers are the most represented (67%) against an average of 7±4% for the other stœchiometries (Table 1). The abundance of trimers might be related to the fact that the PDB over-represents low stœchiometries, dimer and trimer in particular, owing to the difficulties in crystallization. The β-interface geometry represents on average 8% of the circular protein oligomers (40/502) in good agreement with a previous measurement in dimers [21].

In summary, the protein oligomers of the dataset are produced by diverse organisms and cover a variety of functions, folds, amino acid lengths and stoichiometries (Table S1). Not surprisingly, the alignment of their amino acid sequences has no worthy of notice homology (not shown). Hence the dataset is characterized by a large heterogeneity.

### Global beta interface characteristics

Gemini's interaction networks (or graphs) of the β-interfaces are in Dataset S1. The length and the number of hot spots (-X-) of each β-interface, are determined using the Gemini graphs (materials and methods). Both are counted considering the two segments, S1 and S2, of the interface (Table S2). The statistics on hot spots, interface length and number of interactions are summarized in Table 2. The average length and number of hot spots for the segment S1 or for the segment S2, are similar, indicative of indistinguishable characteristics of the two β-strands of the β-interfaces. The number of interactions between two hot spots (X) involved in the β-interfaces (I_β_) is also provided by Gemini (Table S2 and Table 2).

**Table 2:** Statistics on the lengths of the dataset.

Sample	Average	SD^a^	Median	Q3-Q1	Q3^b^	Q1^b^
**Length**	17	6	17	7	19	12
**Hot spot ‘X’**	12	4	12	5	14	9
**Iβ**	10	4	10	5	12	7

a SD stands for standard deviation.

b Q stands for quartile. The statistics are defined in materials and methods.

The length, the hot spot number and the interaction number (I_β_) have medians and interquartile ranges fairly similar to their respective average and standard deviation values indicative of a relative homogeneity of these features throughout the dataset (Table 2). Yet there is no visible common topological feature within the graphs of the β-interfaces or any specific chemical composition compared to the whole chains (Table 3). A slightly different chemical composition appears when the hot spots are considered instead of all the amino acids of the two segments S1 and S2 (Table 3). No particular sequence homology was observed upon alignments of the S1 and S2 segments (not shown).

**Table 3:** Average chemical composition, in percentage, of the amino acids of the whole chain of the protein dataset, of the two segments of the interface S1+S2) and of the hot spots of S1 and S2. SC and BB stand for side chain and backbone amino acids, respectively.

Interfaces	whole	S1+S2	S1+S2 ‘X’	X_SC_	X_BB_
**Charged**	24±17	24±10	28±14	30±17	23±16
**Polar**	23±15	26±14	29±16	29±17	27±24
**Hydrophobic**	53±34	50±12	45±15	41±15	50±14

It was then assumed that common features might be somehow diluted in a ‘background’ noise.

As the backbone atoms are identical for the twenty amino acids, it was possible that counting them in the chemical properties of the β-interfaces ‘hid’ some chemical specificity only distinguishable on the side chain atoms. Likewise, only the backbone atoms might carry topological information. Moreover, previous studies on protein interfaces had indicated the importance of distinguishing main chain (backbone atoms) contacts from side chain contacts [2], [43], [44].

Accordingly, the graphs of the β-interfaces were partitioned in two sub-graphs, one made of the backbone interactions (one atom of the backbone per segment, BB sub-networks) and one made of the side chain interactions (one atom of the side chain per segment or one atom of the side-chain on a segment and one atom of the backbone on the other segment, SC sub-network). They are shown in supplementary material 1 (Dataset S1). The interactions within the BB sub-networks are illustrated with dashed lines whereas the interactions within the SC sub-networks are illustrated with solid lines (see also materials and methods). It is important to note that the BB and SC sub-graphs can be considered individually (not considering the whole graphs) or within the whole graph. This nuance is important and when the two sub-networks are considered together, we will refer to as the “full” graph or the full network.

### Characteristics of the BB sub-networks

The discrimination of the BB and SC sub-networks revealed significant features shared by the β-interfaces.

**Figure 5:**
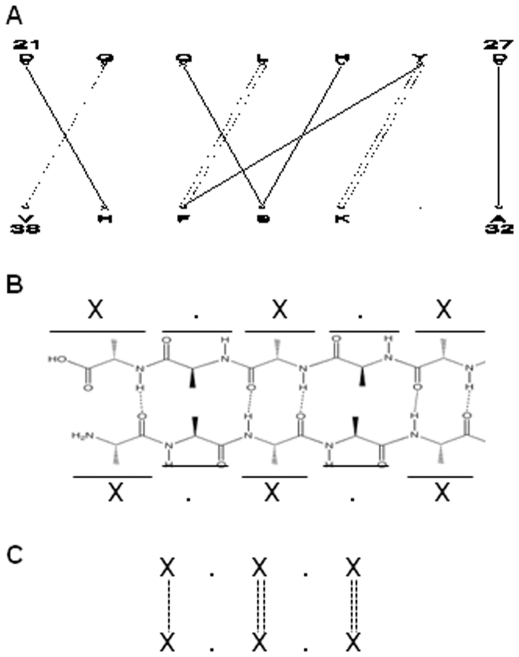
Anti-parallel BB sub-network and intramolecular hydrogen bond network. **A.** Gemini graph of an anti-parallel intermolecular β-interface **B.** Schematics of the hydrogen bond network of anti-parallel intramolecular β-sheet. **C.** Ladder pattern observed in BB sub-network and also visible in anti-parallel intramolecular β-sheet.

**Figure 6:**
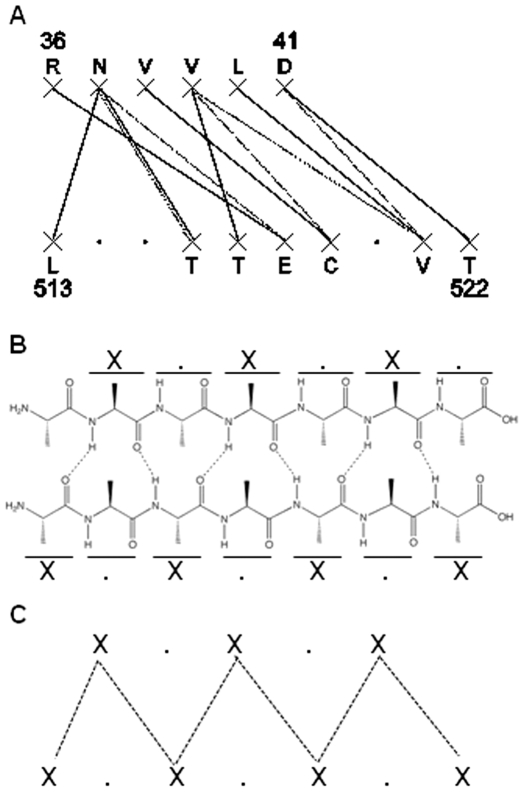
Parallel BB sub-network and intramolecular hydrogen bond network. **A.** Gemini graph of a parallel intermolecular β-interface **B.** Schematics of the hydrogen bond network of parallel intramolecular β-sheet. **C.** Ladder pattern observed in BB sub-network and also visible in parallel intramolecular β-sheet.

The BB sub-networks appeared characterized by common topological features but not by chemical specificities. First, different patterns of interactions show up in the BB sub-graphs. The first one, which appears in 19 graphs, is referred to as the “ladder” pattern because the BB interactions are running parallel to one another (Fig. 5). The second pattern which appears in 8 graphs is referred to as the “V-shape” pattern because it's a triplet interaction in the shape of a -V- (Fig. 6). The patterns are defined by elementary interaction blocks. One block “X.X” on one segment interacts with one block “X.X” on the other segment in the ladder pattern. One “X” on one segment interacts with one block “X.X” on the other segment in the V-shape pattern. The elementary blocks appear singly or in multiple copies. Single versions of the ladder pattern appear in 1PVN, 2OJW, 1U1S and 1HX5 and in multiple copies in 1PM4, 1SNR, 1HI9, 1WUR, 2BCM, 2RCF, 2GJV, 2GVH, 2P90, 1J8D, 1WNR, 2RAQ, 1EEI and 1EFI . There are slightly altered versions of the ladder pattern. One graph (1FB1) is made of one block “X.X” on one segment interacting with one block “X . . X” on the other segment. Two graphs (2I9D and 2RCF) have one block of “XX” on one segment interacting with one block “XX” on the other segment.

Single version of the V-shape pattern can be observed in 2A7R and 2V9U and in multiple copies in 1SJN, 2BAZ, 1L3A, 1NQU, 1OEL and, 1Q3S.

There are also 5 graphs made of a mix of ladder and V-shape patterns (1Y13, 2I9D, 2H5X, 3BFO, 2Z9H).

The second topological information of the BB sub-networks is the fact that the ladder and the V-shape patterns appear related to the arrangement of the secondary structures of the β-interfaces. Indeed, they are observed mostly in anti-parallel and in parallel intermolecular β-strand interactions, respectively, and the pattern shapes' are reminiscent of the anti-parallel and parallel intramolecular main chain hydrogen bond networks found in β-sheets (Figs. 5B & 5C and 6B & 6C). To determine whether Gemini's BB networks were related to intermolecular hydrogen bonds, the program RING (materials and methods) was used, showing that out of the 100 atoms detected by RING as participating in hydrogen bonds, 98 are Gemini's backbone atoms. This is likely due to the selection process of Gemini which retains the closest atoms [15]. Gemini detects slightly more backbone atoms and bonds than RING (139 against 100) due to the fact that Gemini is able to detect the double interactions per amino acids observed in the hydrogen bond network of intramolacular β-sheets (Fig. 5B & 5C and 6B & 6C). Thus, the BB sub-networks describe intermolecular β-sheets. This is confirmed by the observation that the graphs which have no BB interaction (1JN1, 1T0A, 2JCA, 1B09, 2XSC, 1SAC) or only one BB interaction (2BT9 and 2BVC) are not intermolecular β-sheets but are two rather perpendicular interacting β-strands, as can be seen on their respective PDB.

The BB sub-networks (X_BB_) cannot be distinguished from the whole chains by a specific chemical composition (charged, polar and hydrophobic amino acids). Yet, they are dominated by hydrophobic properties: half of the amino acids of the BB sub-networks are hydrophobic and a third of the interactions are purely hydrophobic (Table 3 and table 4).

**Table 4:** Chemical composition of the interactions (amino acid -i- of segment 1 with amino acid -j- of segment 2 or vice-versa, data are added together) in the SC and in the BB (bracket) networks of the β-interfaces.

Chemical properties	Charged	Polar	Hydrophobic
**Charged**	17% (3,5%)		
**Polar**	13% (13%)	10% (9%)	
**Hydrophobic**	18% (23%)	25% (21%)	16% (30%)

The global propensity (materials and methods) of the hydrophobic amino acids of the BB sub-networks was measured to evaluate which hydrophobic amino acids were over-represented in the β-interfaces compared to the whole chains (Table 5). A global propensity above 1 indicates a hydrophobic amino acid “preferred” in the BB sub-networks and on the contrary, a global propensity below 1, indicates a hydrophobic amino acid depleted in the BB sub-networks. Methionine (M), cysteine (C), tryptophane (W), isoleucine (I) and valine (V) are preferred in the BB sub-networks whereas proline (P), alanine (A), glycine (G) and leucine (L) residues are not favored in the BB sub-networks. The phenylalanine is equally present in the BB sub-networks and in the whole chains of the dataset (Global propensity around 1).

**Table 5:** Global propensity of the hydrophobic residue in the BB sub-networks.

Hydrophobic	Number in the BB sub-networks	Percentage in the BB sub-networks	Number in the Whole chains	Percentage in the whole chains	Global propensity
**I**	26	0.20	577	0.13	1.6
**L**	17	0.13	700	0.16	0.8
**V**	27	0.21	714	0.16	1.3
**A**	13	0.10	756	0.17	0.6
**C**	4	0.03	95	0.02	1.5
**M**	11	0.09	180	0.04	2.1
**F**	9	0.07	295	0.07	1.1
**G**	15	0.12	714	0.16	0.7
**P**	4	0.03	351	0.08	0.4
**W**	3	0.02	82	0.02	1.3
**Total**	129		4464		

### Characteristics of the SC sub-networks

In contrast to the BB sub-networks, the SC sub-networks have no topological information but some chemical specificity. In fact the SC sub-networks present an average chemical composition significantly different from the whole chains with a decrease of the percentage of hydrophobic amino acids in favor of an increase of the percentage of charged amino acids (Table 3). The percentage of polar residues remains similar for the SC sub-networks and the whole chains. This observation is even more obvious when the interactions (I_SC_) are considered instead of the individual amino acids (X_SC_), as the SC sub-networks have 5 times more purely charged interactions (Ch-Ch) than the BB sub-networks (Table 4). The SC sub-networks also have twice less purely hydrophobic interactions (F-F) than the BB sub-networks (Table 4).

The global propensity (materials and methods) of the charged residues of the SC sub-networks compared to the whole chains is reported in Table 6. A charged amino acid with a global propensity above 1 is “preferred” in the SC sub-networks whereas a charged amino acid with a propensity below 1 is depleted. Apart from the histidine, which has a global propensity slightly above 1.0, all the charged residues of the SC sub-networks have a global propensity around 1.

**Table 6:** Global propensity of the charged residue in the SC sub-networks.

Charged	Number in the SC sub-networks	Percentage in the SC sub-networks	Number in the whole chains	Percentage in the whole chains	Global propensity
**R**	19	0.16	403	0.18	0.9
**E**	31	0.27	584	0.27	1.0
**K**	26	0.22	497	0.23	1.0
**D**	24	0.21	507	0.24	0.9
**H**	13	0.11	188	0.09	1.3
**Total**	113		2179		

The local propensity of the charged amino acids in the SC sub-networks was analyzed considering corner (the four outer SC amino acids) and central (non corner) positions (Table 7 and table 8, respectively). The local propensity (material and methods) is the ratio of the frequency of an amino acid in a particular position (e.g. corner) within a local structure (e.g. the β-interfaces) and of the frequency of the same amino acid in any other position within that local structure [38]. There are almost as much charged amino acids at corners than at central positions (44% in corner positions). But the two positions are made of different types of charged residues. Arginine (R) residues are more frequent at corners (local propensity above 1 in table 7) whereas it is glutamic acid and histidine residues which are favored centrally (local propensity above 1 in table 8). The lysine and aspartic acid residues have no local preferences (local propensity around 1 in both table 7 and table 8).

**Table 7:** Local propensity of the corner charged residue in the SC sub-networks.

Charged	Number in the corner position	Percentage in the corner position	Number in the SC sub-networks	Percentage in the SC sub-networks	Local propensity
**R**	12	0.24	19	0.17	1.4
**E**	11	0.22	31	0.27	0.8
**K**	12	0.24	26	0.23	1.0
**D**	11	0.22	24	0.21	1.0
**H**	4	0.08	13	0.12	0.7
**Total**	51		113		

**Table 8:** Local propensity of the corner charged residue in the SC sub-networks.

Charged	Number in a NOT corner position	Percentage in a NOT corner position	Number in the SC sub-networks	Percentage in the SC sub-networks	Local propensity
**R**	7	0.11	19	0.17	0.7
**E**	20	0.32	31	0.27	1.2
**K**	14	0.22	26	0.23	1.0
**D**	13	0.21	24	0.21	1.0
**H**	9	0.14	13	0.12	1.2
**Total**	64		113		

### Comparison of BB and SC sub-networks

There exist several differences between the BB and the SC sub-networks (Table S3). There are 6±3 I_SC_ interactions for only 4±2 I_BB_ interactions. Additionally, there are 9±4 X_SC_ amino acids for only 5±3 X_BB_ amino acids. An amino acid with one atom involved in a BB interaction and one atom involved in a SC interaction is counted twice, one per network. But an amino acid having several atoms participating to the same network is counted only once. Thus, on average, the SC sub-network is bigger than the BB sub-network with roughly 60% of the interface amino acids and interactions devoted to it.

When considering the full graphs, it appears that the BB sub-networks are depleted of interactions and of hot spots at corners having only two graphs with two I_BB_ in the outer positions (1NQU and 2Z9H) and only 11 with one I_BB_ in the outer position (1Y13, 2BCM, 1PVN, 2A7R, 2H5X, 3BFO, 1EFI, 2OJW, 1U1S, 1WNR AND 1Q3S). In contrast, 28 graphs have two SC interactions in the outer positions and 39 (out of 40) have at least one. Likewise, the SC sub-networks are depleted of interactions and of hot spots at central positions. There are 86 I_SC_ centrally located for a total of 240 I_SC_ (36%) and 143 X_SC_ centrally located for a total of 374 X_SC_ (38%). In the BB sub-networks, there are 86 I_BB_ centrally located for a total of 156 I_BB_ (55%) and 131 X_BB_ centrally located for a total of 219 X_BB_ (60%). This means that in a typical arrangement, the SC sub-network spatially contains and surrounds the BB one.

Consequently, the corners of the SC sub-networks are enriched with charged residues (32 graphs out of 40, 80%) while those of the BB sub-networks are depleted (10 graphs out 34: 29%). Similarly, the BB sub-networks are enriched centrally with hydrophobic residues (72 central hydrophobic residues for 110 in total: 65%) while the SC sub-networks are depleted (41 central hydrophobic residues for 101 in total: 41%).

Hence, the relative position of the sub-networks provides enrichment (or depletion) of a chemical property without having to vary the absolute number of amino acids of that property in the sub-networks. For example, there are 110 and 101 hydrophobic residues in the BB and SC sub-networks, respectively. Also, the probabilities of finding a charged residue in the corner of the SC or of the BB sub-networks, based on their respective chemical properties (Table 3), are indeed very similar 76% and 65%, respectively (materials and methods). Yet by positioning the X_BB_ centrally, the charged X_SC_ appear more frequently at corners.

### Rationalization of the BB and SC features

Once common features are identified within the β-interfaces of the dataset, the next question is: can those features be rationalized in term of protein assembly or interface formation?

The first argument in that direction, is the weight of the β-interactions (Table S2). I_β_ are the interactions involved in the β-interface region of the protein oligomers of the dataset. Now, the total number of intermolecular interactions (I_tot_) in a whole chain is the number of interactions in all the interface regions. I_tot_ is provided by Gemini. The average number of intermolecular interactions (I_av_) per chain is the total number of interactions (I_tot_) divided by the number of interface regions. The weight of the β-interactions is measured by the ratio -I_β_/I_av_- which gives the amount of interactions in a β-interface compared to the average number of interactions in the whole chains. On average, there are twice more interactions in the β-interfaces than in the whole interface (1.8±0.6). The high number of interactions due the beta geometry is consistent with a role of the β-interfaces in the assembly mechanism.

The data indicate that the BB sub-networks are related to the secondary structures of the interfaces and that they are enriched in hydrophobic residues and hydrophobic interactions. In order to test the involvement of the hydrophobic residues in the secondary structure of the interface, the effect of their mutation on secondary structure prediction was investigated.

The secondary structure of the segments (S1 and S2) with the wild-type (WT) sequence was predicted using GOR IV and compared to the prediction of the same segment after a point mutation of one hydrophobic residue. The mutation of centrally located hydrophobic residues to a charged residue (e.g. K, D, R, E, H) altered the secondary-structure prediction in 83% of the cases. The mutation of hydrophobic residues located at corners to charged residue, also disturbed the secondary-structure prediction but to a much lesser extent (44% of the cases). In the same way, the mutation of polar or of charged residues of the BB sub-networks centrally located, to hydrophobic, charge or polar amino acids affected the secondary-structure prediction in only 44% of the cases.

We then measured the local propensity of the hydrophobic residues located centrally in the BB sub-networks and affecting the 2D structure prediction (Table 9). It appears that among the secondary-influencing hydrophobic residues centrally located, the valine (V) and the phenylalanine (F) are preferred (local propensity above 1). The leucine (L), the isoleucine and the methionine (M) appear neutral in the central position (local propensity around 1). Tryptophan (W), proline (P), glycine (G), alanine (A) and cysteine (C) are not favored (local propensity below 1).

**Table 9:** Local propensity of the central hydrophobic residue of the BB sub-networks affecting the 2D-structure prediction.

Hydrophobic	Number in central position	Percentage in the central position	Number in BB sub-networks	Percentage in BB sub-networks	Local propensity
**I**	11	0.20	26	0.20	1.0
**L**	8	0.15	17	0.13	1.1
**V**	18	0.33	27	0.21	1.6
**A**	3	0.06	13	0.10	0.6
**C**	1	0.02	4	0.03	0.6
**M**	4	0.07	11	0.09	0.9
**F**	5	0.09	9	0.07	1.3
**G**	3	0.06	15	0.12	0.5
**P**	1	0.02	4	0.03	0.6
**W**	0	0.00	3	0.02	0.0
**Total**	54		129		

The local propensity results were tested using secondary-structure prediction again. Mutations of central hydrophobic amino acids of the BB sub-networks to hydrophobic amino acids which have a local propensity above 1 were expected to have a secondary-structure prediction identical to the wild-type one. This is referred to as the amino acid having a positive versatility (act as wild-type amino acid). On the contrary, mutations to amino acid with a local propensity below 1 were expected to alter the wild-type secondary-structure prediction. These amino acids are referred to as having a negative versatility. In total 331 mutations-predictions have been performed and on average 69% behave as expected (229/331). Both the versatilities are giving similar results with 67% (116/172) of the mutations to amino acids of positive versatility not affecting the secondary structure prediction and 71% (113/159) of the mutations to amino acids of negative versatility affecting it.

This is consistent with the involvement of the features of the BB sub-networks in the secondary structure formation of the β-interfaces.

The SC sub-networks have no topological information and therefore cannot be related to geometrical features. But they have enrichment in charged residues and more precisely a specific distribution of the type of charges along the interface. This suggests a chemical role of the SC sub-networks in the formation of the β-interfaces, via electrostatic interactions.

We have seen that the local positions of the hydrophobic and of the charged residues of the BB and SC sub-networks were connected to the relative position of the two sub-networks. Now, remarkably for the 11 graphs which have one outer BB interaction, 7 have one charged BB residue at a corner. Following the same drift, the graphs with a low content of SC interactions but made of a majority of BB interactions have a charged BB residue in a corner in 44% of the case (7 out 16 graphs) whereas this occurs only in 12% of the graphs made of a minority of BB interactions (3/24).

So even if having a charged residue in a corner appears a trademark of the SC sub-networks, a corner charged residue is maintained via the BB sub-networks if necessary. This looks like a compensatory or a substitutive mechanism.

**Figure 7:**
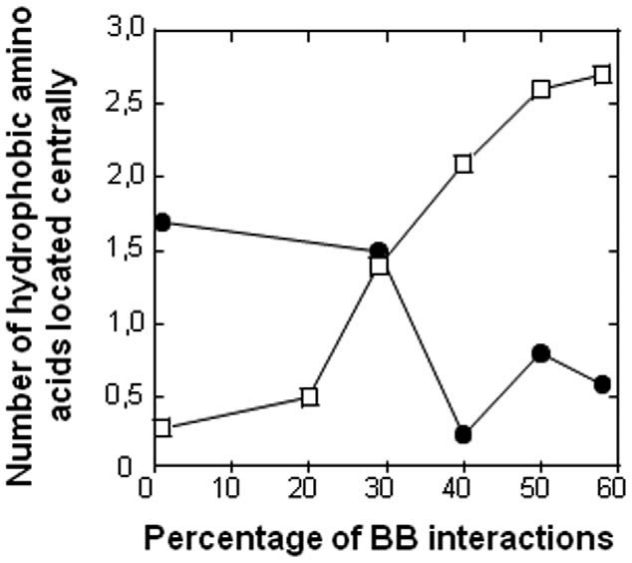
Central hydrophobic residues and percentage of BB interactions. The number of hydrophobic amino acids of the BB (β) or of the SC sub-networks (•) located centrally in the full networks are plotted against the percentage of BB interactions.

A similar phenomenon can be observed for the hydrophobic property of the graphs. On average twice more SC hydrophobic residues are located centrally (1,1 central SC hydrophobic) in graphs made of a minority of BB interactions than in graphs made of a majority of BB interactions (0,45 central SC hydrophobic). More precisely, the number of centrally located hydrophobic residues is maintained at a value of 2,8±0,6 across the dataset with 2,2±0,5 of them affecting the secondary structure predictions (Fig. 7). This value is kept constant using either BB or SC residues, or a balance of both. The mutation of the centrally located hydrophobic residues of the SC sub-networks to charged residue affects the secondary prediction in 83% of the case, as for the BB sub-networks. Thus the regulation of the secondary structure through hydrophobic amino acids located centrally is organized by the BB sub-networks in most cases. But the BB sub-networks can be substituted by the SC sub-networks as an alternative.

Such compensatory or substitutive phenomenon is also in favor of the features being involved in the formation of the interface.

No distinction between the stœchiometries was found for any of the properties of the β-interfaces (not shown).

### Autonomous β-interface segments

**Figure 8:**
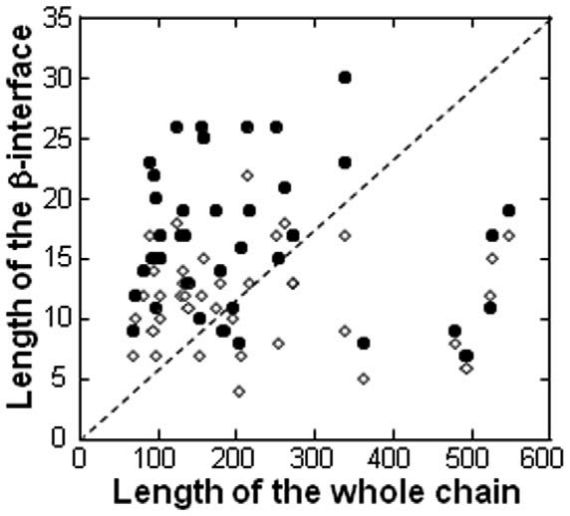
Absence of correlation between the lengths of the whole chains and of the β-interfaces. The length of the β-interface (sum of the amino acids of the two segments) of each protein of the dataset is plotted against the length of its respective whole chain (•, ‘all amino acids’ and ◊, ‘X’, respectively). If there was a correlation between the size of the whole chain and the size of its interface or the size of its hot spot numbers, the points would appear on the dashed line.

**Figure 9:**
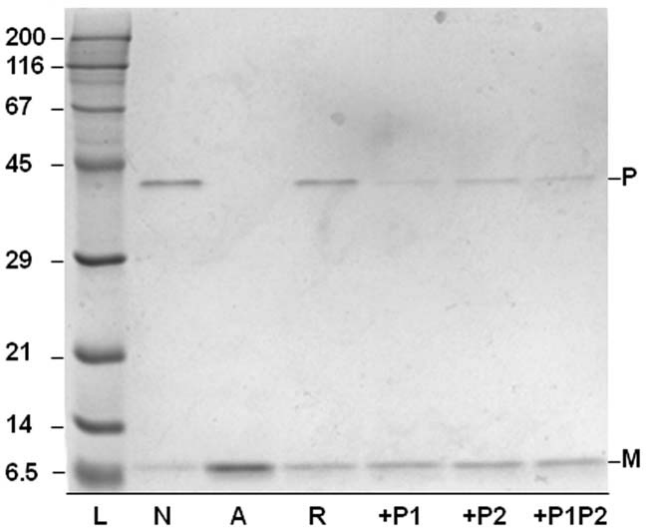
*In vitro* assembly of the cholera toxin B subunit into pentamer (CtxB_5_). The formation of the CtxB β-interface is monitored by SDS-PAGE. The initial native CtxB_5_ is indicated in lane 2 (N) whereas the acidified CtxB monomer is indicated in lane 3 (A). The toxin reassembly after 15 min in neutral condition is shown from lane 4 to 7 for the toxin alone (R, lane 4), or with a synthetic peptide of CtxB segment 1 sequence (+P1, lane 5) or with a synthetic peptide whose sequence corresponds to CtxB segment 2 (+P2, lane 6) or with a mixture of both peptides (+P1P2, lane 7). L stands for low molecular weight standard.

As mentioned earlier, the features describing the β-interfaces are rather homogeneous compared to the heterogeneity observed for their whole chains. In addition, it seems possible to associate the β-interface features to geometrical and chemical properties. This hinted the possibility that the β-interfaces had some autonomous capacity to associate in absence of the whole chain. This was further supported by the narrow distribution of the β-interface lengths and by the absence of proportion between the lengths of the β-interface and the length of their respective whole chain (Fig. 8). To test that possibility, a simple experiment was carried out using the pentamer of the cholera toxin B (CtxB_5_) as a prototype of the β-interfaces (Fig. 1). Conditions to follow the assembly of the CtxB_5_ *in vitro* had been established previously and are indicated in material and methods [40]. Briefly, the native toxin (Fig. 9, lane 2) is acidified for 15 min at room temperature (RT) to lead to its dissociation into monomers (Fig. 9, lane 3). Subsequently, it is neutralized for 15 min at RT, time during which the reassembly into pentamer takes place (Fig. 9, lane 4). In subsequent experiments, 9mer (P1) or/and 8mer (P2) synthetic peptides with sequences corresponding to S1 (^23^ KIFSYTESL ^31^) and S2 (^96^ IAAISMAN ^103^), respectively, of the wild-type CtxB β-interface were added to the neutralizing buffer. The amounts of CtxB reassembled into pentamer under the different conditions, were then compared using SDS-PAGE (Fig. 9). The addition of P1 (Fig. 9, lane 5), of P2 (Fig. 9, lane 6) and of P1 and P2 together (Fig. 9, lane 7) strongly inhibited the reassembly of the toxin into pentamer. This indicates that P1 as well as P2 do interfere with the formation of CtxB-CtxB interfaces. P1 inhibited more than P2 and the mixture P1+P2 inhibited more than P2 but less than P1. Thus P1 and P2 must be reacting together.

## Discussion

As for the α-coiled interfaces, the choice of a common geometry of interfaces proved to be successful in isolating characteristics among the β-interfaces of otherwise unrelated protein oligomers. The results are thus devoid of potential bias introduced when protein interfaces of proteins with similar folds or similar functions are compared. It was also possible to associate geometrical and chemical properties to the identified features. On one hand, this provides an evaluation of the features so their reliability improves. On the other hand, it also gives some rational about the ‘mode of action’ of the features in term of interface formation. Thus, using the CtxB model, the role of the hydrophobic and of the charged residues on the formation of the secondary structure and on the formation of the CtxB β-interface, respectively, can be tested. However, the study entirely focuses on the β-interfaces and as such the results are far from providing a full picture of the parameters involved in the assembly of the whole chains of the dataset. As an illustration, we have seen that the mutations of the central hydrophobic residues of the BB sub-networks have little effect on the secondary structure predictions of the whole length sequences (∼25%) (not shown). The true essence of the results resides in the observation of interdigitated networks in which the interface features are made through strategic positioning of chemical characteristics rather than through drastic chemical modulation. Thus the search of a sequence of an interface cannot be done as the search of a sequence of a biological function (e.g. active site).

In summary, the β-interfaces are made of two interactions sub-networks. One is involving atoms of the main chain (BB sub-networks) and the other is involving atoms from the side chains (SC sub-networks). The characteristics of the BB sub-networks are related to the hydrophobic residues which seem particularly involved in the secondary structures of the β-interfaces. This is well supported by the fact that the hydrophobic residues favored in the β-interfaces (IVMWC) are also favored in intramolecular β-sheet (IVMCW) [34], [45], [46], [47]. Likewise, the hydrophobic residues disfavored in the β-interfaces (AGP) are disfavored in intramolecular β-sheet (AGP) [34], [45], [46], [47]. There are some discrepancies for the leucine and phenylalanine residues which are favored in intramolecular β-sheets but disfavored or neutral in the β-interfaces, respectively. Intriguingly, these two amino acids are enriched in amyloid β-fiber (LIF) [33]. The role of hydrophobic forces in interfaces (dimers) was previously reported but not in connection with the geometry of the interface [21], [48], [49] and for review see [2], [12], [33].

The hydrophobic amino acids of the BB sub-networks are thus devoid of ‘intermolecular’specificity since they are shared with intramolecular interactions.

In contrast, the charged amino acids favored in the SC sub-networks present some specificity. First, intra-molecular β-interactions as well as dimeric β-interfaces are rather depleted in charged residues, apart from arginine for the dimeric interfaces ([21], [32], [33], [45], [46], [50] and for review [2]). On the contrary, in the β-interface side chains, charged residues represent a third of the interfacial amino acids and have only a slight preference for histidine residues. It is interesting that the histidine residue stands out as it is the only amino acid charged under physiological conditions. It is also an amino acid already shown to take part in the assemblies of several protein oligomers [51], [52], [53]. Second, the β-interfaces of our dataset have an average net charge of −0.5 which differs from the one required for the formation of amyloid β-fiber (net charge of ±1), another type of β-interface [54], [55], [56].

The third and most practical information about the charge specificity, resides in the distribution of the charged residues. The arginine residues are frequent at both the corners (N- and C- terminal caps) of the β-interfaces whereas histidine and glutamic acid are favored centrally. Lysine and aspartic acid residues have no preferred position in the β-interfaces.

**Figure 10:**
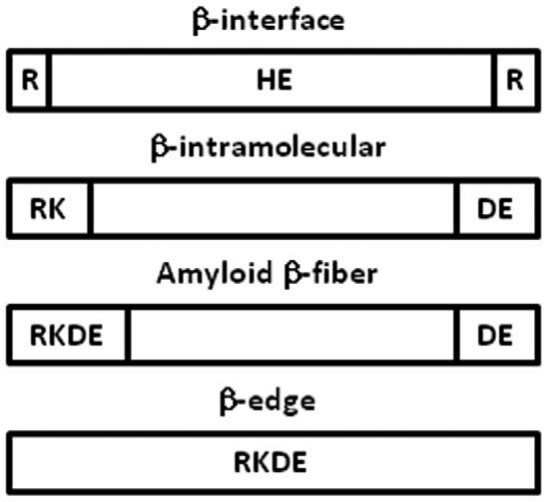
Schematic of the charge distribution in β-interactions. The amino acids are indicated using the single letter code.

This is in contrast to parallel intramolecular β-sheet in which positively charged residues (KR) are located at the N-terminal extremities only and negatively charged residues (DE) are present at the C-terminal extremities only [47]. The presence of charges at the N- or C- terminal extremities is believed to act as β-breakers [45], [47]. Additionally, the formation of amyloid β-fiber is promoted with positively charged residues (KR) located at the N-terminal extremities of the amyloid β-strands and negatively charged residues (DE) at both the N- or C-terminal extremities [54], [55]. Finally, charged residues centrally located are observed in intra-molecular edge β-strands and are thought to prevent their aggregation [34]. Hence, the scattered distribution observed on the β-interfaces differentiates them from other types of intramolecular and intermolecular dimeric β-interactions (Fig. 10).

Altogether the data lead us to propose some hypothesis on the construction mechanism of the β-interfaces following two principles: (i) interfaces are built via geometrical and chemical recognition of the interacting domains and (ii) there are a recognition phase (‘binding’) and a stabilization phase. The BB sub-networks, via the hydrophobic residues, could provide the geometrical recognition whereas the side chain charged residues could provide the chemical one. It is tempting to speculate that the long arginine residue located at the extremities is employed as a hook to promote encounter. The central smaller histidine and glutamic acid residues could act as clips to stabilize the interface. Alternatively, they might, as proposed for the β-edge strands, maintain the two domains soluble prior the recognition.

Some experimental data are consistent with a relation between Gemini's hotspot residues and their involvement in the process of a β-interface formation. For example, the heat labile enterotoxin B (LTB_5_) and the cholera toxin B (CtxB_5_) pentamers, which shares 84% sequence identity and almost superimposable x-ray structures, have nevertheless different assembly mechanisms and different β-interface graphs (1EFI and 1EEI, respectively). The two toxin pentamers have only 14 different amino acids and one of them is in the β-interface (Leu 25 and Phe 25 in 1EFI and 1EEI, respectively). Residue 25 is involved in a I_BB_ in both graphs but leucine and phenylalanine have been measured with different global propensities (Table 5). There are 6 I_BB_ for 4 I_SC_ in LTB_5_ compatible with a geometry-regulated assembly as observed experimentally since only folded LTB chains associate [57]. On the other hand, there are 5 I_BB_ for 5 I_SC_ in CtxB_5_ consistent with a more ‘chemically’-regulated assembly also observed experimentally with partially folded CtxB chains capable of associating [40], [52]. The presence of a I_SC_ involving a lysine residue only in CtxB_5_ (K23-N103) also supports a more ‘chemically’-regulated assembly. Similarly, shiga-like toxin I and II have different stabilities and different graphs (2XSC and not shown) [58]. In the bacterial hexameric (1U1S) from *Pseudomonas aeruginosa* , the mutation of His 57, to alanine (Ala) or to threonine (Thr) destabilizes the hexamer by disturbing the side chain hydrogen bond network of the His 57 with the side chains of Lys 56 and Ile 59 of the adjacent chain [59]. The His 57 side chain hydrogen bond network is properly seen on the Gemini graph of the β-interface of Hfq (Dataset 1, 1U1S). Disappearance of that network (or changes of that network) for mutant Ala 57 (or for mutant Thr 57) is also seen properly on the Gemini graphs of the mutated Hfq (not shown). Moreover, the conserved main chain hydrogen bond network made of the residues Met 53 and Tyr 55 of chain M with the residues Val 62 and Ser 60 of the adjacent chain is also identified by Gemini (not shown) [60]. However, cautious is necessary with interpreting the graph features. At this stage, they should be used as a tool to formulate hypothesizes for experimental tests.

There are several arguments, mentioned in the result section, supporting the idea that the β-interfaces are independent assembly unit. The most indicative one is the experimental observation that the CtxB β-interface peptides recognize the CtxB individual chains. Such peptides could be called “assemblons” by homology to the foldons [61], [62]. Some peptides have been found to lead to the trimerization of proteins when genetically added to their sequence, supporting the ‘assemblons’ concept [63], [64], [65].

## Supporting Information

Dataset S1 **Gemini Graphs of the 40 β-interfaces.** Each graph appears on a separate page. The stœchiometry and the PDB code of the concerned protein oligomer is indicated on the box in the left hand side of the image. The amino acid number is indicated with the type of amino acid at position X. Segments 1 and 2 appear on two parallel rows. X indicates amino acids involved in atomic interactions according to Gemini. SC and BB interactions are illustrated by solid and dashed lines, respectively [15]. The graphs which interfaces have been annotated manually are indicated with a straight line above the segments. A top (left) and a side view (right) of the x-ray structure of the protein oligomer is shown above its respective graph.
(PDF)

Table S1 Features of the protein oligomers of the dataset.
(DOC)

Table S2 Features of the β-interfaces.
(DOC)

Table S3 Properties of the two sub-graphs.
(DOC)

## References

[1] Goodsell DS, Olson AJ (2000). Structural symmetry and protein function.. Annu Rev Biophys Biomol Struct.

[2] Janin J, Bahadur RP, Chakrabarti P (2008). Protein-protein interaction and quaternary structure.. Q Rev Biophys.

[3] Iacovache I, van der Goot FG, Pernot L (2008). Pore formation: An ancient yet complex form of attack.. Biochim Biophys Acta.

[4] Lesieur C, Vecsey-Semjen B, Abrami L, Fivaz M, Gisou van der Goot F (1997). Membrane insertion: The strategies of toxins (review).. Mol Membr Biol.

[5] Kirkitadze MD, Bitan G, Teplow DB (2002). Paradigm shifts in Alzheimer's disease and other neurodegenerative disorders: the emerging role of oligomeric assemblies.. J Neurosci Res.

[6] Soto C (2003). Unfolding the role of protein misfolding in neurodegenerative diseases.. Nature Reviews Neuroscience.

[7] Klein W, Stine W (2004). Small assemblies of unmodified amyloid [beta]-protein are the proximate neurotoxin in Alzheimer's disease.. Neurobiology of aging.

[8] Harrison RS, Sharpe PC, Singh Y, Fairlie DP (2007). Amyloid peptides and proteins in review.. Rev Physiol Biochem Pharmacol.

[9] Miller Y, Ma B, Nussinov R (2010). Polymorphism in Alzheimer A amyloid organization reflects conformational selection in a rugged energy landscape.. Chemical reviews.

[10] Larsen TA, Olson AJ, Goodsell DS (1998). Morphology of protein-protein interfaces.. Structure.

[11] Grueninger D, Treiber N, Ziegler MOP, Koetter JWA, Schulze MS (2008). Designed protein-protein association.. Science.

[12] Tuncbag N, Kar G, Keskin O, Gursoy A, Nussinov R (2009). A survey of available tools and web servers for analysis of protein–protein interactions and interfaces.. Briefings in Bioinformatics.

[13] Cazals F, Proust F, Bahadur RP, Janin J (2006). Revisiting the Voronoi description of protein-protein interfaces.. Protein Sci.

[14] Shulman-Peleg A, Shatsky M, Nussinov R, Wolfson HJ (2007). Spatial chemical conservation of hot spot interactions in protein-protein complexes.. BMC Biol.

[15] Feverati Ga, Lesieur C (2010). Oligomeric Interfaces under the Lens: Gemini.. Plos One.

[16] Guidry JJ, Shewmaker F, Maskos K, Landry S, Wittung-Stafshede P (2003). Probing the interface in a human co-chaperonin heptamer: residues disrupting oligomeric unfolded state identified.. BMC Biochem.

[17] Crick FHC (1953). The packing of alpha-helices: simple coiled-coils.. Acta Crystallogr.

[18] Lupas A, Van Dyke M, Stock J (1991). Predicting coiled coils from protein sequences.. Science.

[19] Lupas A (1996). Coiled coils: new structures and new functions.. Trends Biochem Sci.

[20] Walshaw J, Woolfson DN (2003). Extended knobs-into-holes packing in classical and complex coiled-coil assemblies.. J Struct Biol.

[21] Guharoy M, Chakrabarti P (2007). Secondary structure based analysis and classification of biological interfaces: identification of binding motifs in protein-protein interactions.. Bioinformatics.

[22] Yan C, Wu F, Jernigan RL, Dobbs D, Honavar V (2008). Characterization of protein-protein interfaces.. Protein J.

[23] Davis FP, Sali A (2005). PIBASE: a comprehensive database of structurally defined protein interfaces.. Bioinformatics.

[24] Tsai CJ, Lin SL, Wolfson HJ, Nussinov R (1996). Protein-protein interfaces: architectures and interactions in protein-protein interfaces and in protein cores. Their similarities and differences.. Crit Rev Biochem Mol Biol.

[25] Gao M, Skolnick J (2010). Structural space of protein–protein interfaces is degenerate, close to complete, and highly connected.. Proceedings of the National Academy of Sciences.

[26] Stein A, Mosca R, Aloy P (2011). Three-dimensional modeling of protein interactions and complexes is going omics.. Current opinion in structural biology.

[27] Tsai CJ, Lin SL, Wolfson HJ, Nussinov R (1996). A dataset of protein-protein interfaces generated with a sequence-order-independent comparison technique.. J Mol Biol.

[28] Grigoryan G, Keating AE (2008). Structural specificity in coiled-coil interactions.. Curr Opin Struct Biol.

[29] Calladine CR, Sharff A, Luisi B (2001). How to untwist an alpha-helix: structural principles of an alpha-helical barrel.. J Mol Biol.

[30] Hadley EB, Testa OD, Woolfson DN, Gellman SH (2008). Preferred side-chain constellations at antiparallel coiled-coil interfaces.. Proc Natl Acad Sci U S A.

[31] Tsai CJ, Lin SL, Wolfson HJ, Nussinov R (1997). Studies of protein-protein interfaces: a statistical analysis of the hydrophobic effect.. Protein Sci.

[32] Ma B, Nussinov R (2007). Trp/Met/Phe hot spots in protein-protein interactions: potential targets in drug design.. Current topics in medicinal chemistry.

[33] Ma B, Elkayam T, Wolfson H, Nussinov R (2003). Protein–protein interactions: structurally conserved residues distinguish between binding sites and exposed protein surfaces.. Proceedings of the National Academy of Sciences.

[34] Richardson JS, Richardson DC (2002). Natural -sheet proteins use negative design to avoid edge-to-edge aggregation.. Proceedings of the National Academy of Sciences.

[35] Krishnan A, Zbilut JP, Tomita M, Giuliani A (2008). Proteins as networks: usefulness of graph theory in protein science.. Curr Protein Pept Sci.

[36] Bode C, Kovacs IA, Szalay MS, Palotai R, Korcsmaros T (2007). Network analysis of protein dynamics.. FEBS Lett.

[37] Martin AJ, Vidotto M, Boscariol F, Di Domenico T, Walsh I (2011). RING: networking interacting residues, evolutionary information and energetics in protein structures.. Bioinformatics.

[38] Penel S, Hughes E, Doig AJ (1999). Side-chain structures in the first turn of the alpha-helix.. J Mol Biol.

[39] Laemli U (1970). Cleavage of structural proteins during the assembly of the head of bacteriophage T4.. Nature.

[40] Lesieur C, Cliff MJ, Carter R, James RF, Clarke AR (2002). A kinetic model of intermediate formation during assembly of cholera toxin B-subunit pentamers.. J Biol Chem.

[41] Guex N, Peitsch MC (1997). SWISS-MODEL and the Swiss-PdbViewer: an environment for comparative protein modeling.. Electrophoresis.

[42] Murzin AG, Brenner SE, Hubbard T, Chothia C (1995). SCOP: a structural classification of proteins database for the investigation of sequences and structures.. J Mol Biol.

[43] Lo Conte L, Chothia C, Janin J (1999). The atomic structure of protein-protein recognition sites.. J Mol Biol.

[44] Ma B, Nussinov R (2000). Molecular dynamics simulations of a [beta]-hairpin fragment of protein G: balance between side-chain and backbone forces1.. Journal of molecular biology.

[45] Garratt RC, Thornton JM, Taylor WR (1991). An extension of secondary structure prediction towards the production of tertiary structure.. FEBS letters.

[46] Minor DL, Kim P (1994). Measurement of the b-sheet-forming propensities of amino acids.. Nature.

[47] FarzadFard F, Gharaei N, Pezeshk H, Marashi SA (2008). [beta]-Sheet capping: Signals that initiate and terminate [beta]-sheet formation.. Journal of structural biology.

[48] Merkel JS, Sturtevant JM, Regan L (1999). Sidechain interactions in parallel [beta] sheets: the energetics of cross-strand pairings.. Structure.

[49] Chakrabarti P, Janin J (2002). Dissecting protein-protein recognition sites.. Proteins.

[50] Jones S, Thornton JM (1996). Principles of protein-protein interactions.. Proc Natl Acad Sci U S A.

[51] Tacnet P, Cheong EC, Goeltz P, Ghebrehiwet B, Arlaud GJ (2008). Trimeric reassembly of the globular domain of human C1q.. Biochim Biophys Acta.

[52] Zrimi J, Ng Ling A, Giri-Rachman Arifin E, Feverati G, Lesieur C (2010). Cholera toxin B subunits assemble into pentamers - proposition of a fly-casting mechanism.. PLoS One.

[53] Dang LT, Purvis AR, Huang RH, Westfield LA, Sadler JE (2011). Phylogenetic and functional analysis of histidine residues essential for pH-dependent multimerization of von Willebrand factor.. Journal of Biological Chemistry.

[54] Lopez De La Paz M, Goldie K, Zurdo J, Lacroix E, Dobson CM (2002). De novo designed peptide-based amyloid fibrils.. Proc Natl Acad Sci U S A.

[55] López De La Paz M, Serrano L (2004). Sequence determinants of amyloid fibril formation.. Proceedings of the National Academy of Sciences of the United States of America.

[56] Marshall KE, Serpell LC (2009). Structural integrity of beta-sheet assembly.. Biochem Soc Trans.

[57] Ruddock LW, Coen JJ, Cheesman C, Freedman RB, Hirst TR (1996b). Assembly of the B subunit pentamer of Escherichia coli heat-labile enterotoxin. Kinetics and molecular basis of rate-limiting steps in vitro.. J Biol Chem.

[58] Conrady DG, Flagler MJ, Friedmann DR, Vander Wielen BD, Kovall RA (2010). Molecular basis of differential B-pentamer stability of Shiga toxins 1 and 2.. PLoS One.

[59] Moskaleva O, Melnik B, Gabdulkhakov A, Garber M, Nikonov S (2010). The structures of mutant forms of Hfq from Pseudomonas aeruginosa reveal the importance of the conserved His57 for the protein hexamer organization.. Acta Crystallographica Section F: Structural Biology and Crystallization Communications.

[60] Nikulin A, Stolboushkina E, Perederina A, Vassilieva I, Blaesi U (2005). Structure of Pseudomonas aeruginosa Hfq protein.. Acta Crystallographica Section D: Biological Crystallography.

[61] Panchenko AR, Luthey-Schulten Z, Wolynes PG (1996). Foldons, protein structural modules, and exons.. Proc Natl Acad Sci U S A.

[62] Panchenko AR, Luthey-Schulten Z, Cole R, Wolynes PG (1997). The foldon universe: a survey of structural similarity and self-recognition of independently folding units.. J Mol Biol.

[63] Mitraki A, van Raaij MJ (2005). Folding of beta-structured fibrous proteins and self-assembling peptides.. Methods Mol Biol.

[64] Papanikolopoulou K, Teixeira S, Belrhali H, Forsyth VT, Mitraki A (2004a). Adenovirus fibre shaft sequences fold into the native triple beta-spiral fold when N-terminally fused to the bacteriophage T4 fibritin foldon trimerisation motif.. J Mol Biol.

[65] Papanikolopoulou K, Forge V, Goeltz P, Mitraki A (2004b). Formation of highly stable chimeric trimers by fusion of an adenovirus fiber shaft fragment with the foldon domain of bacteriophage t4 fibritin.. J Biol Chem.

[66] Zhang RG, Scott DL, Westbrook ML, Nance S, Spangler BD (1995). The three-dimensional crystal structure of cholera toxin.. J Mol Biol.

[67] Shomura Y, Yoshida T, Iizuka R, Maruyama T, Yohda M (2004). Crystal structures of the group II chaperonin from Thermococcus strain KS-1: steric hindrance by the substituted amino acid, and inter-subunit rearrangement between two crystal forms.. J Mol Biol.

[68] Gan L, Seyedsayamdost MR, Shuto S, Matsuda A, Petsko GA (2003). The immunosuppressive agent mizoribine monophosphate forms a transition state analogue complex with inosine monophosphate dehydrogenase.. Biochemistry.

