# Supplementary material for: Beta-Strand Interfaces of Non-Dimeric Protein Oligomers Are Characterized by Scattered Charged Residue Patterns
Source: PLoS One. 2012 Apr 9;7(4):e32558. doi: 10.1371/journal.pone.0032558 (PMC3322119; doi:10.1371/journal.pone.0032558)
Supplement: Dataset S1 — Gemini Graphs of the 40 β-interfaces. Each graph appears on a separate page. The stœchiometry and the PDB code of the concerned protein oligomer is indicated on the box in the left hand side of the image. The amino acid number is indicated with the type of amino acid at position X. Segments 1 and 2 appear on two parallel rows. X indicates amino acids involved in atomic interactions according to Gemini. SC and BB interactions are illustrated by solid and dashed lines, respectively [15]. The graphs which interfaces have been annotated manually are indicated with a straight line above the segments. A top (left) and a side view (right) of the x-ray structure of the protein oligomer is shown above its respective graph. (PDF) [file pone.0032558.s001.pdf]

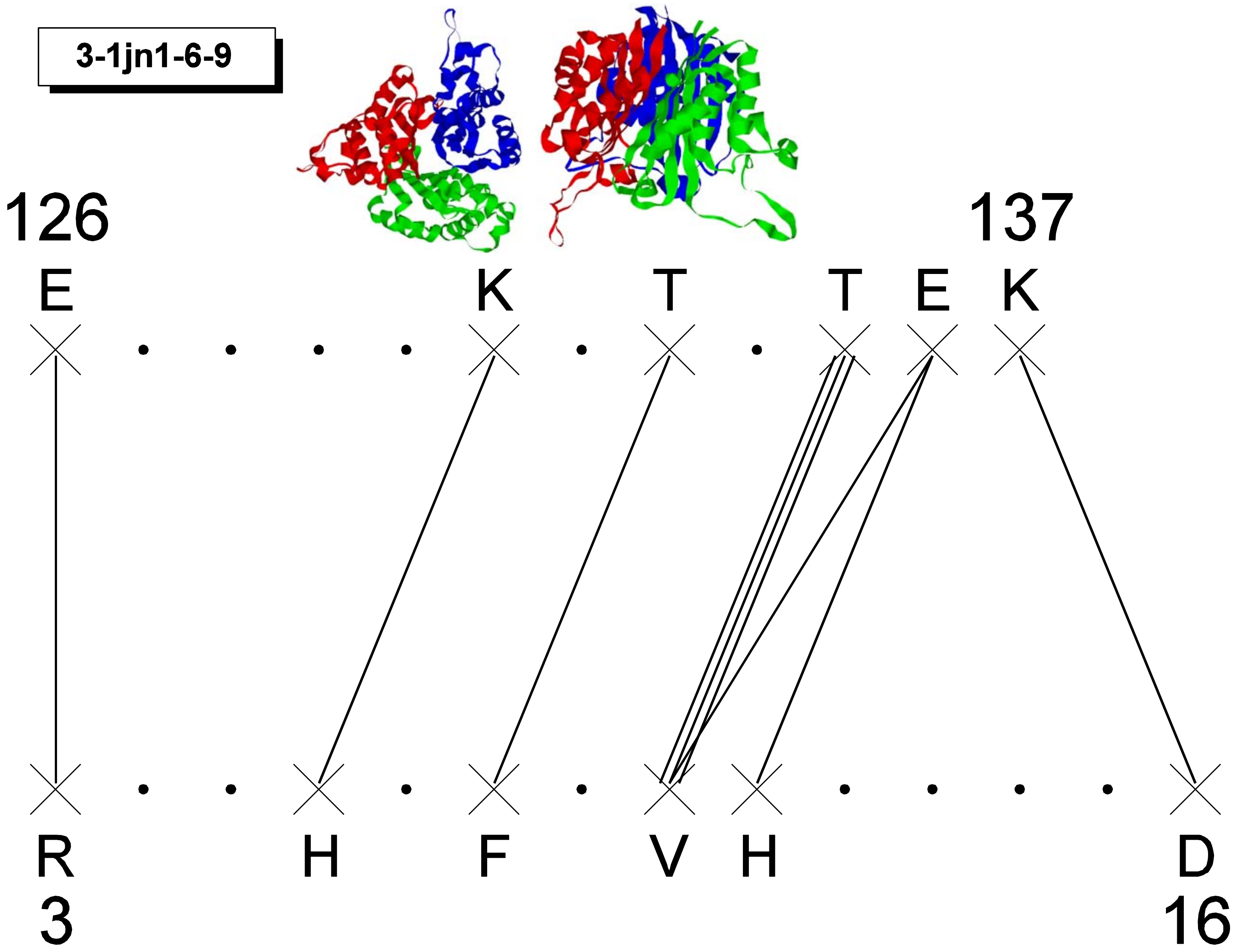
3-1jn1-6-9
126
137
E K T T E K
R H F V H D
3
16

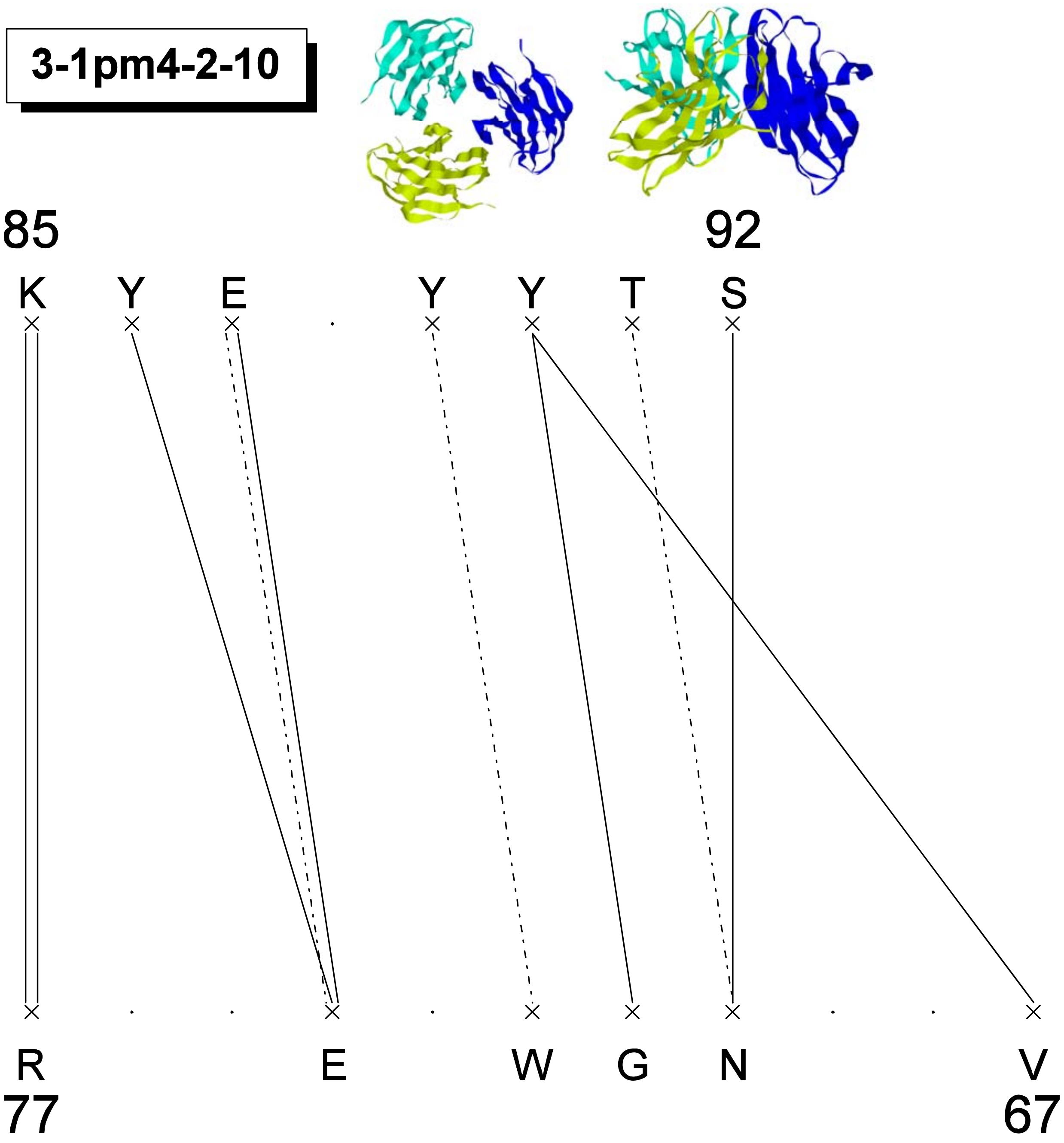
3-1pm4-2-10
85
92
K Y E Y Y T S
R E W G N V
77
67

3-1sjn-1-12

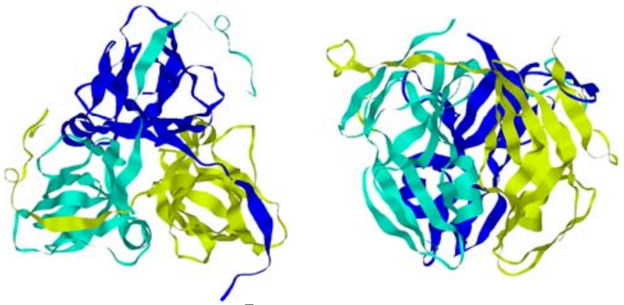

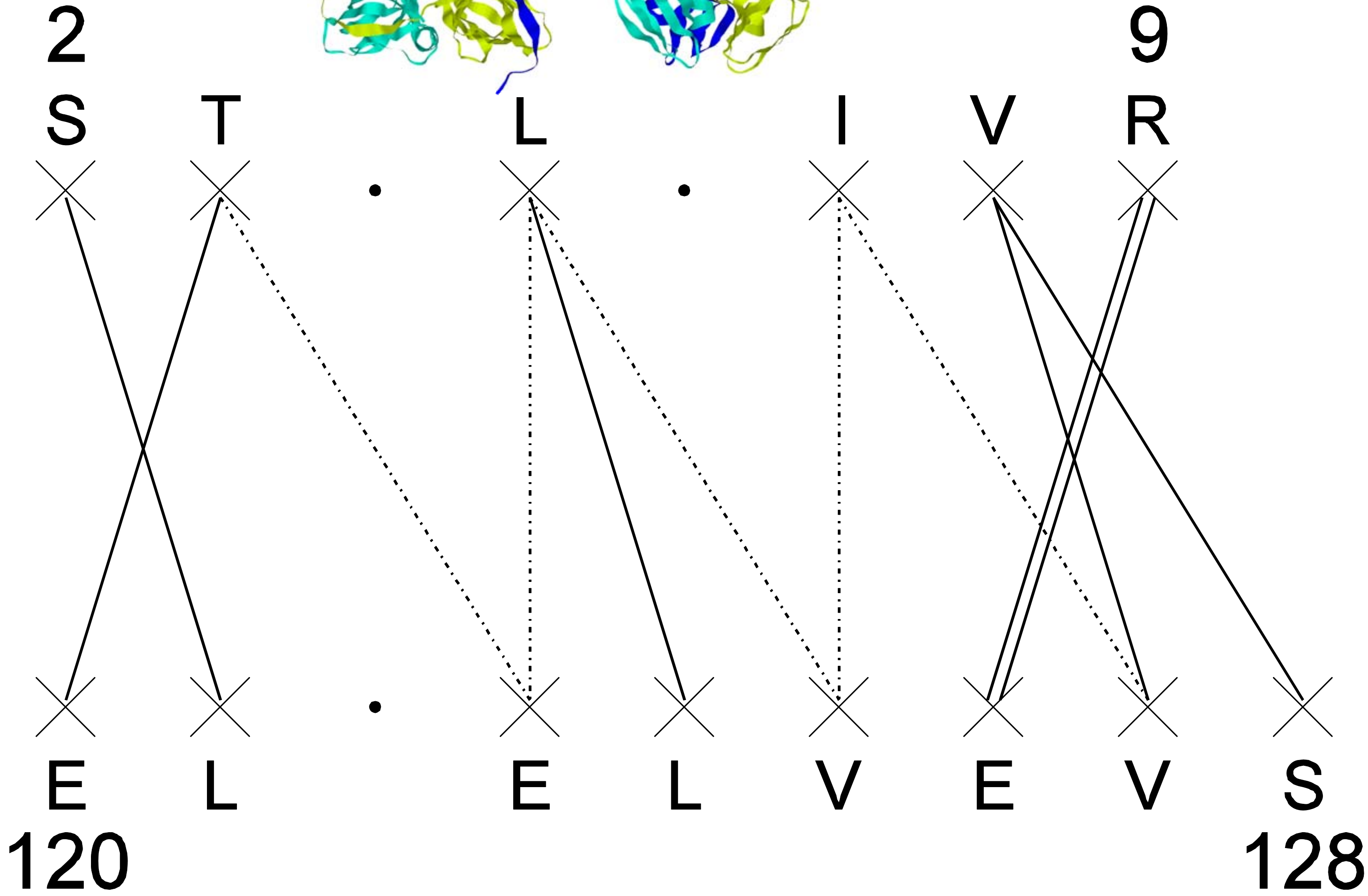

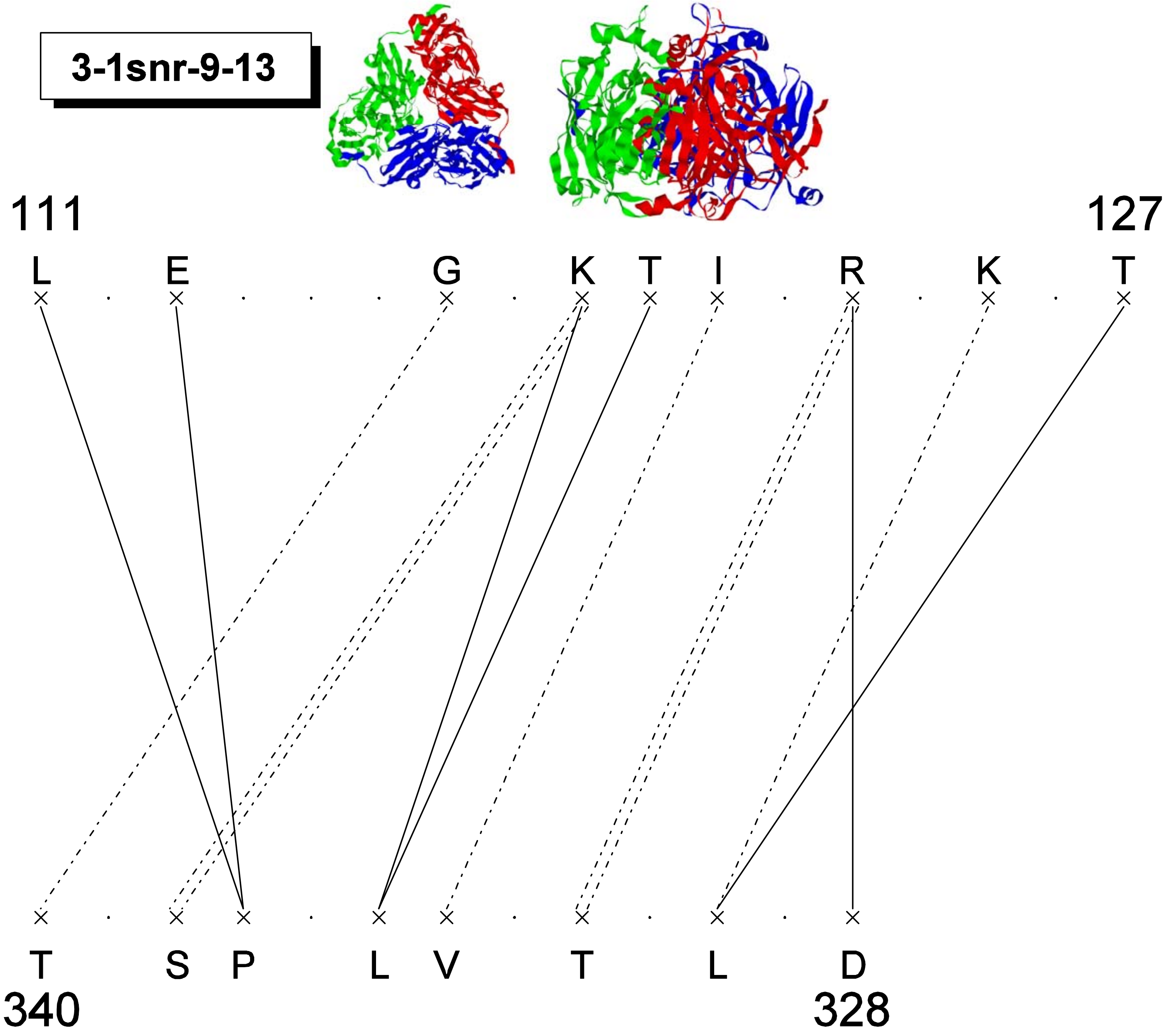
3-1snr-9-13
111
127
L E G K T I R K T
T S P L V T L D
340
328

3-1t0a-2-12

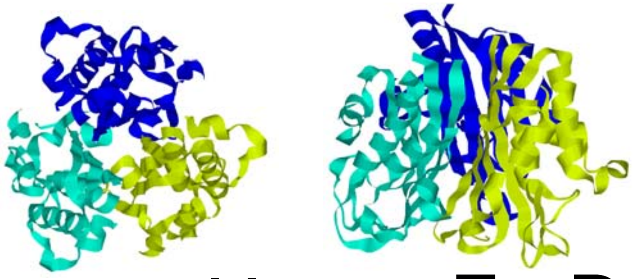

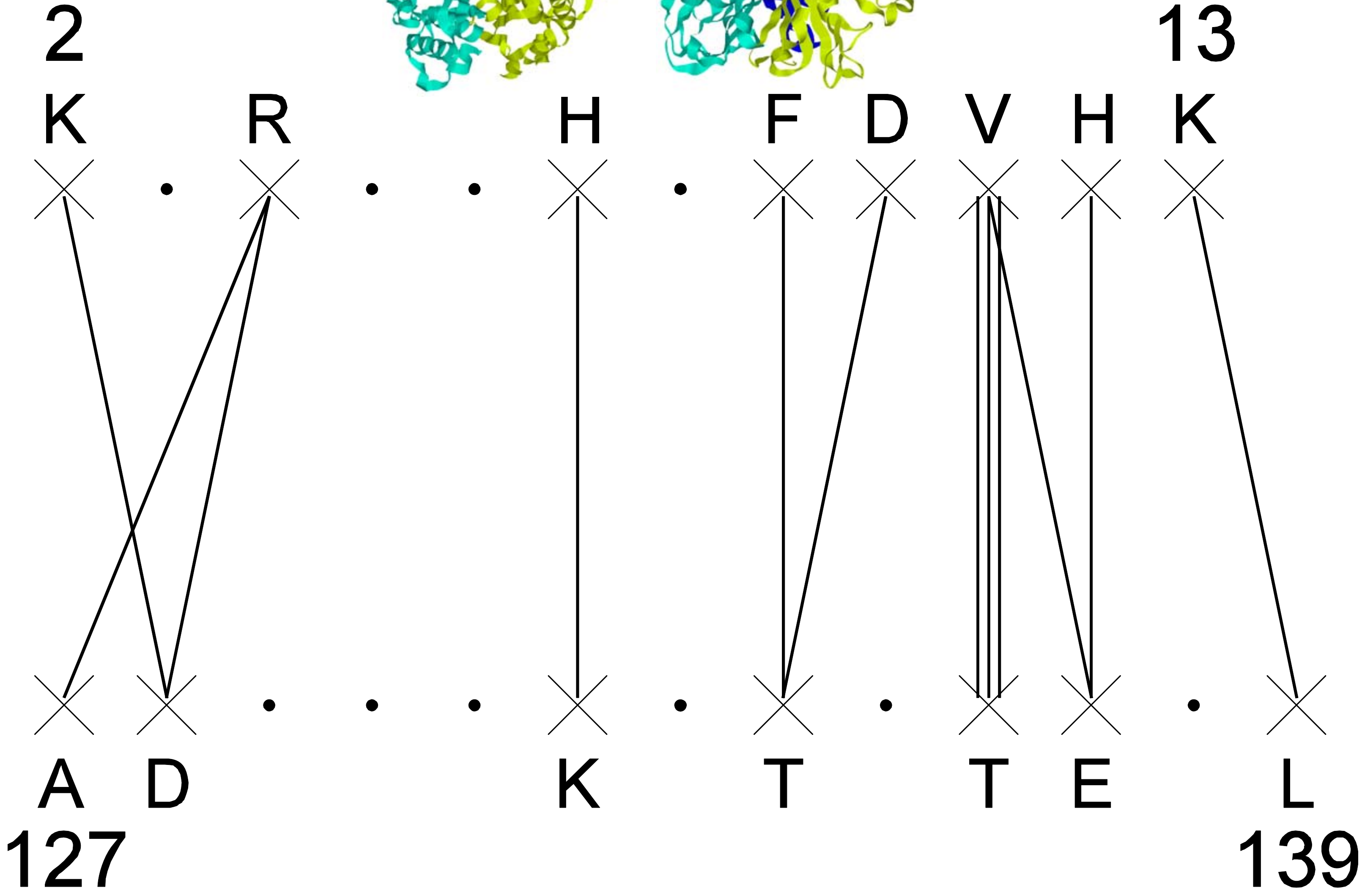

3-1y13-1-14

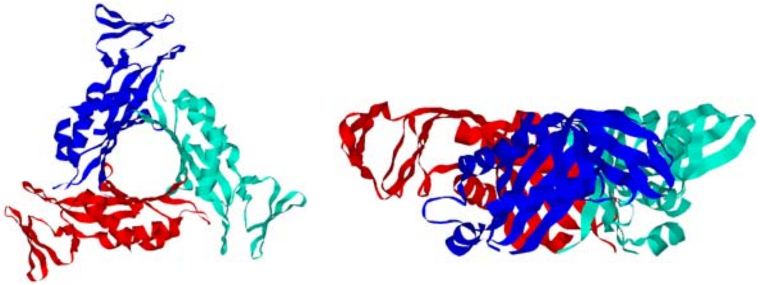

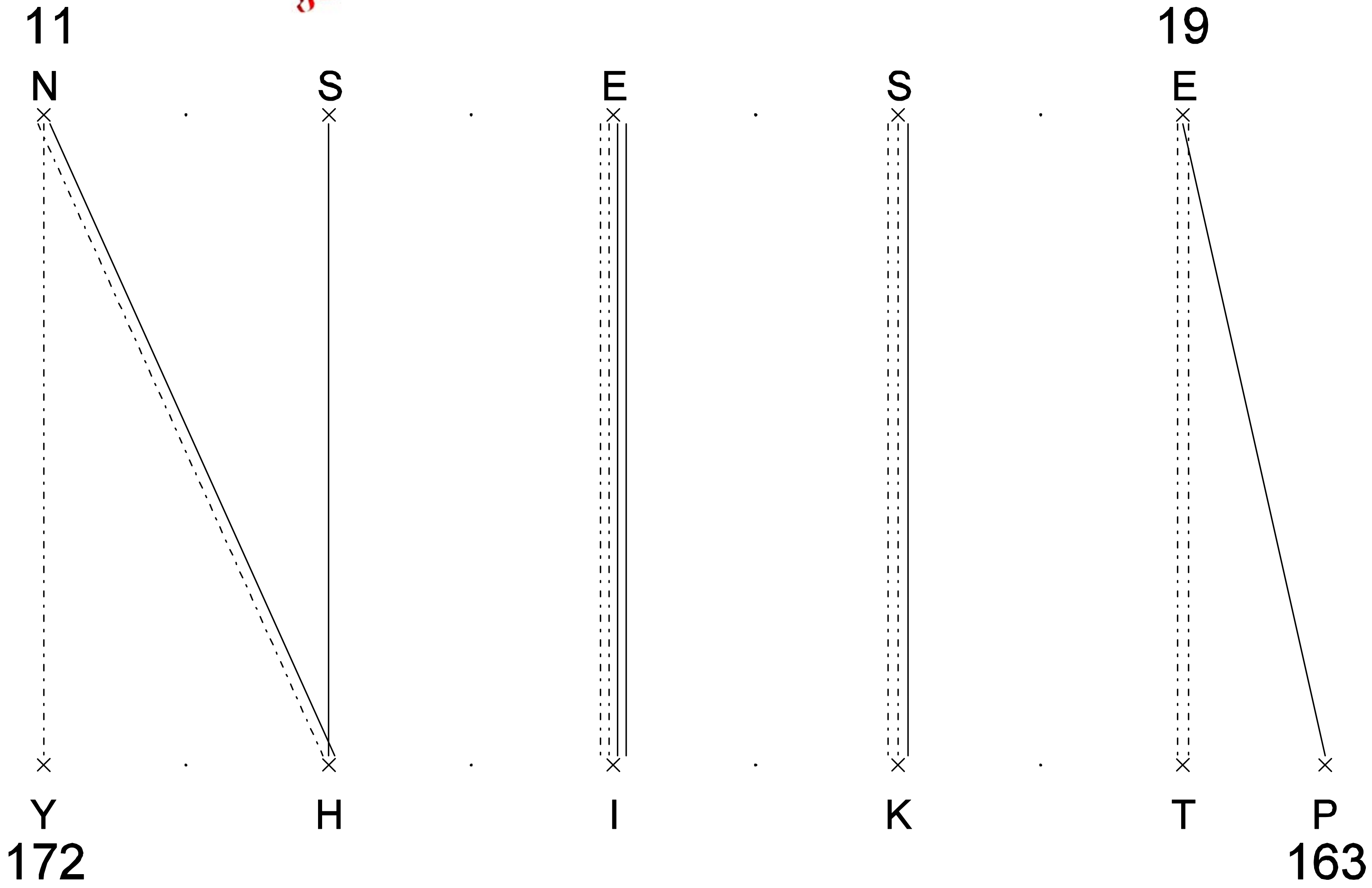

3-2baz-1-9

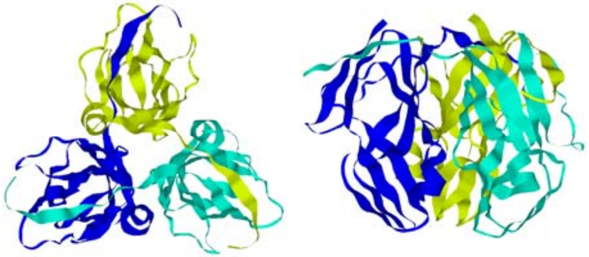

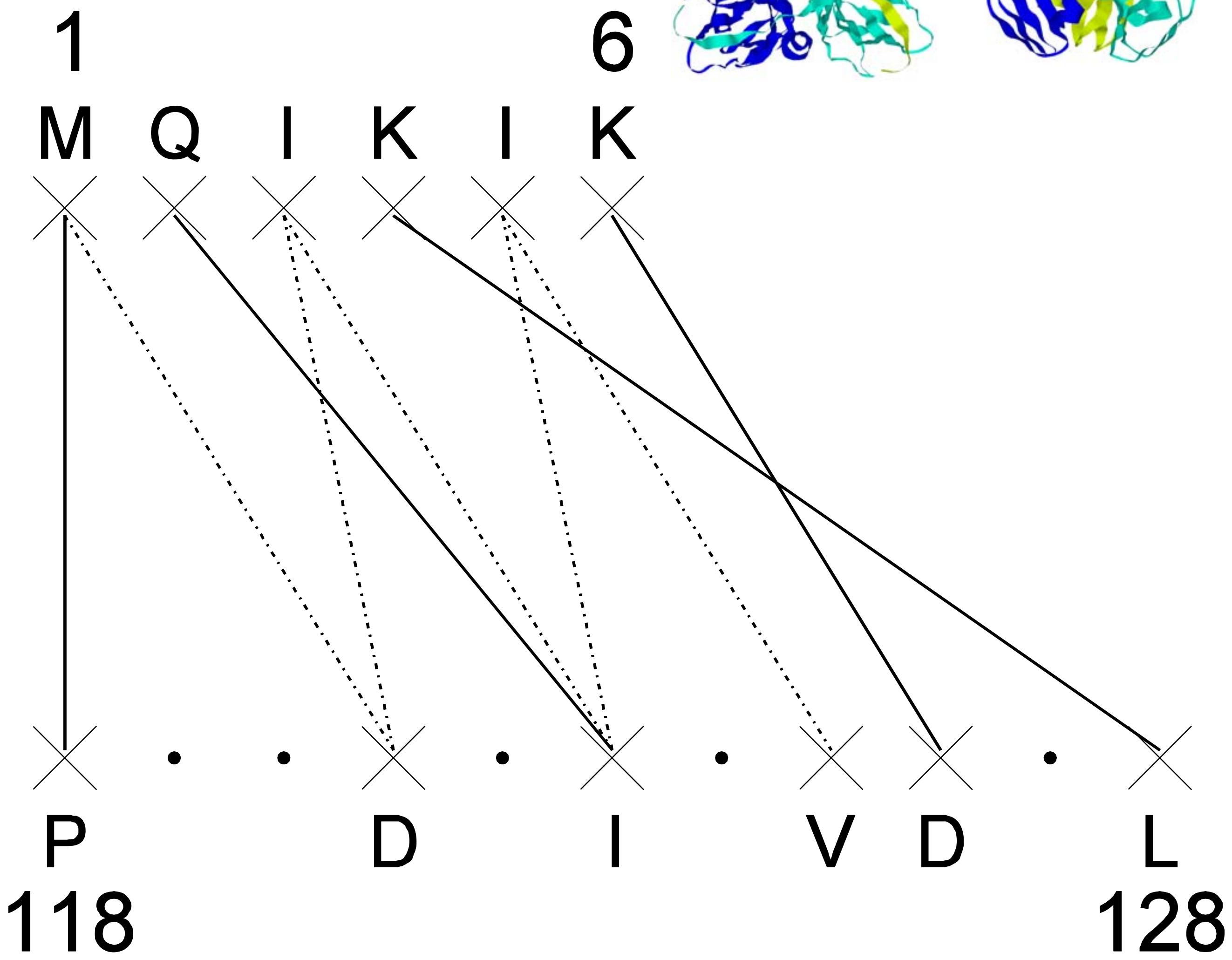

3-2bcm-2-11

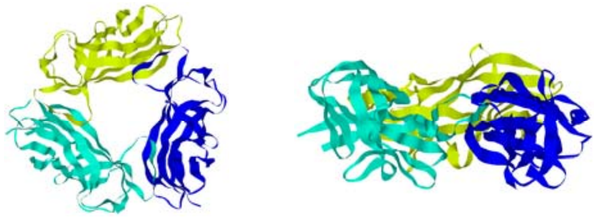

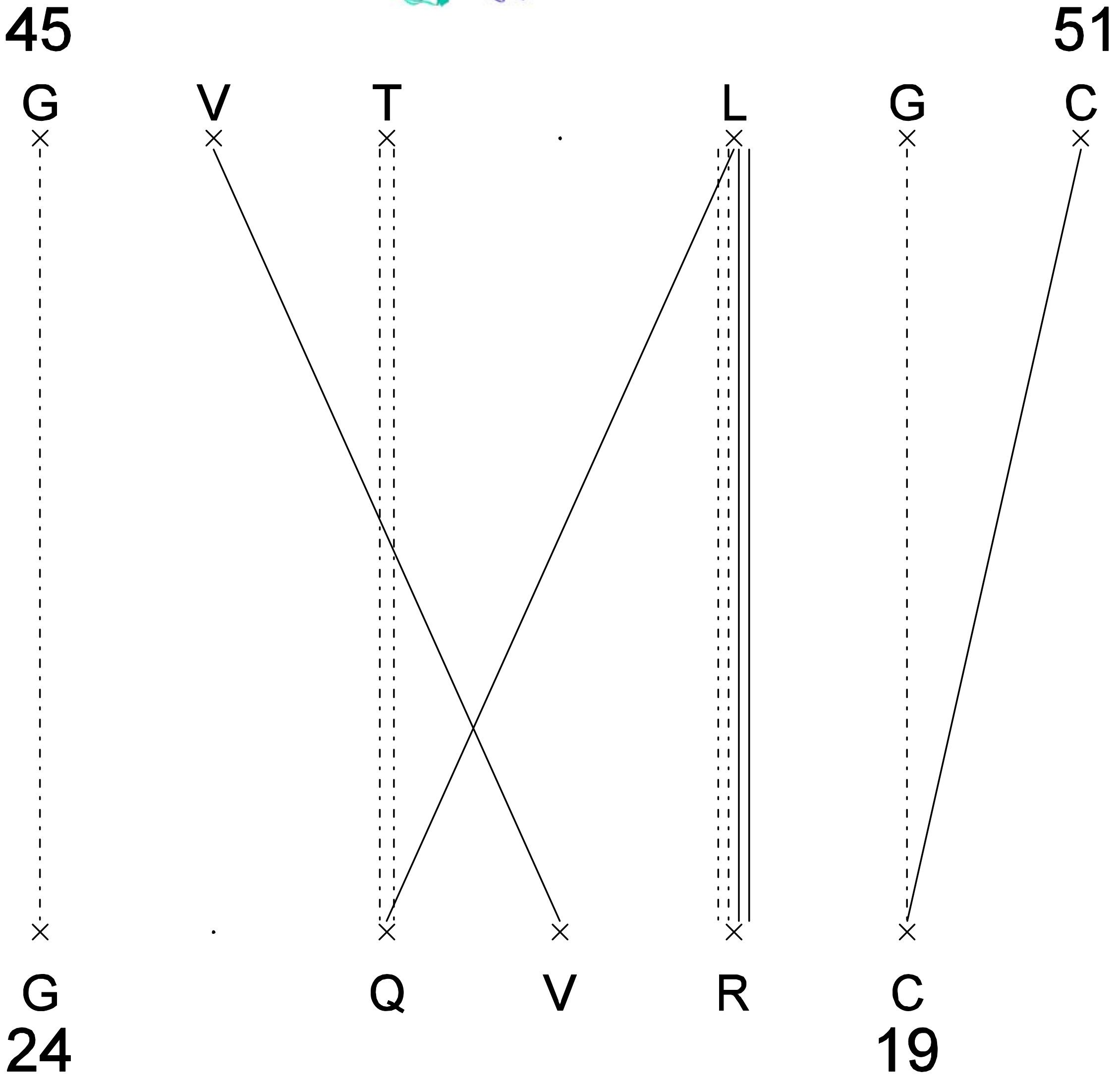

3-2bt9-1-11

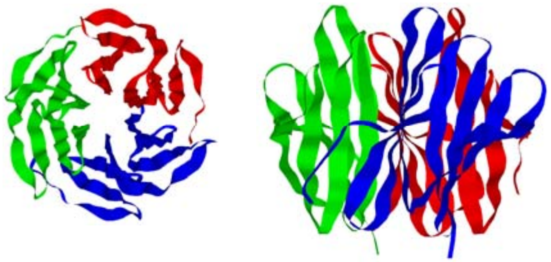

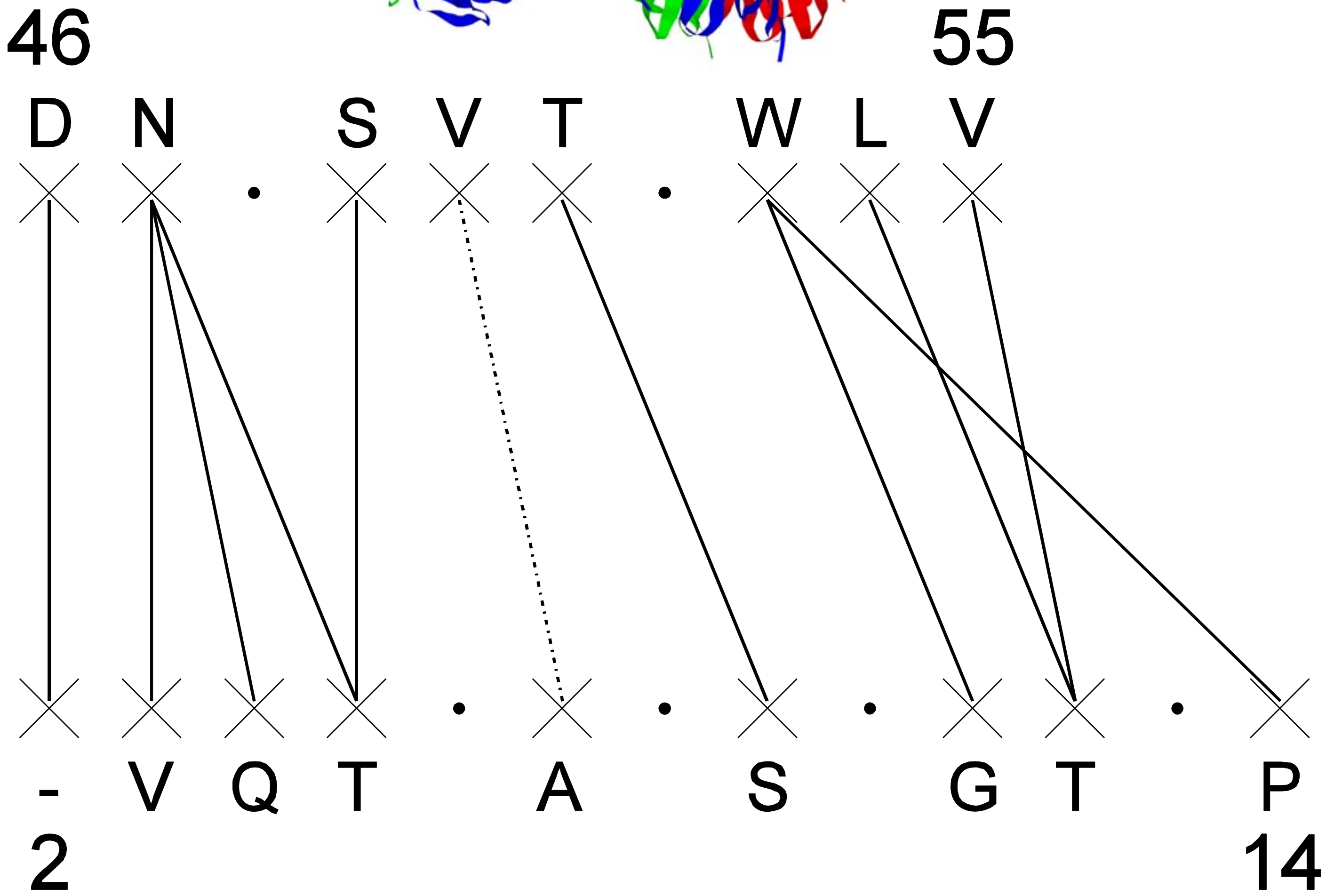

3-2gvh-5-16

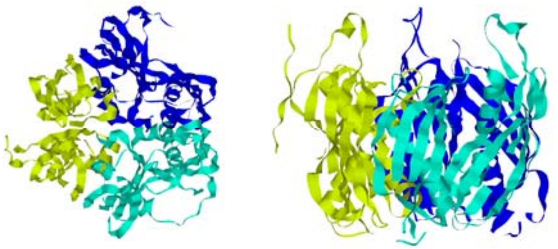

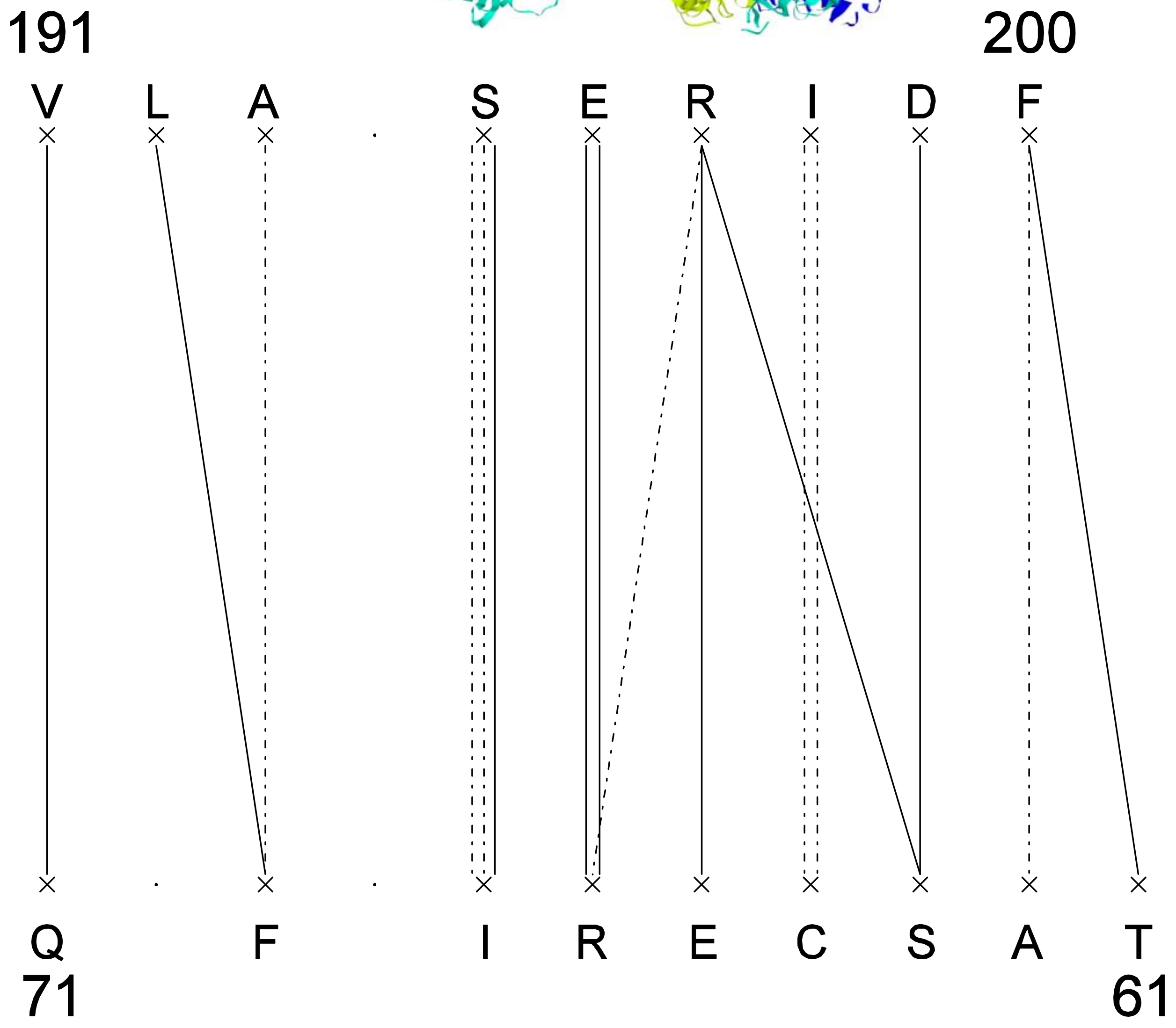

3-2i9d-5-19

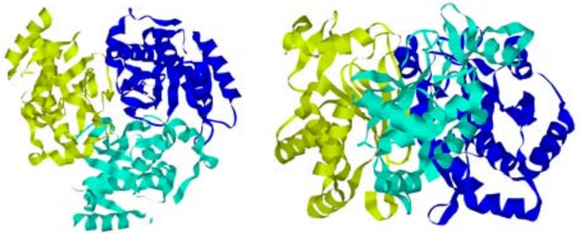

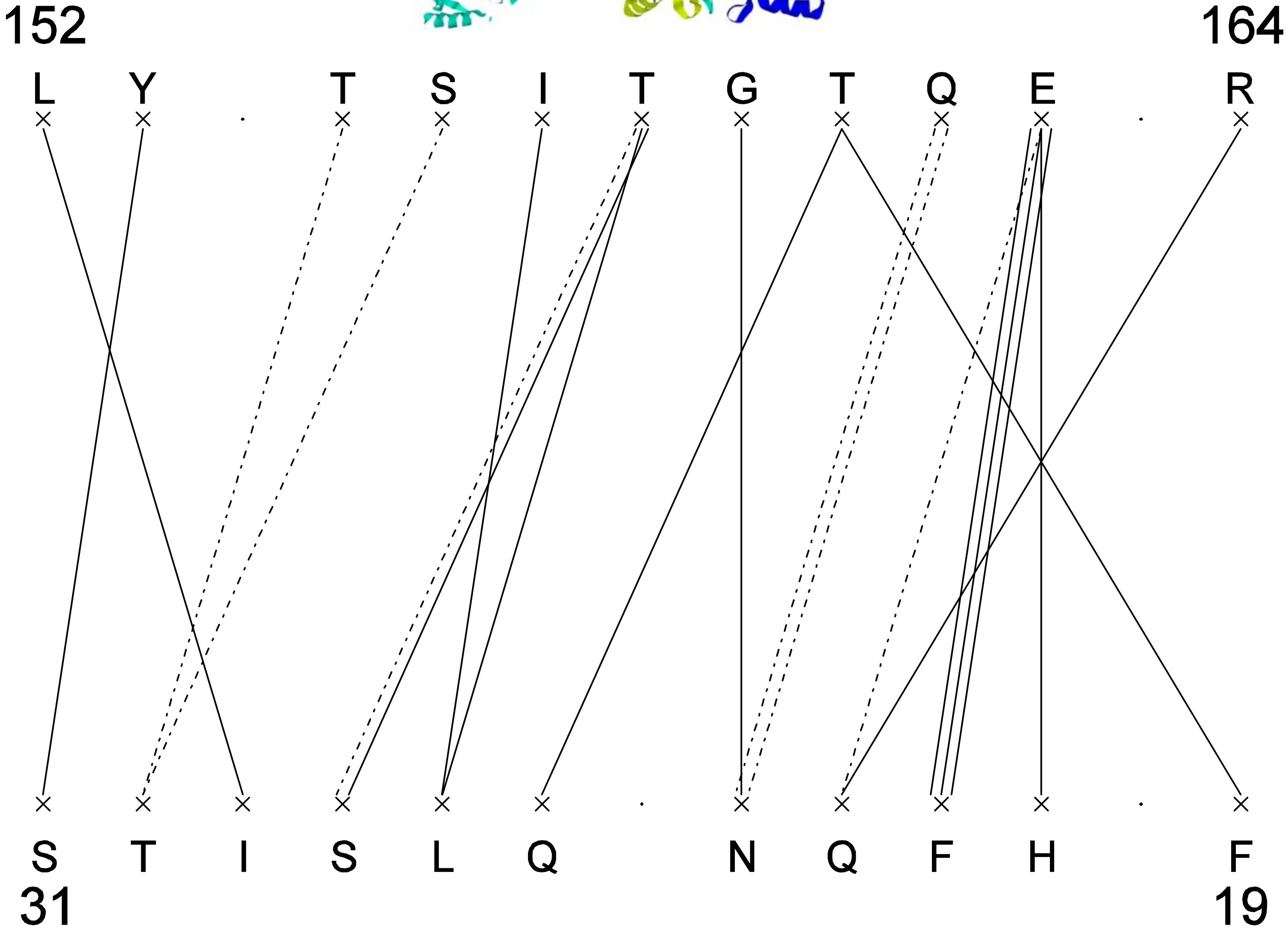

3-2jca-1-16

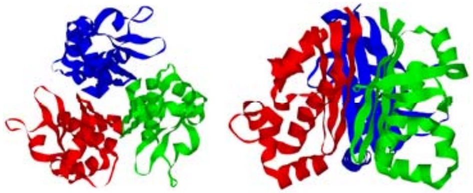

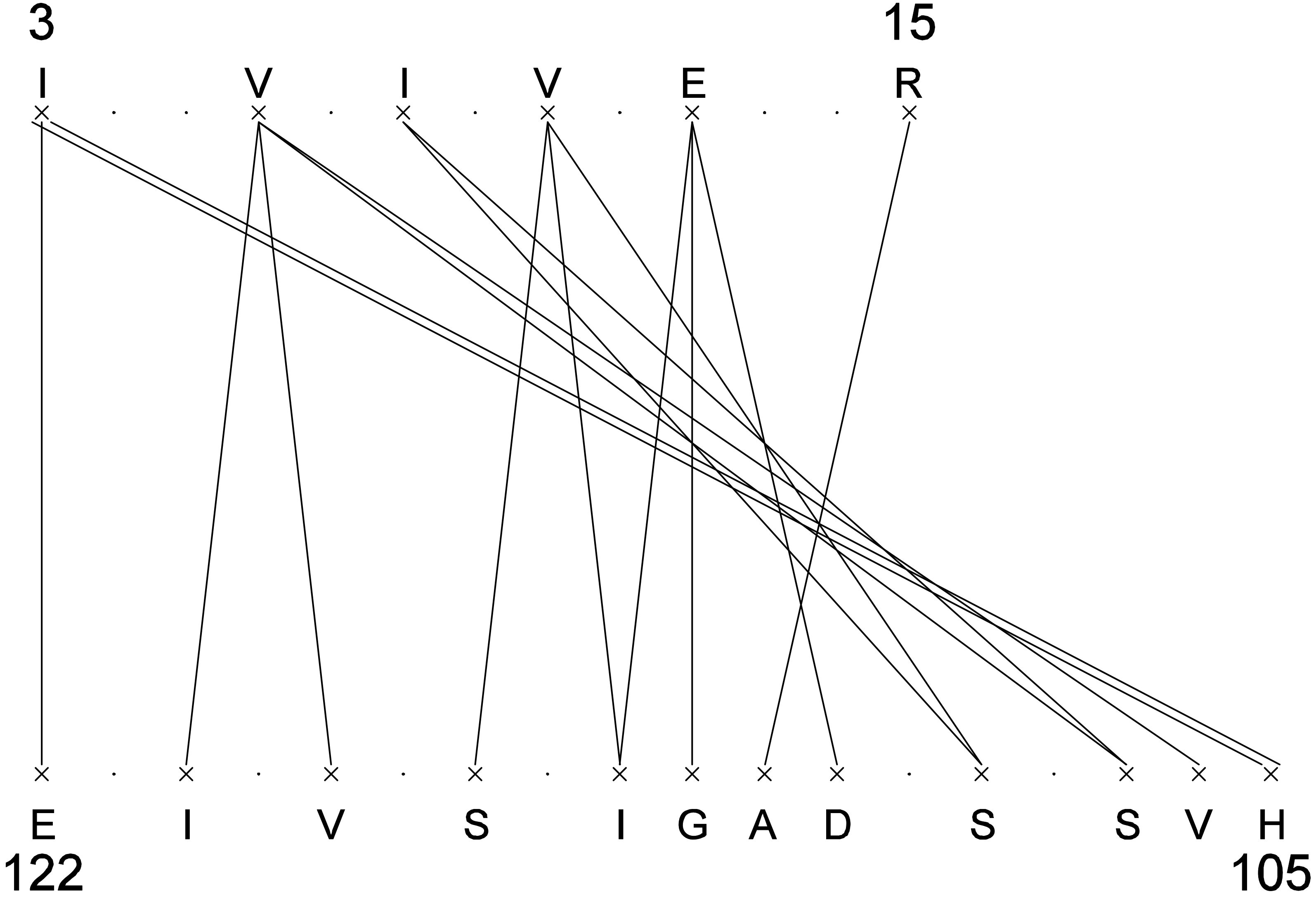

3-2p90-1-18

73 79

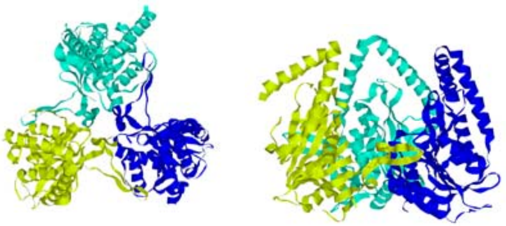

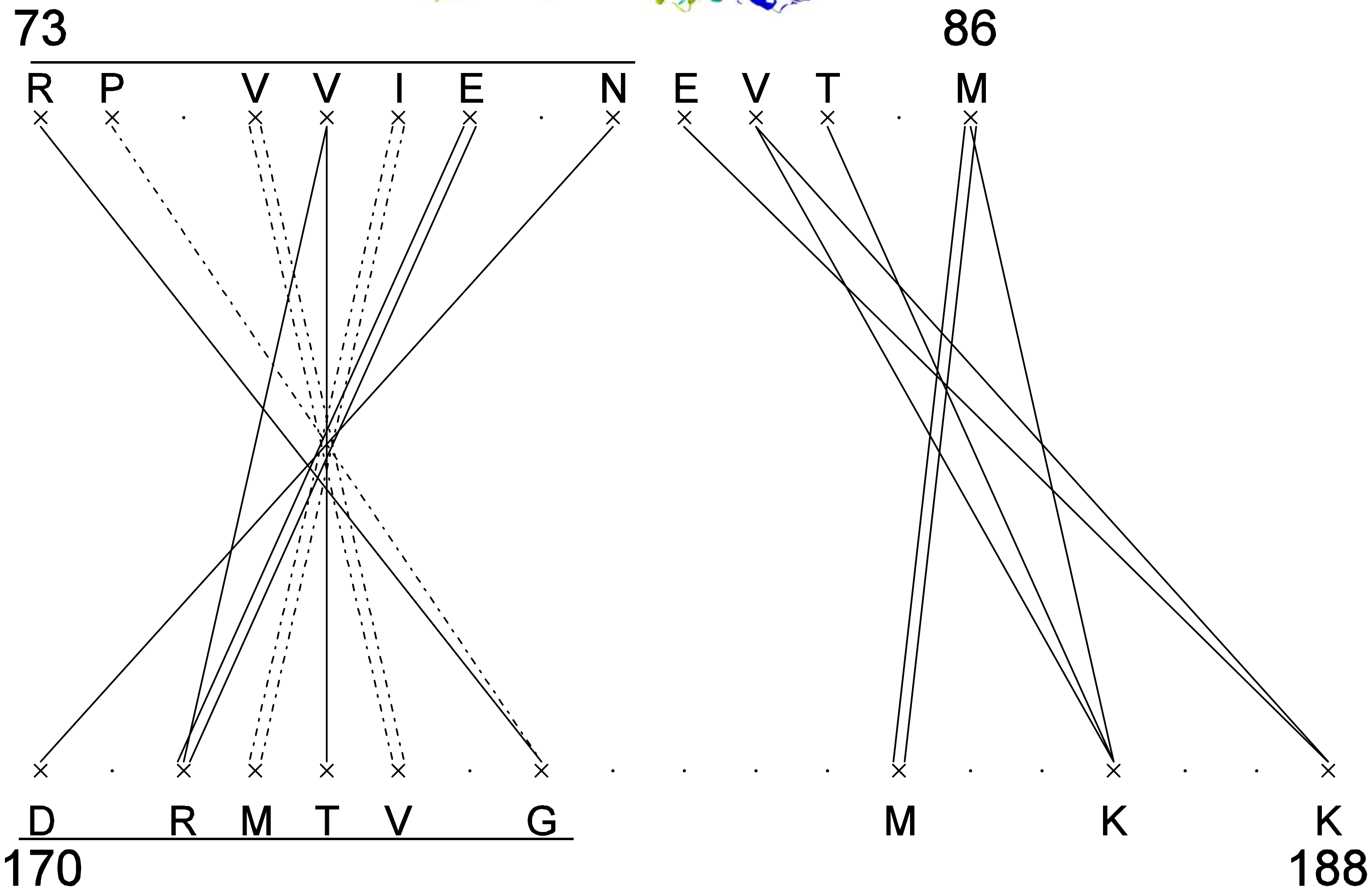

4-1J8D-2-10

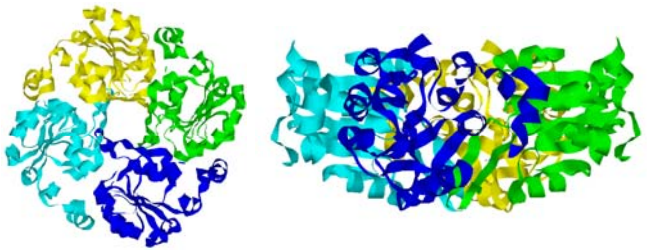

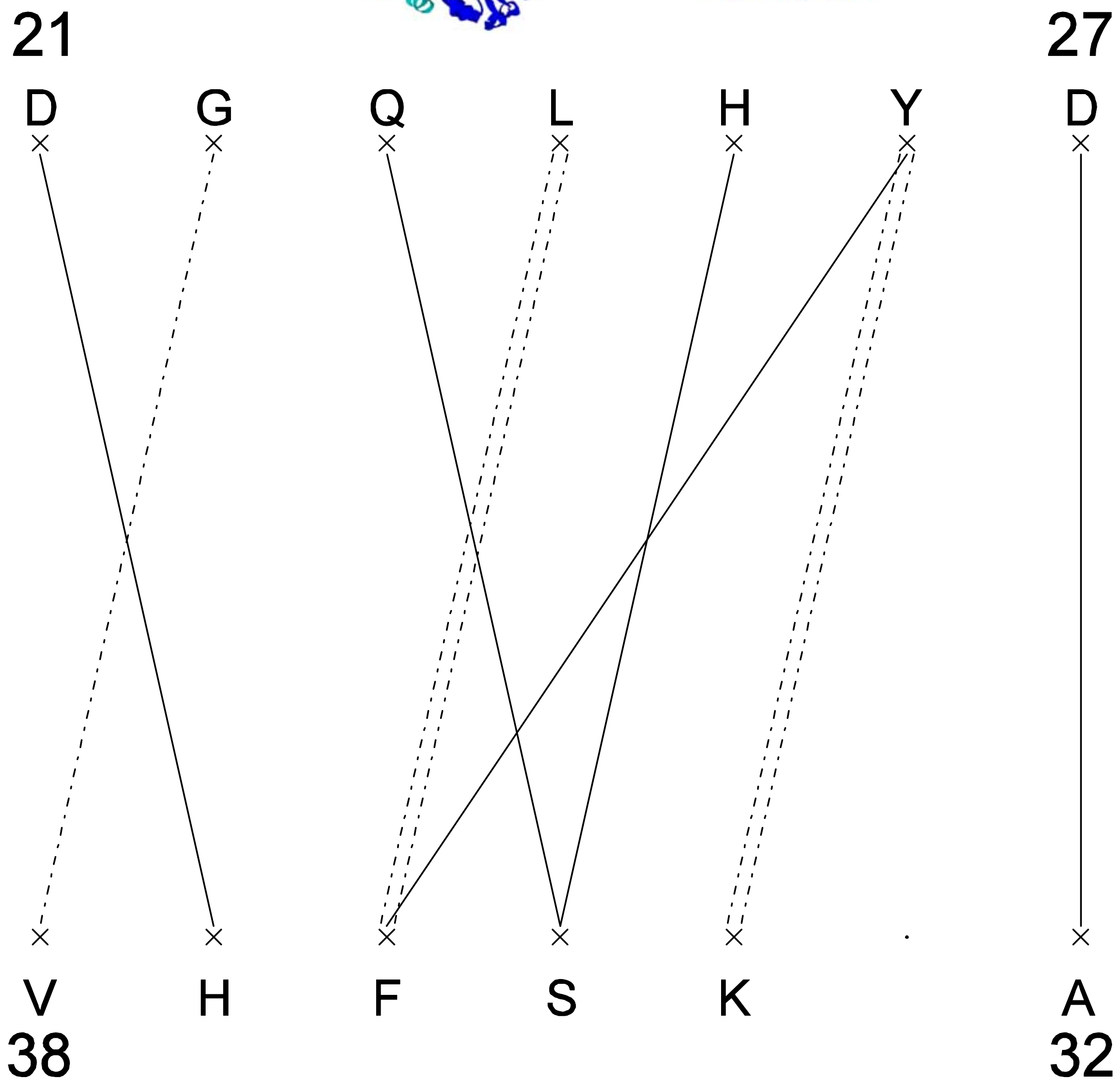

4-1L3A-1-6

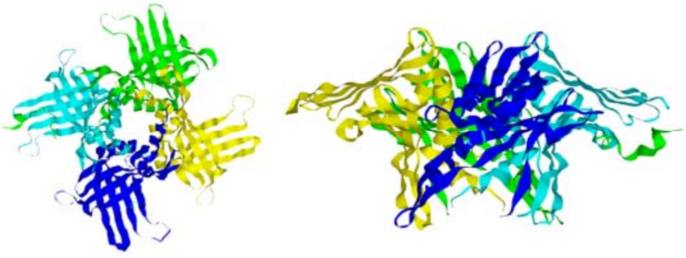

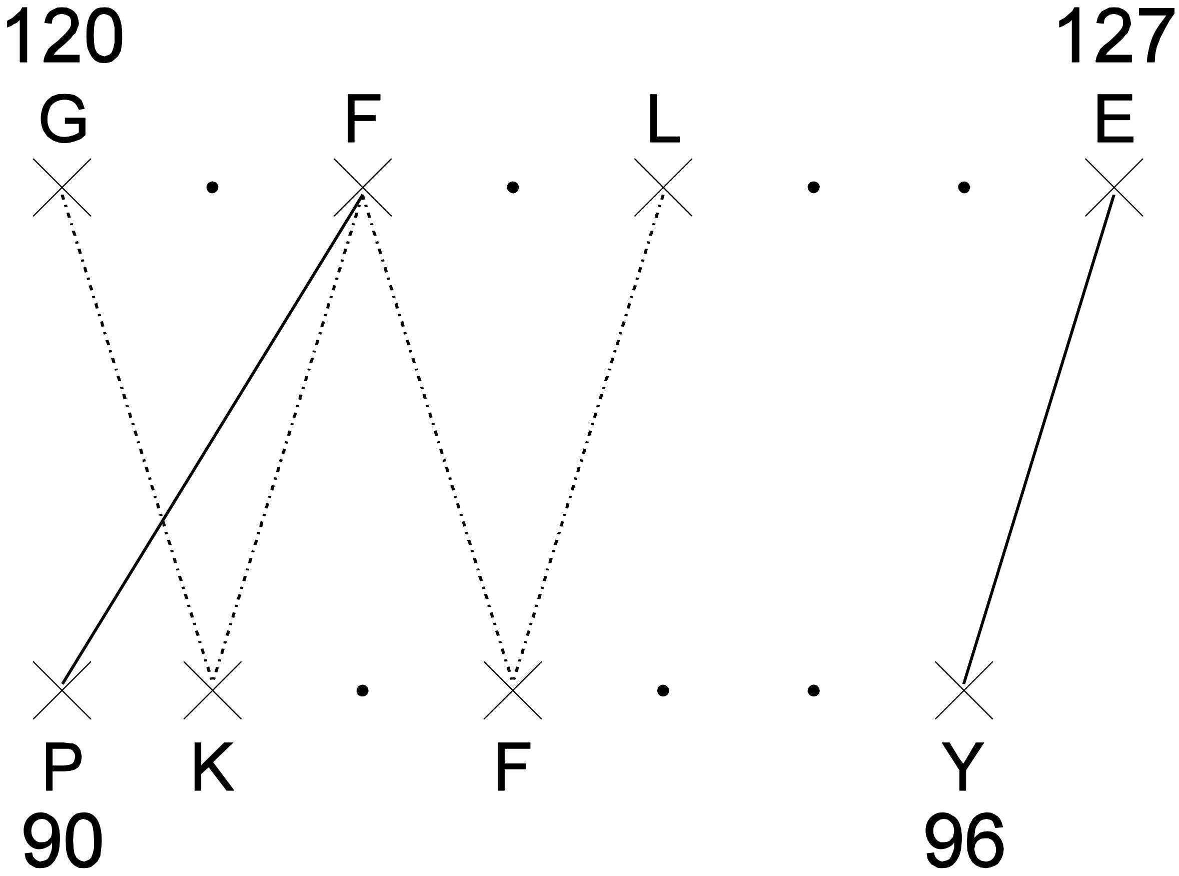

4-1PVN-6-5

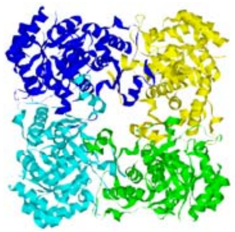

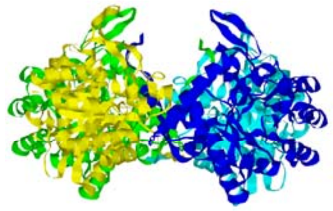

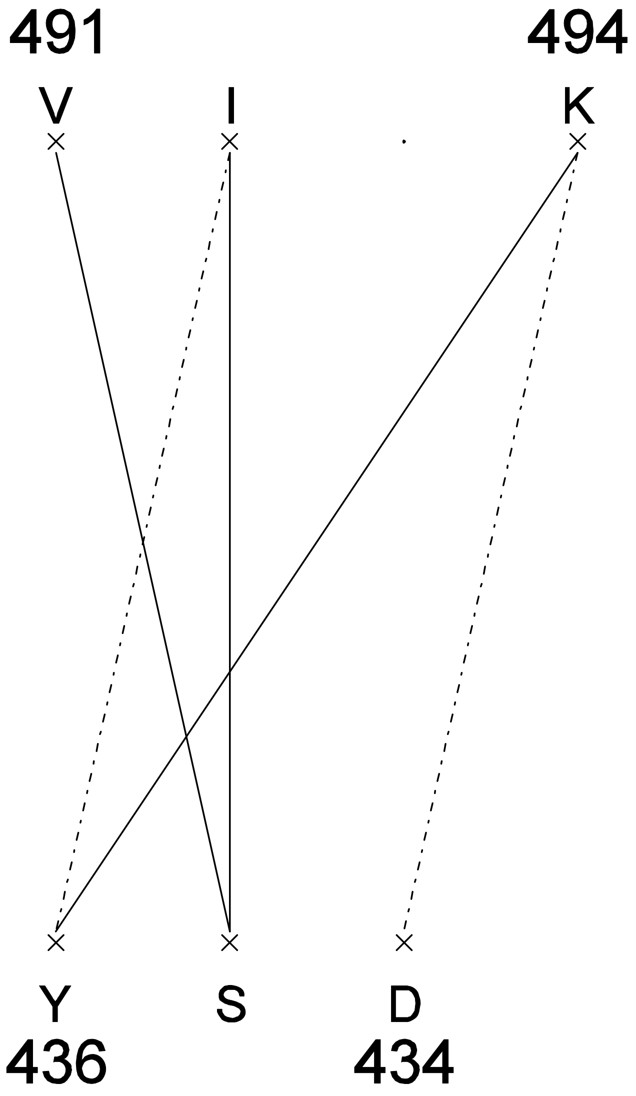

4-2A7R-1-8

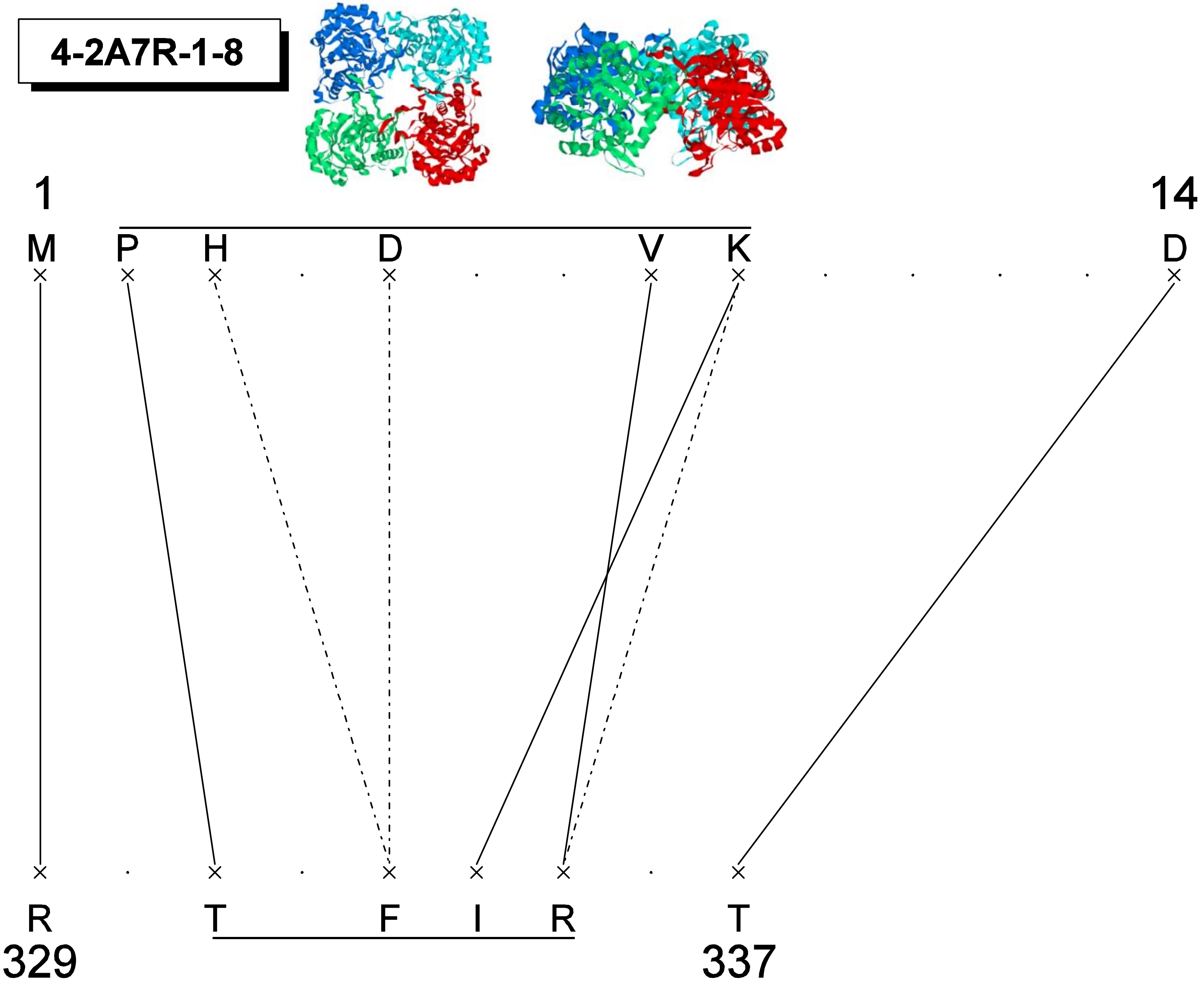

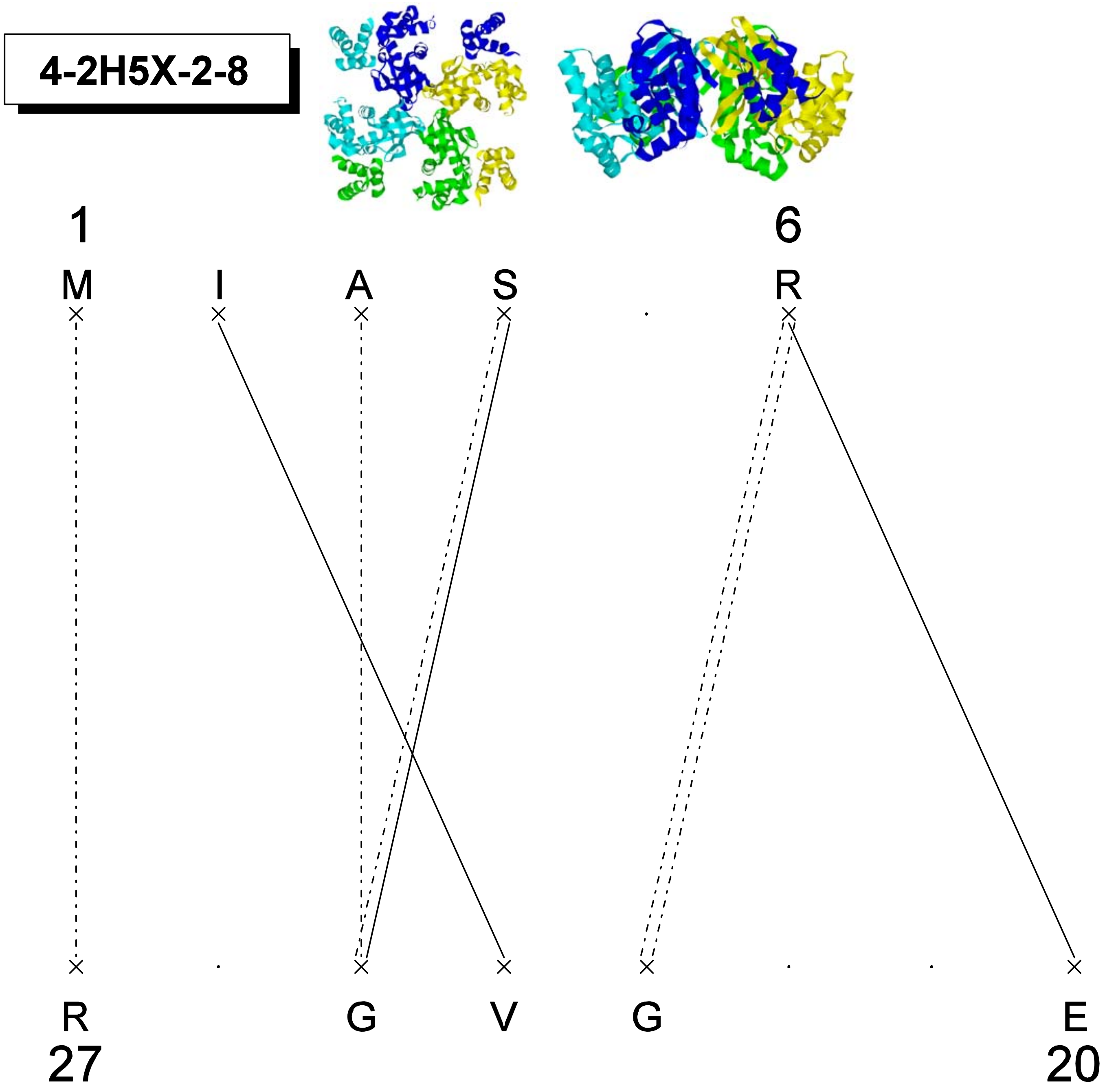
4-2H5X-2-8
1
6
M
I
A
S
R
R
G
V
G
E
27
20

4-3BF0-3-20

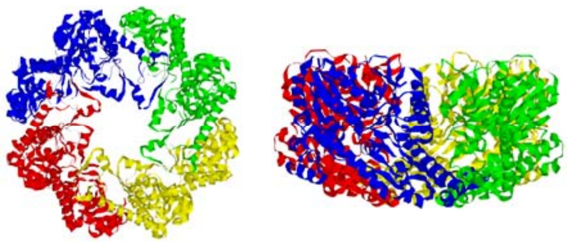

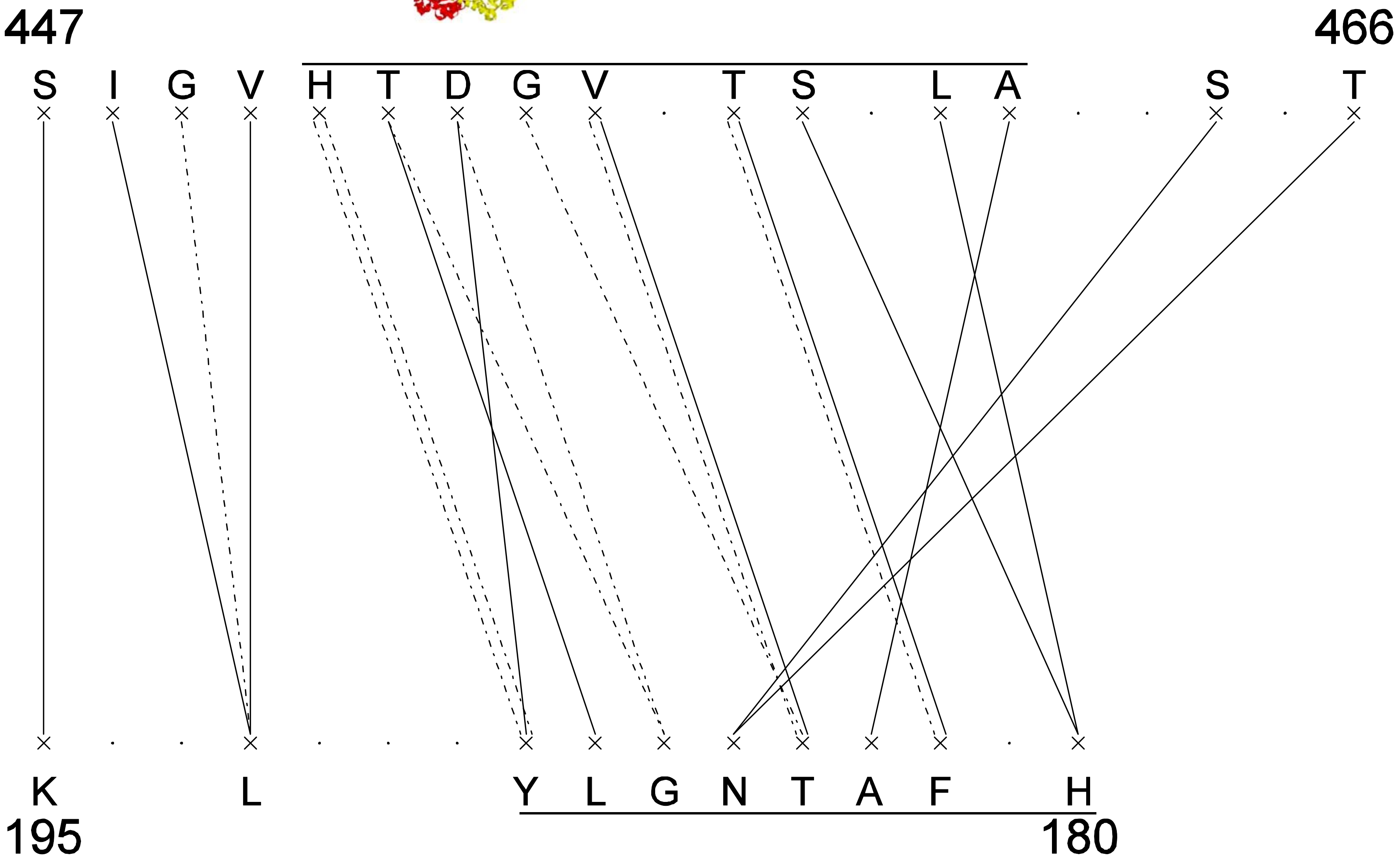

5-1b09-5-4

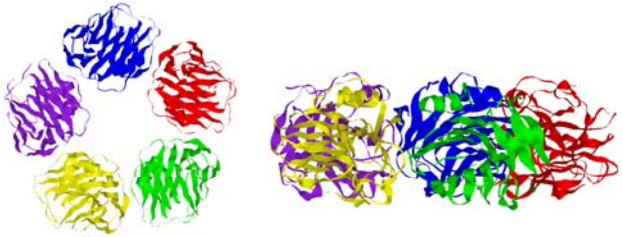

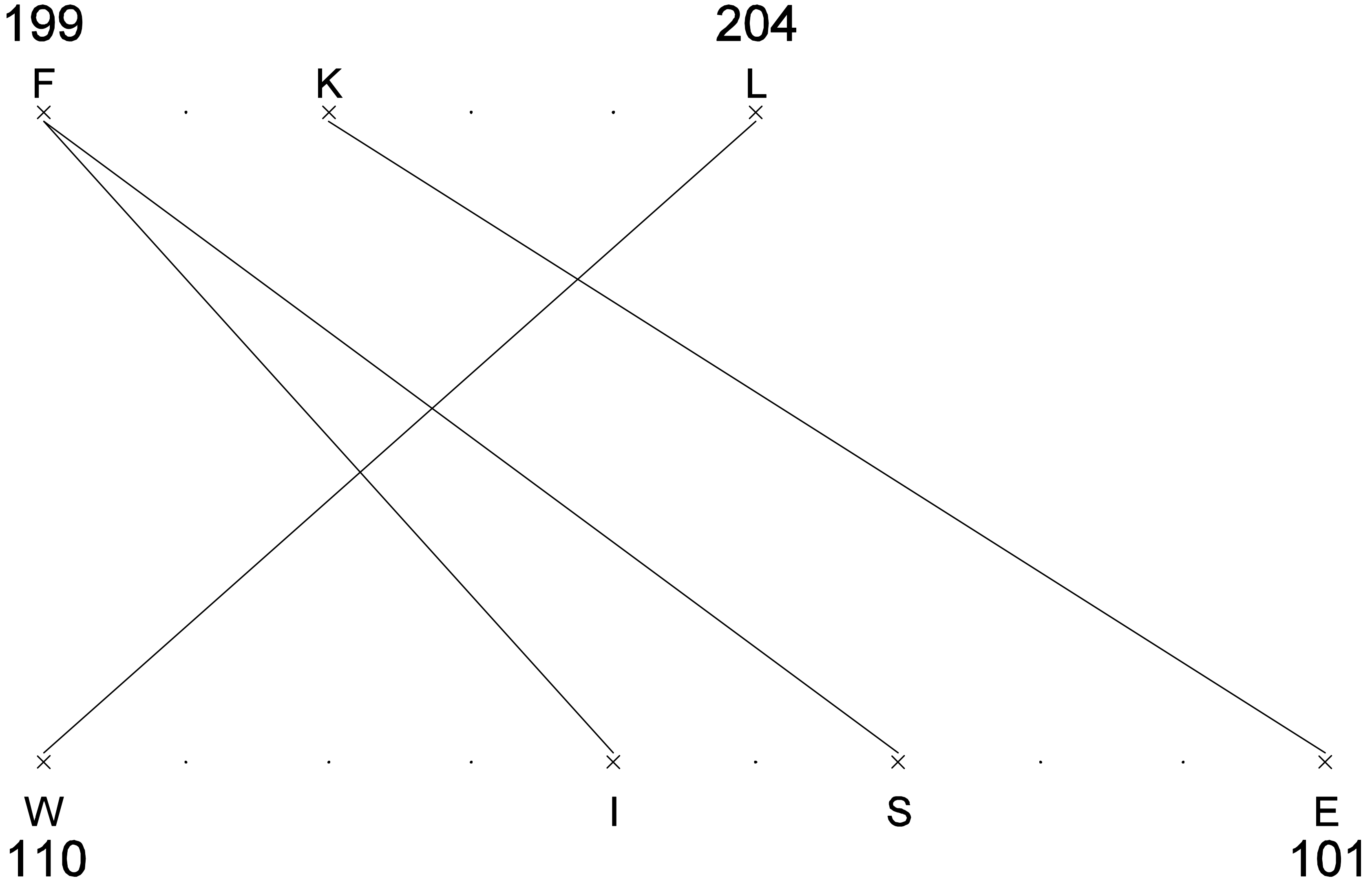

5-2XSC-3-7

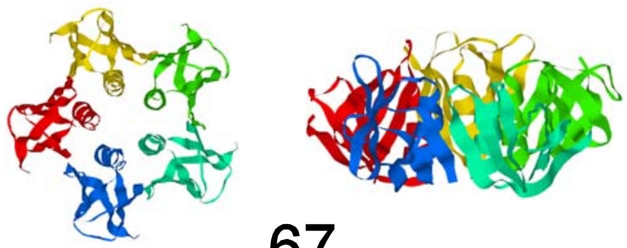

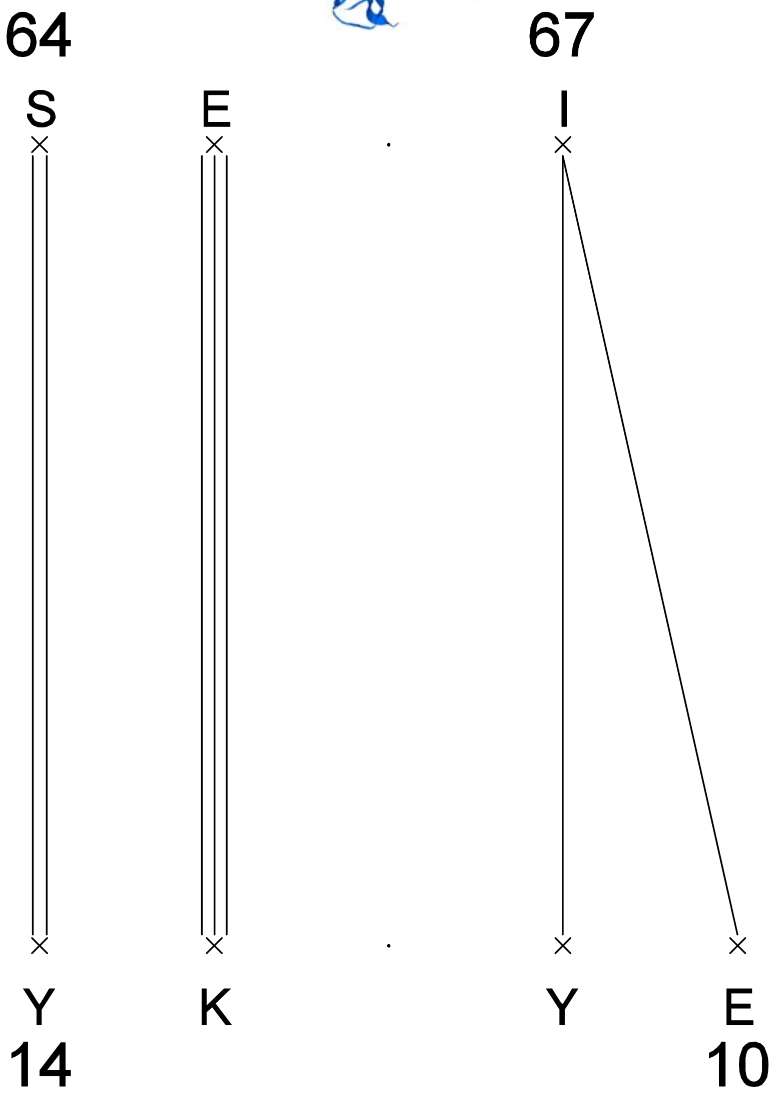

5-1EEI-6-10

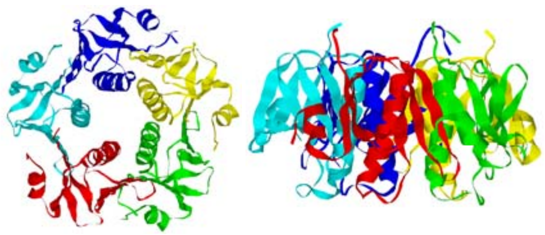

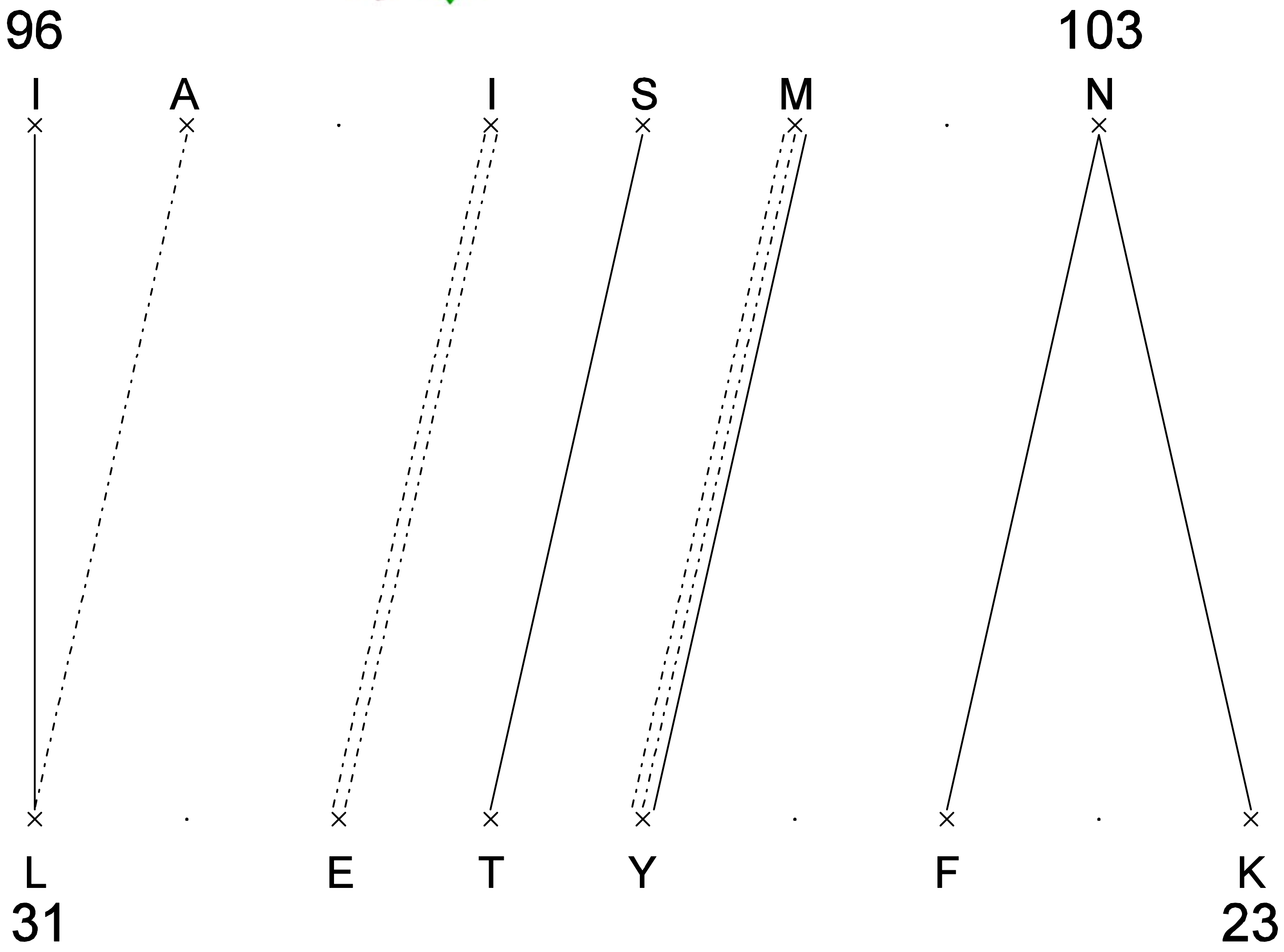

5-1EFI-1-10

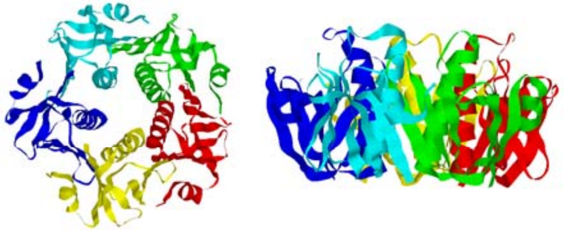

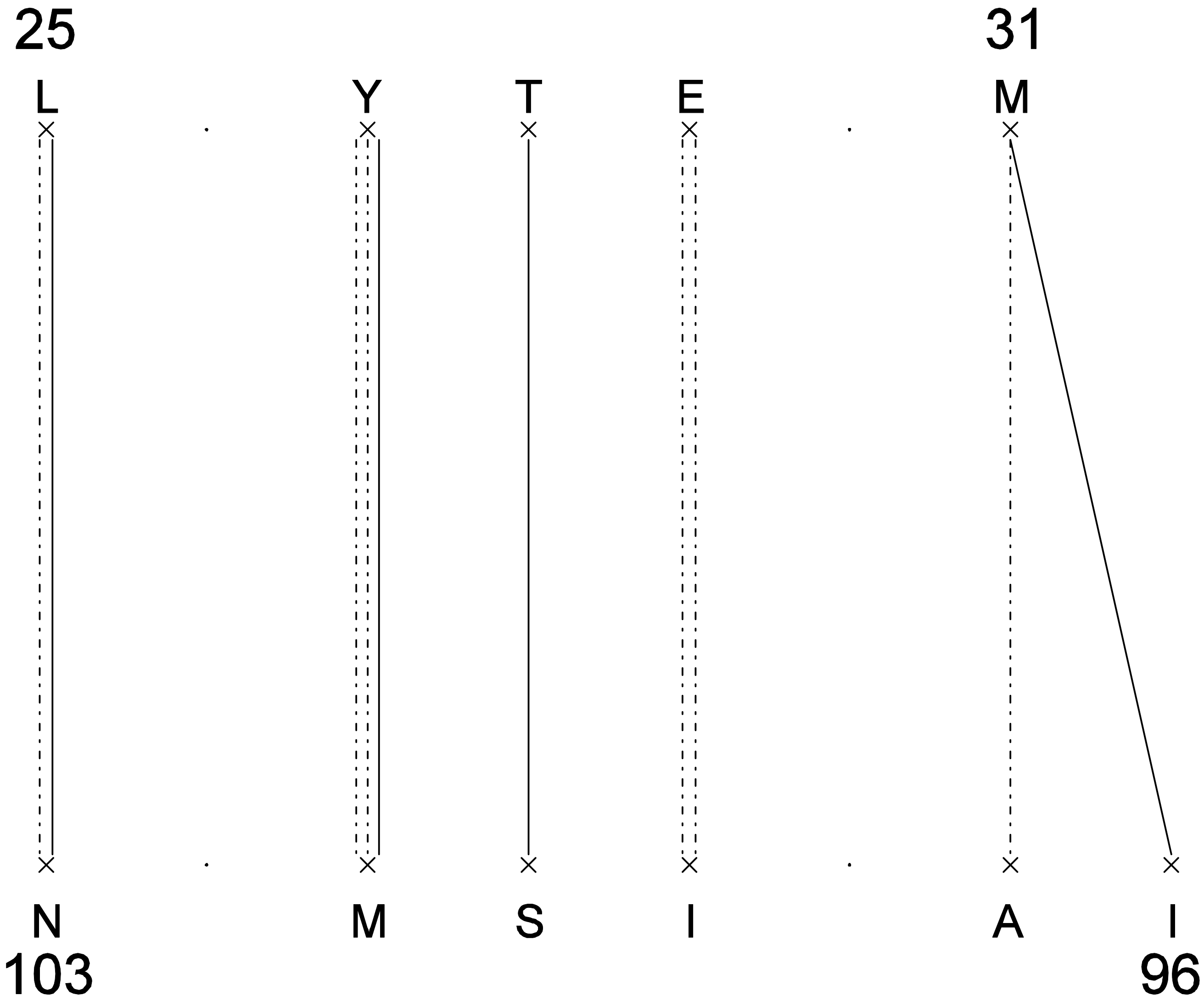

5-1fb1-1-13

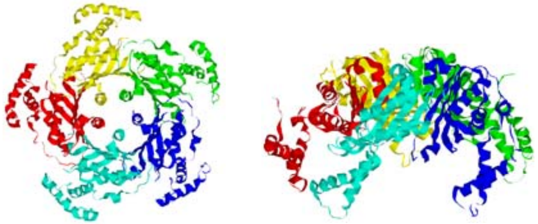

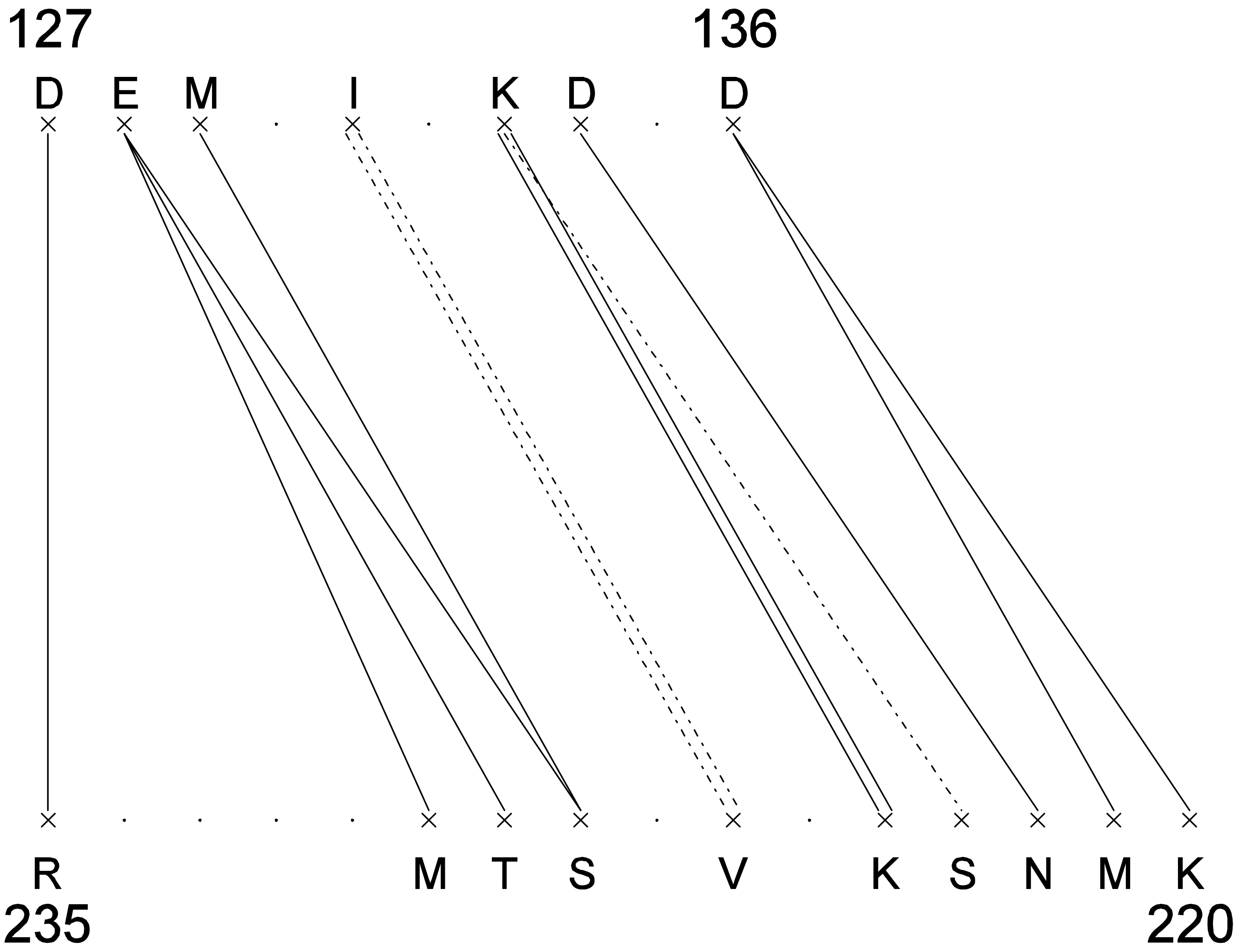

5-1hi9-1-15

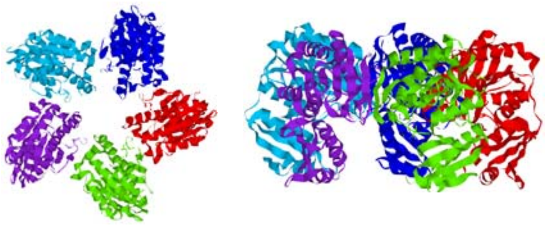

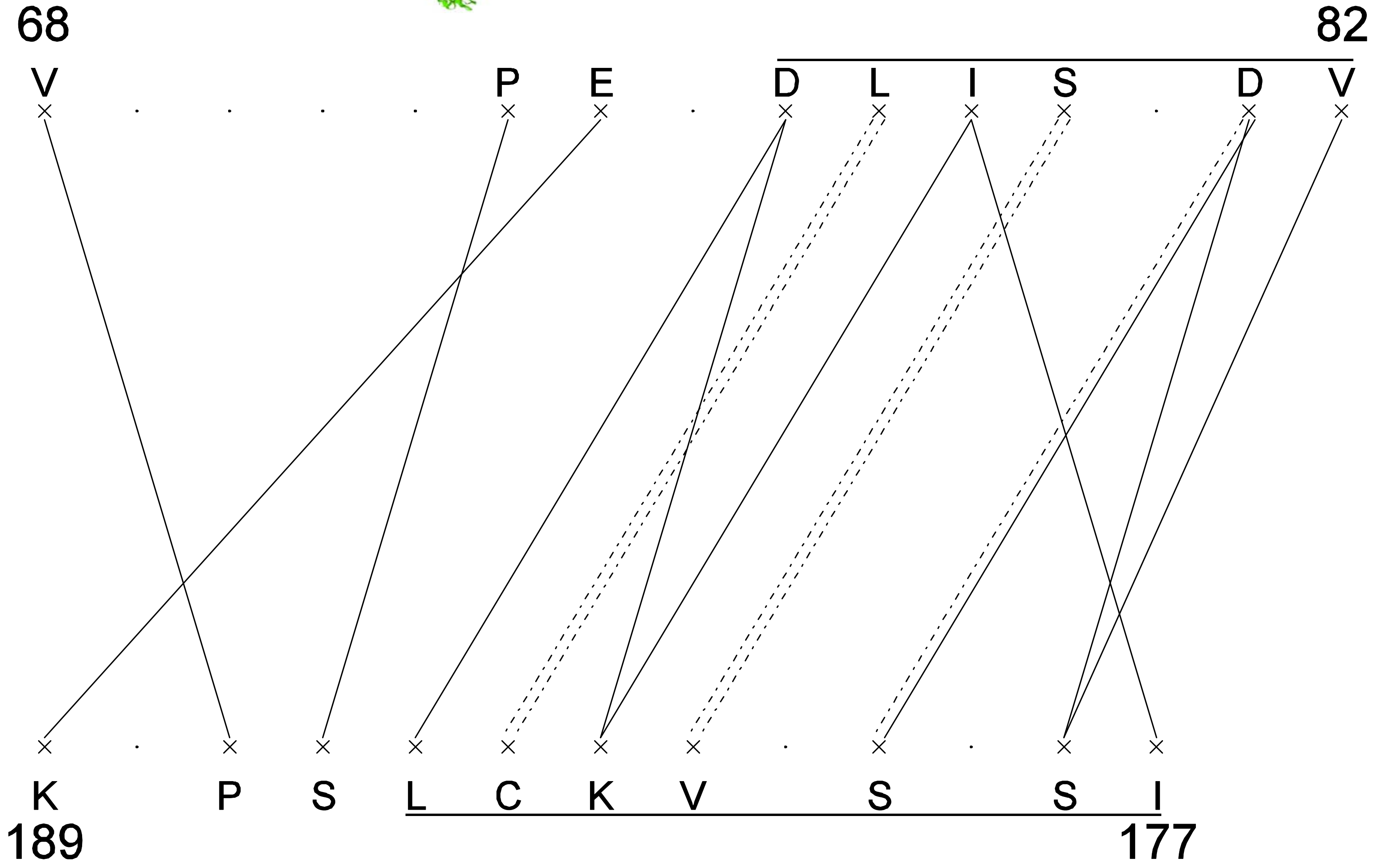

5-1nqu-1-9

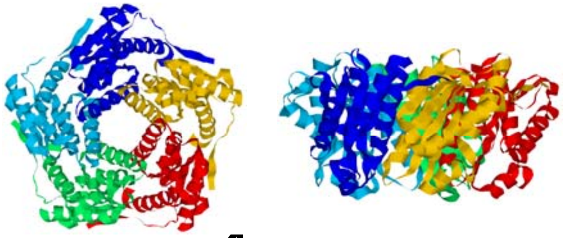

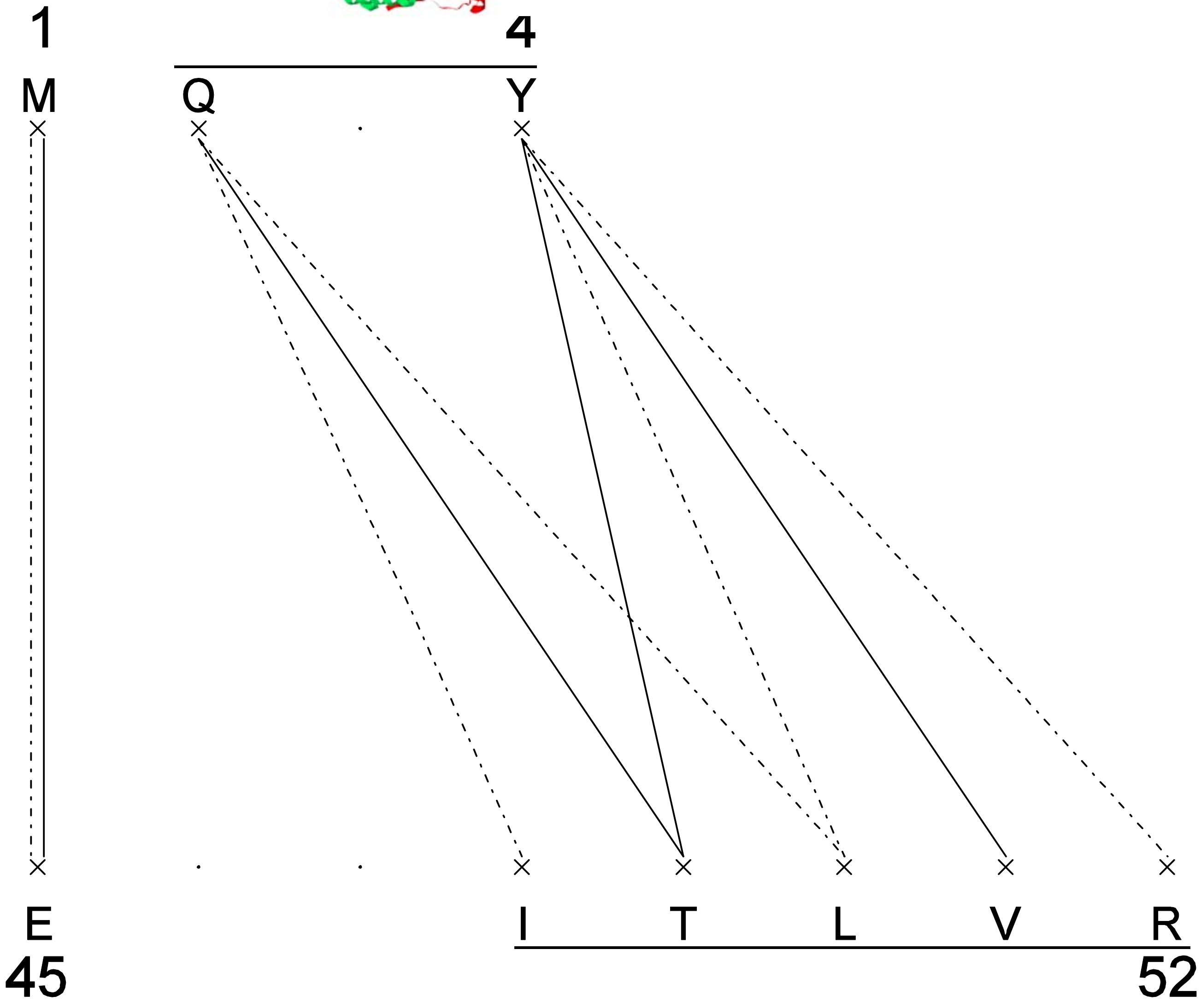

5-1sac-3-2

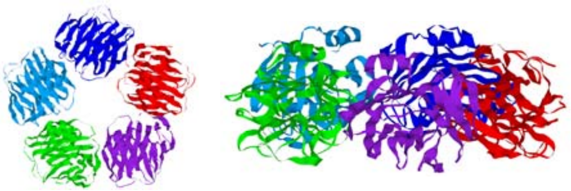

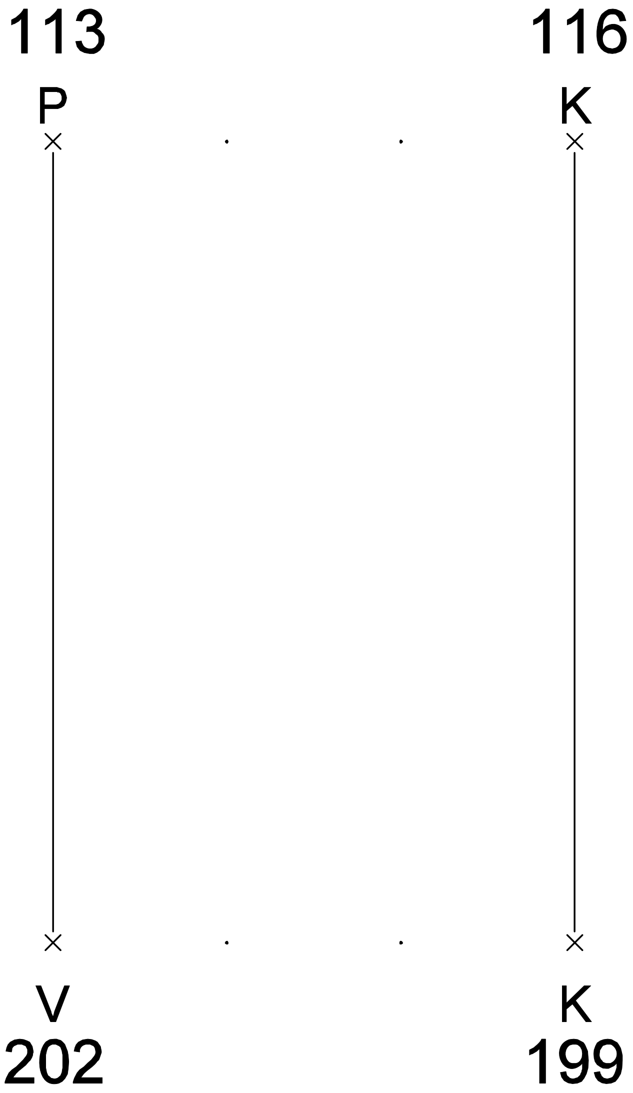

5-1wur-3-11

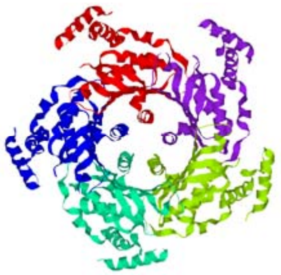

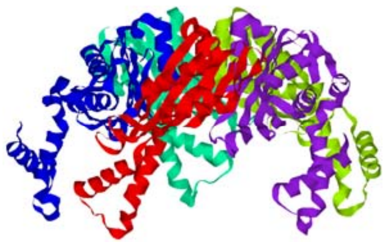

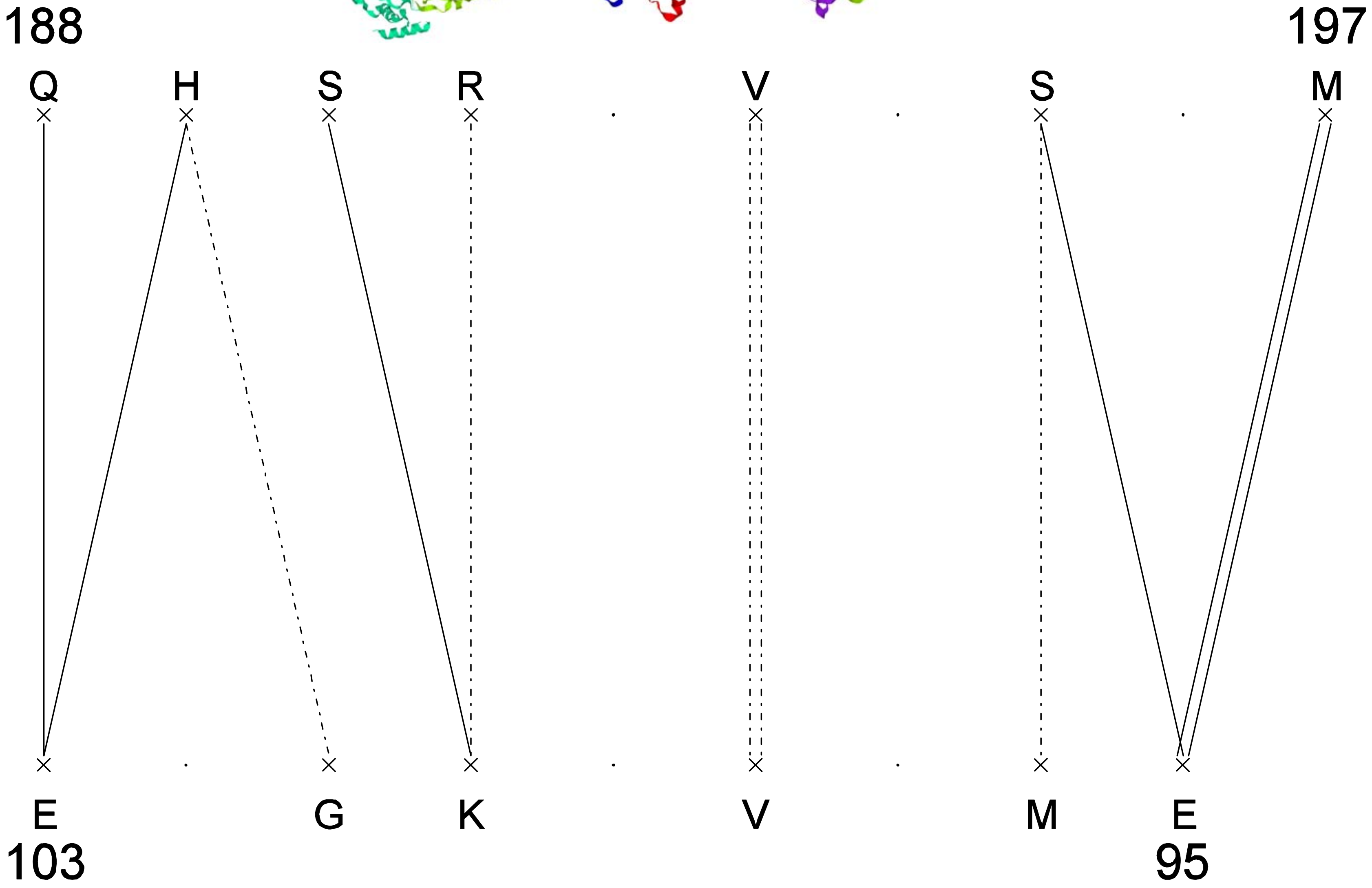

5-2ojw-6-5

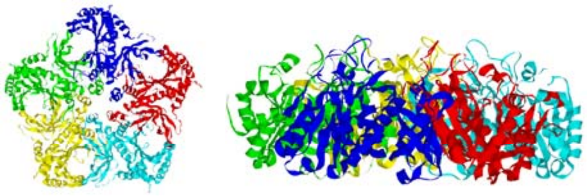

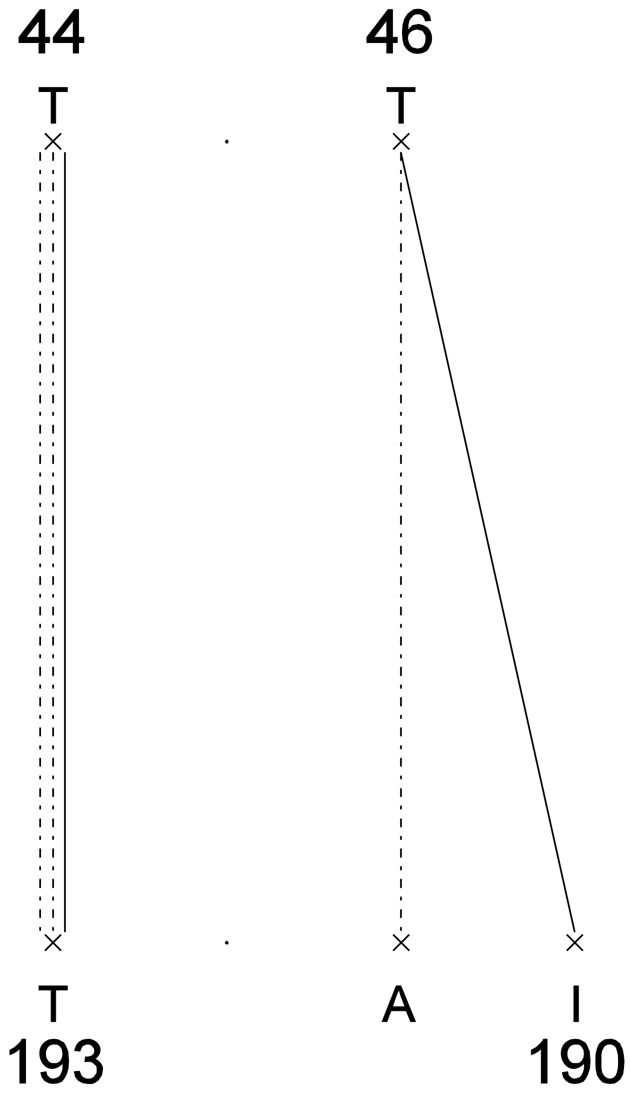

5-2rcf-4-9

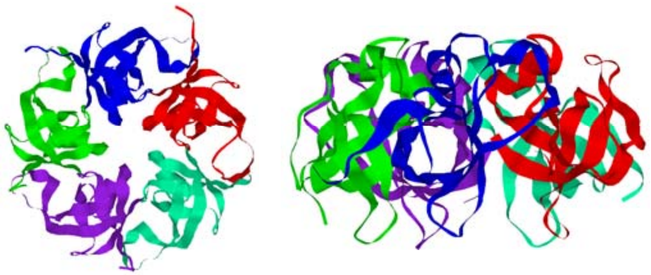

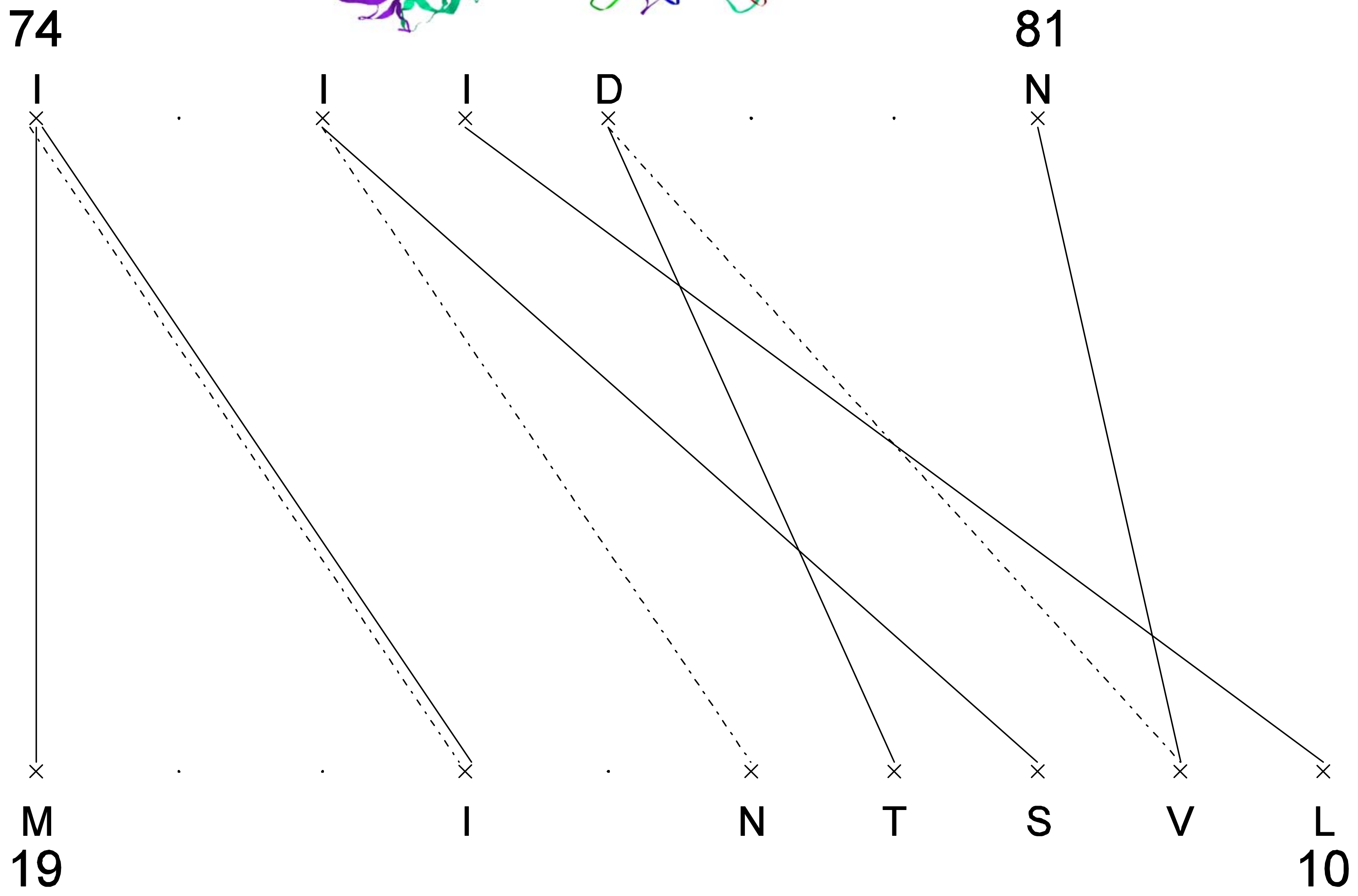

6-1U1S-1-12

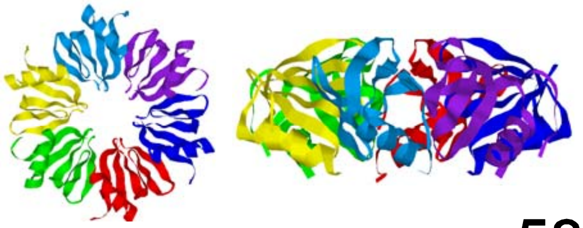

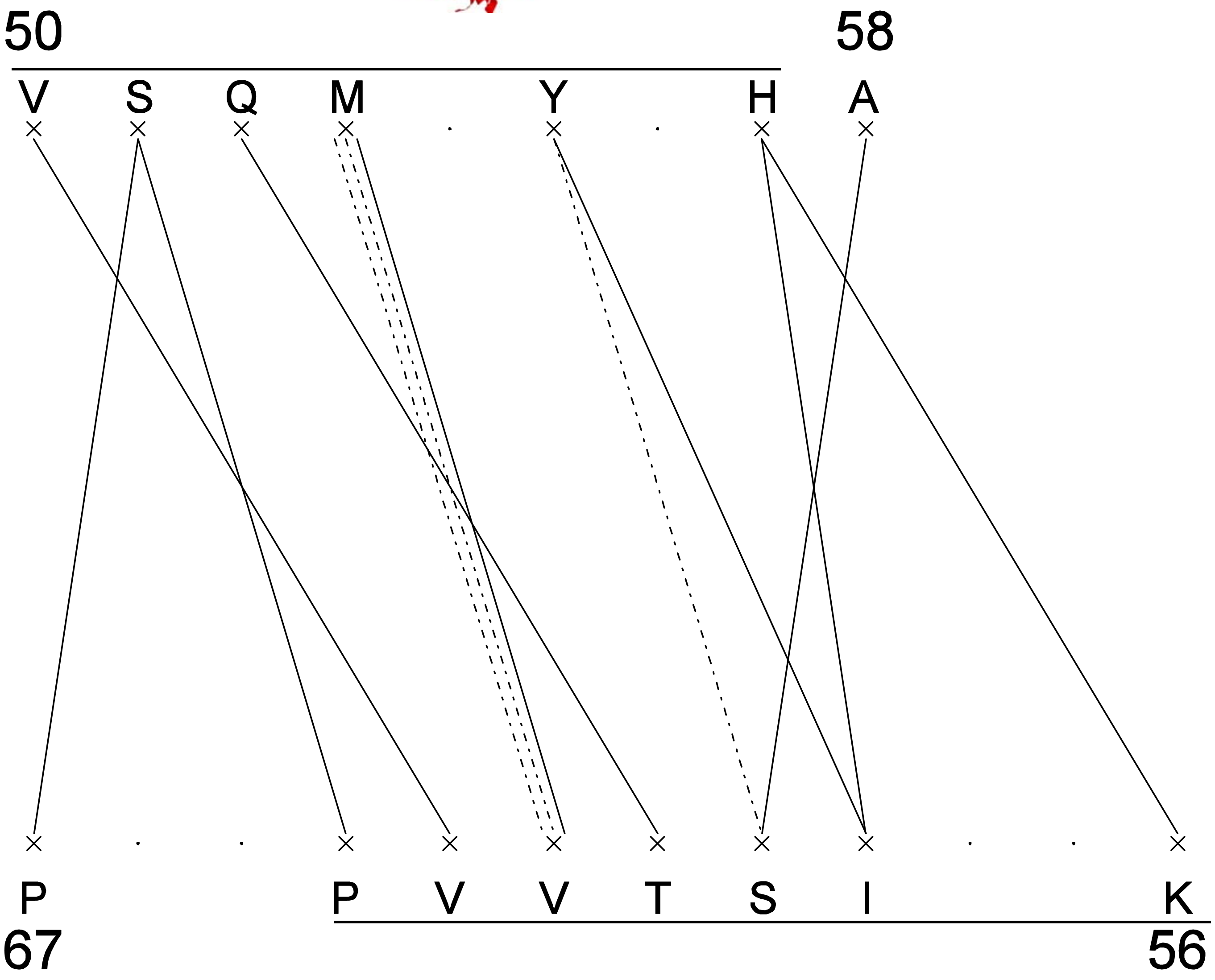

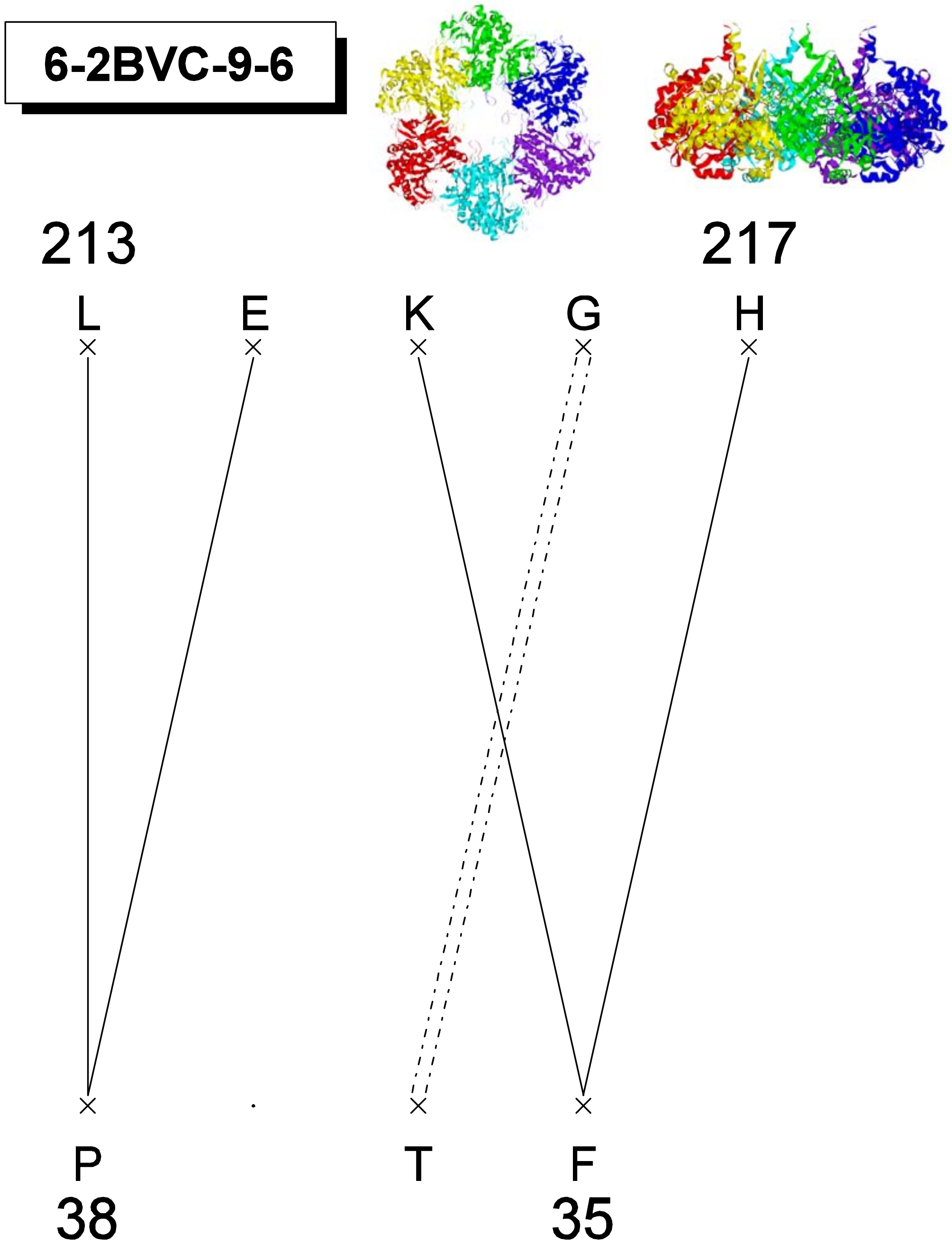

6-2BVC-9-6
213
217
L
E
K
G
H
P
T
F
38
35

6-2GJV-4-10

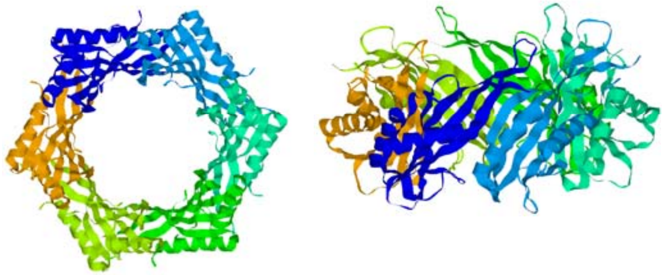

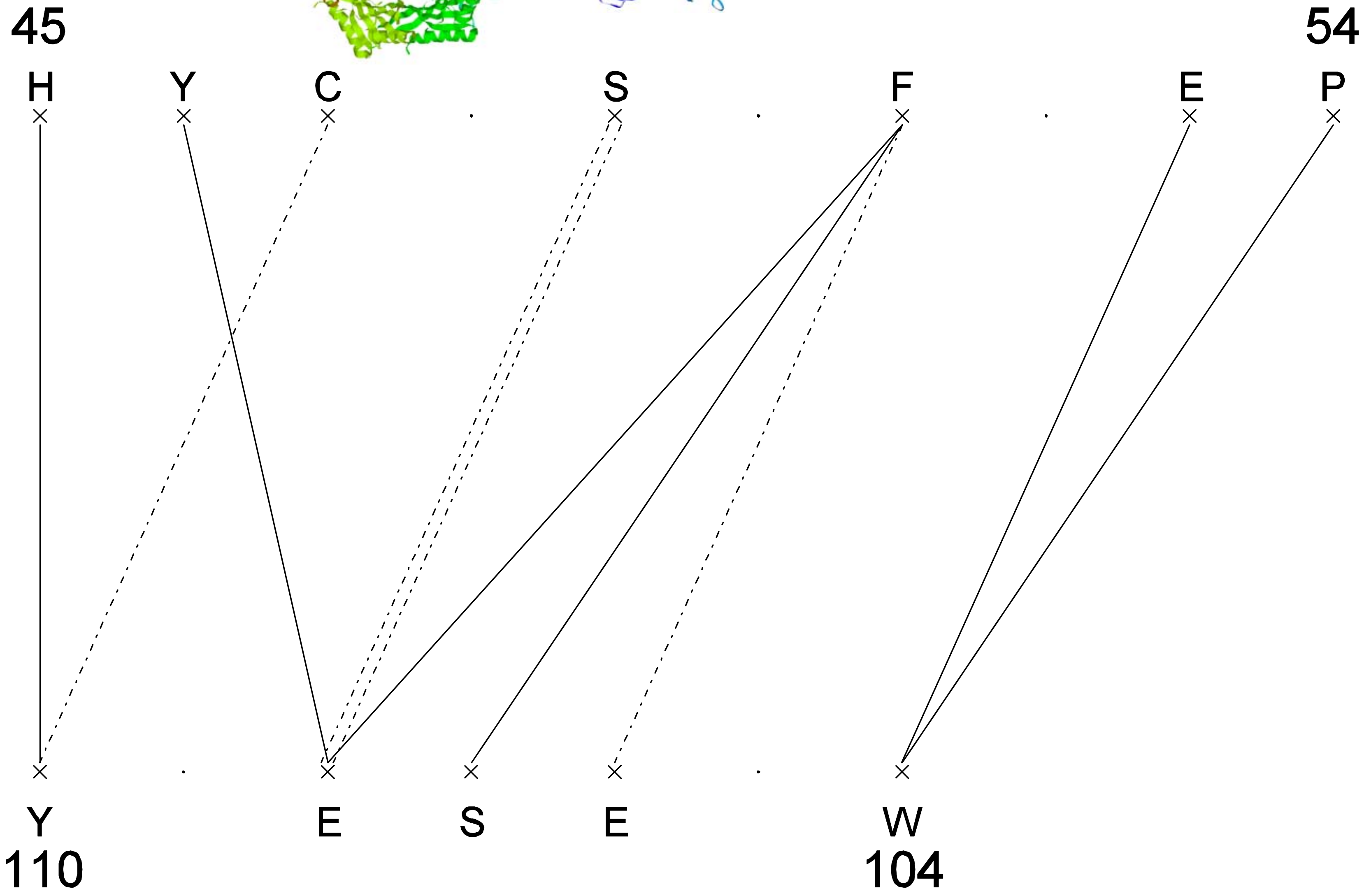

6-2Z9H-1-11

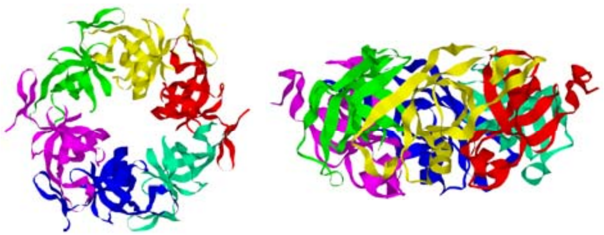

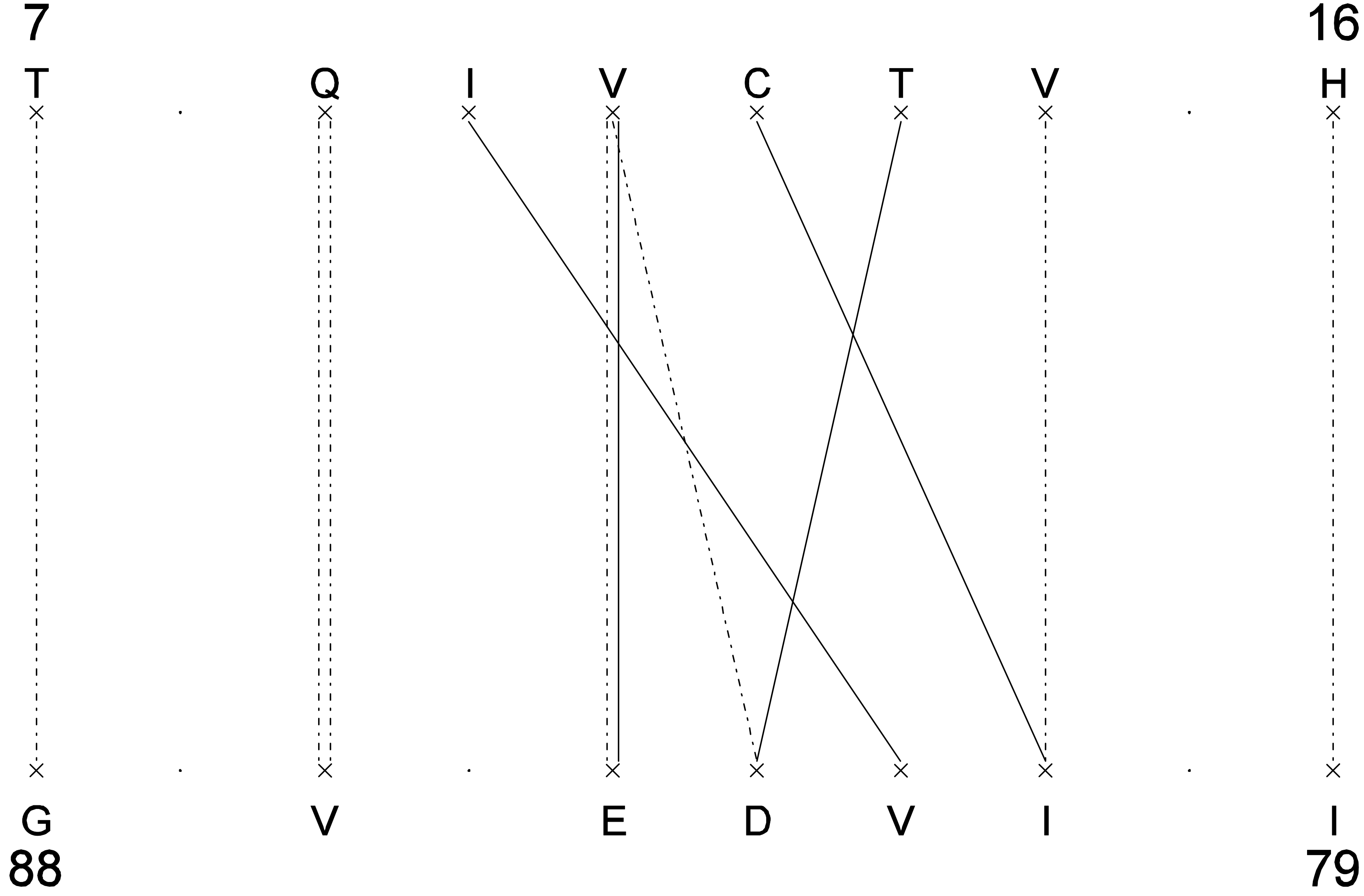

7-1HX5-1-7

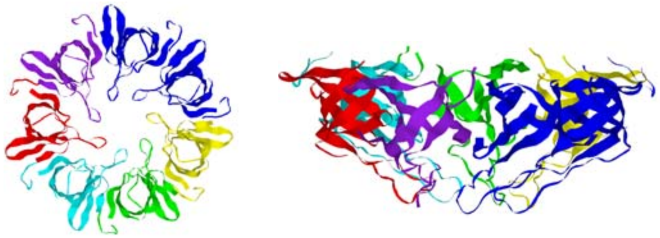

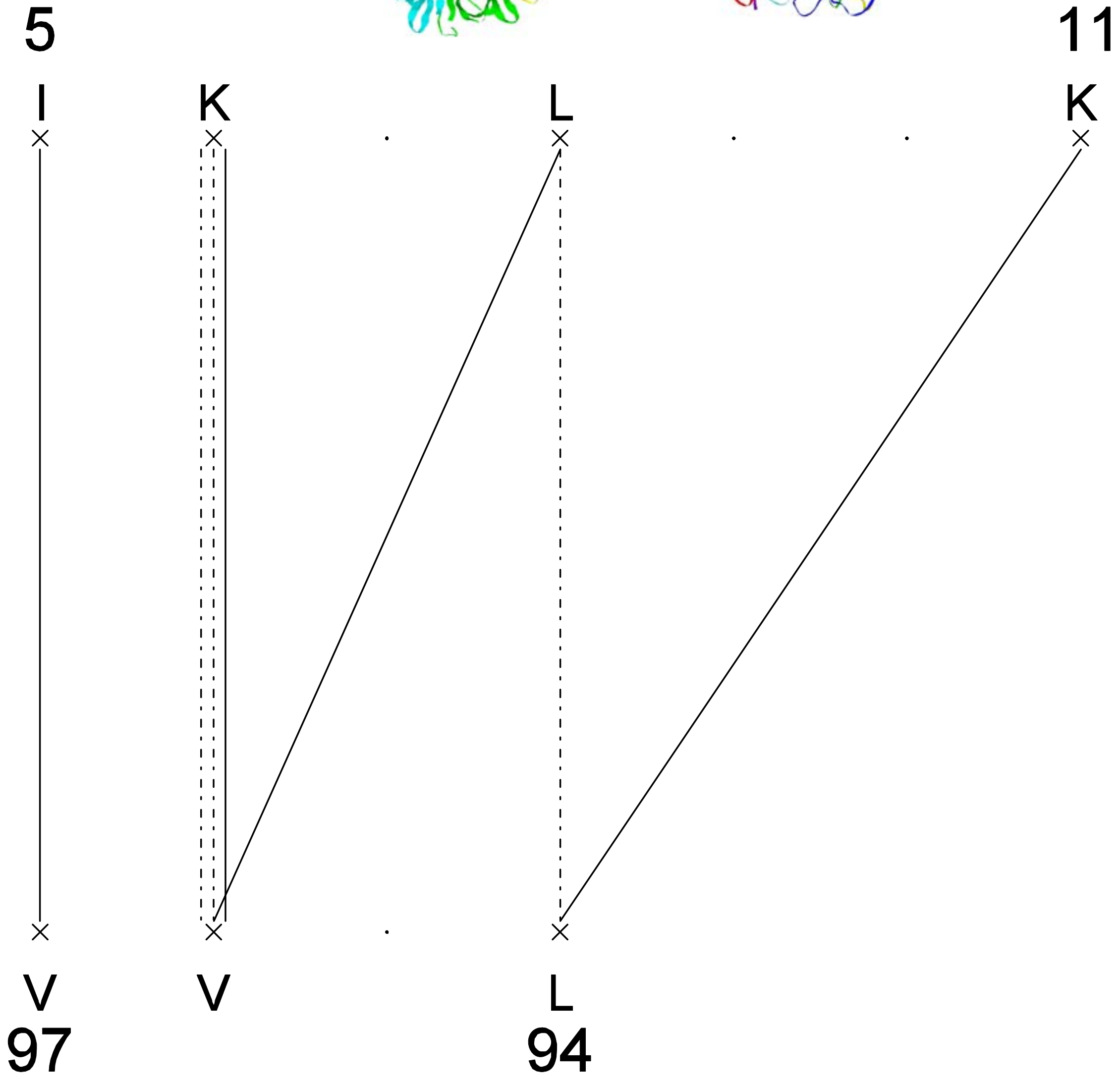

7-1OEL-8-12

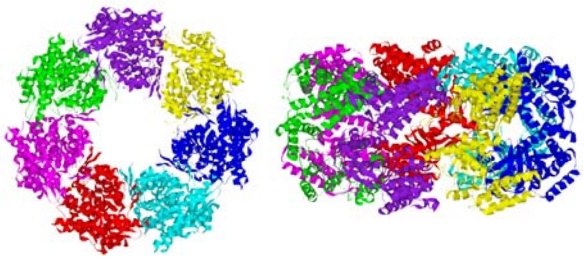

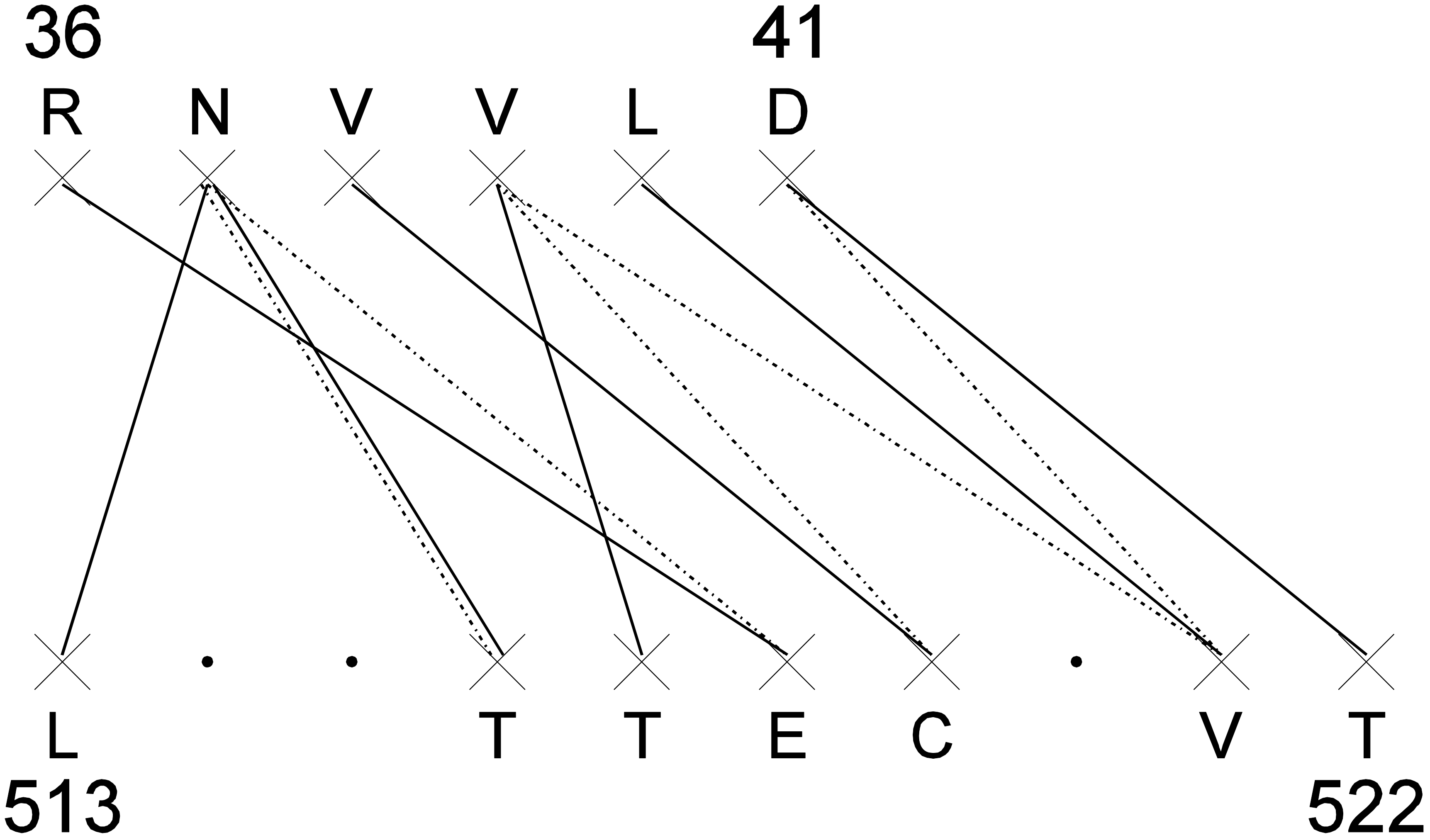

7-1WNR-1-8

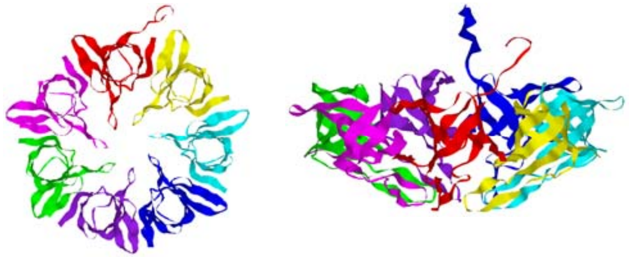

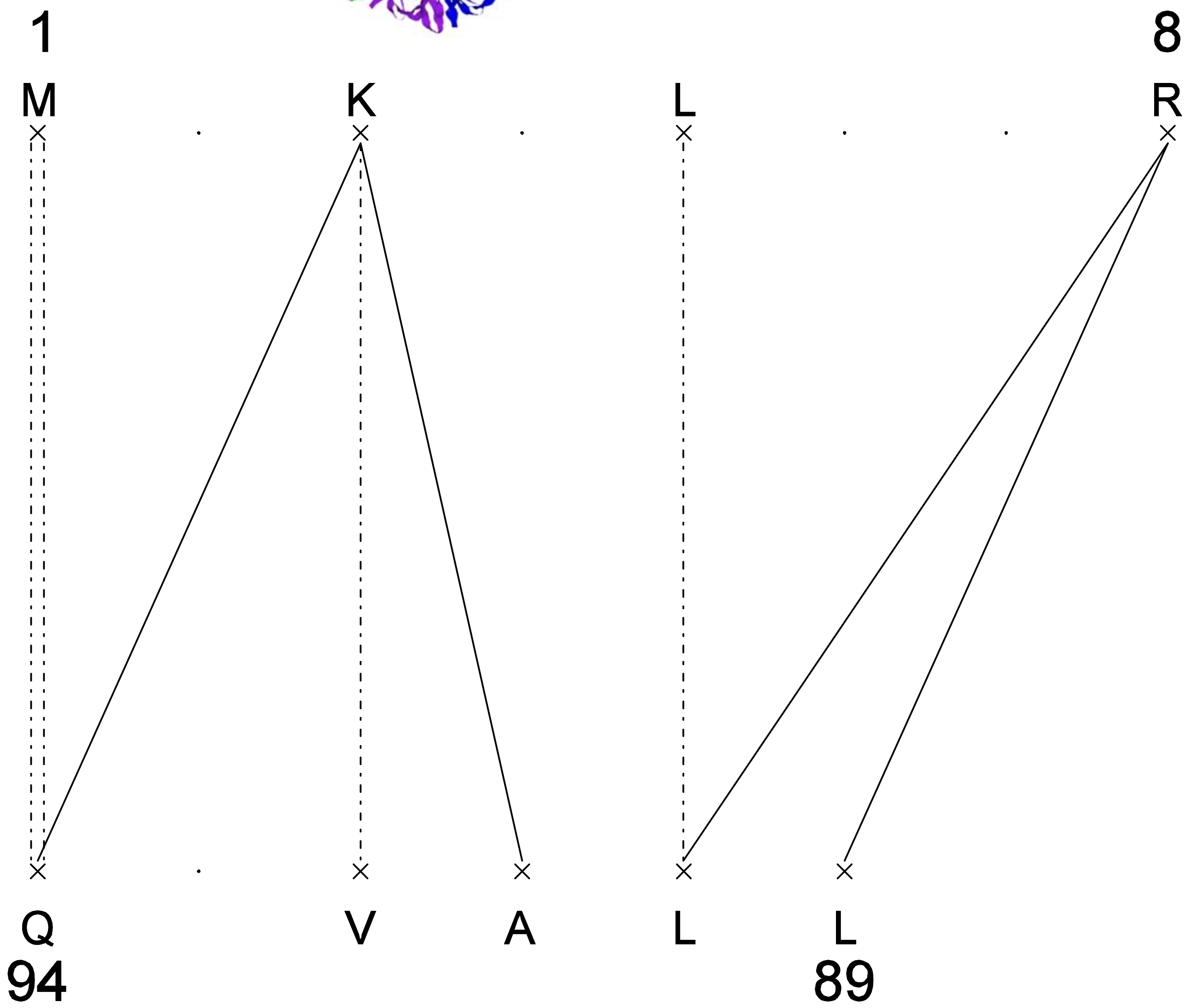

7-2RAQ-4-11

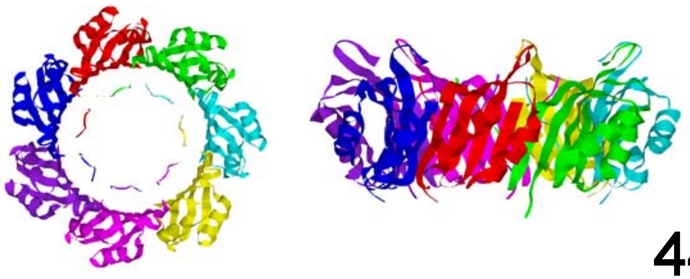

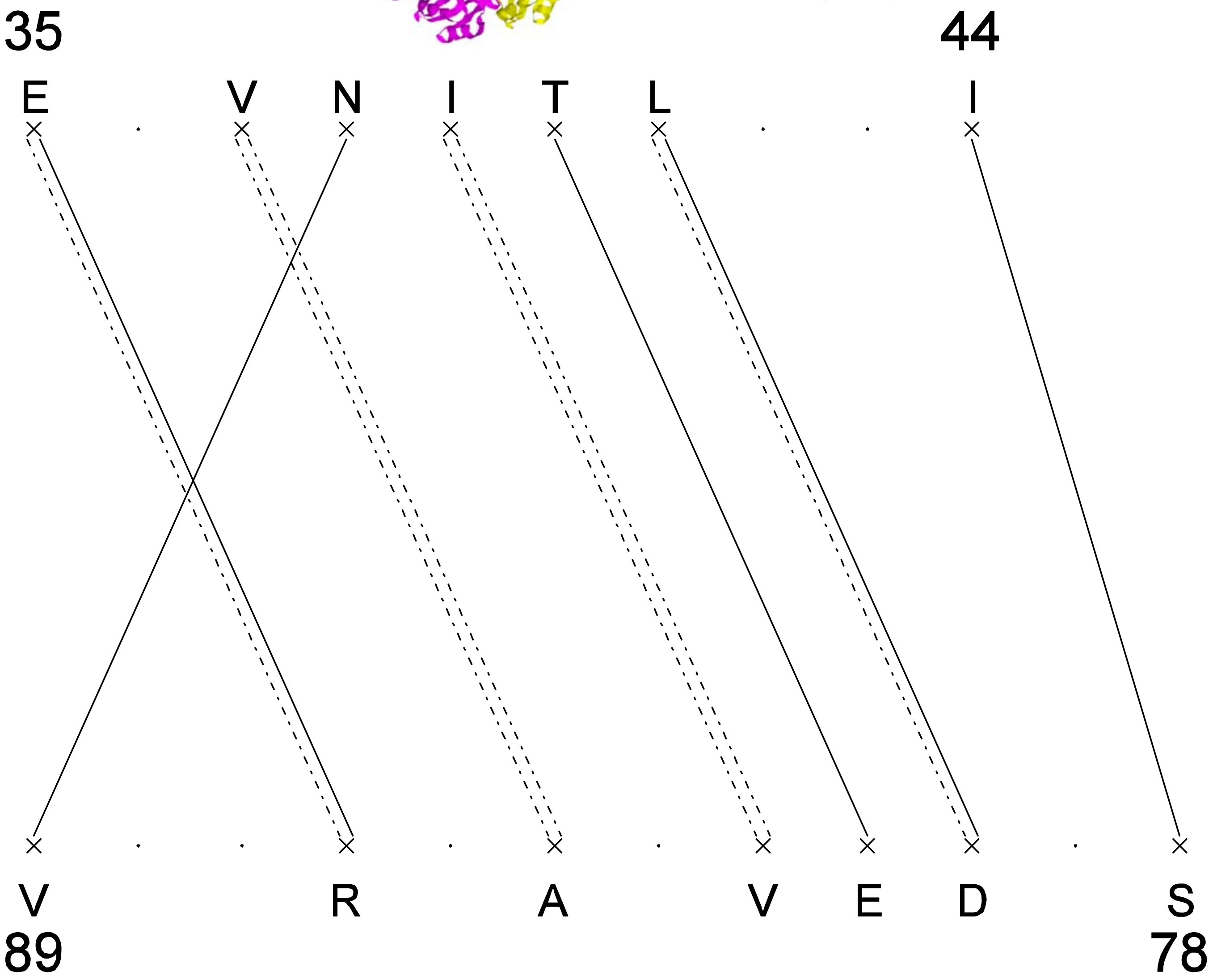

8-1Q3S-1-14

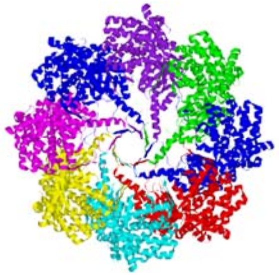

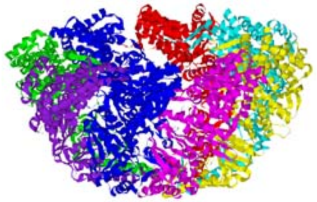

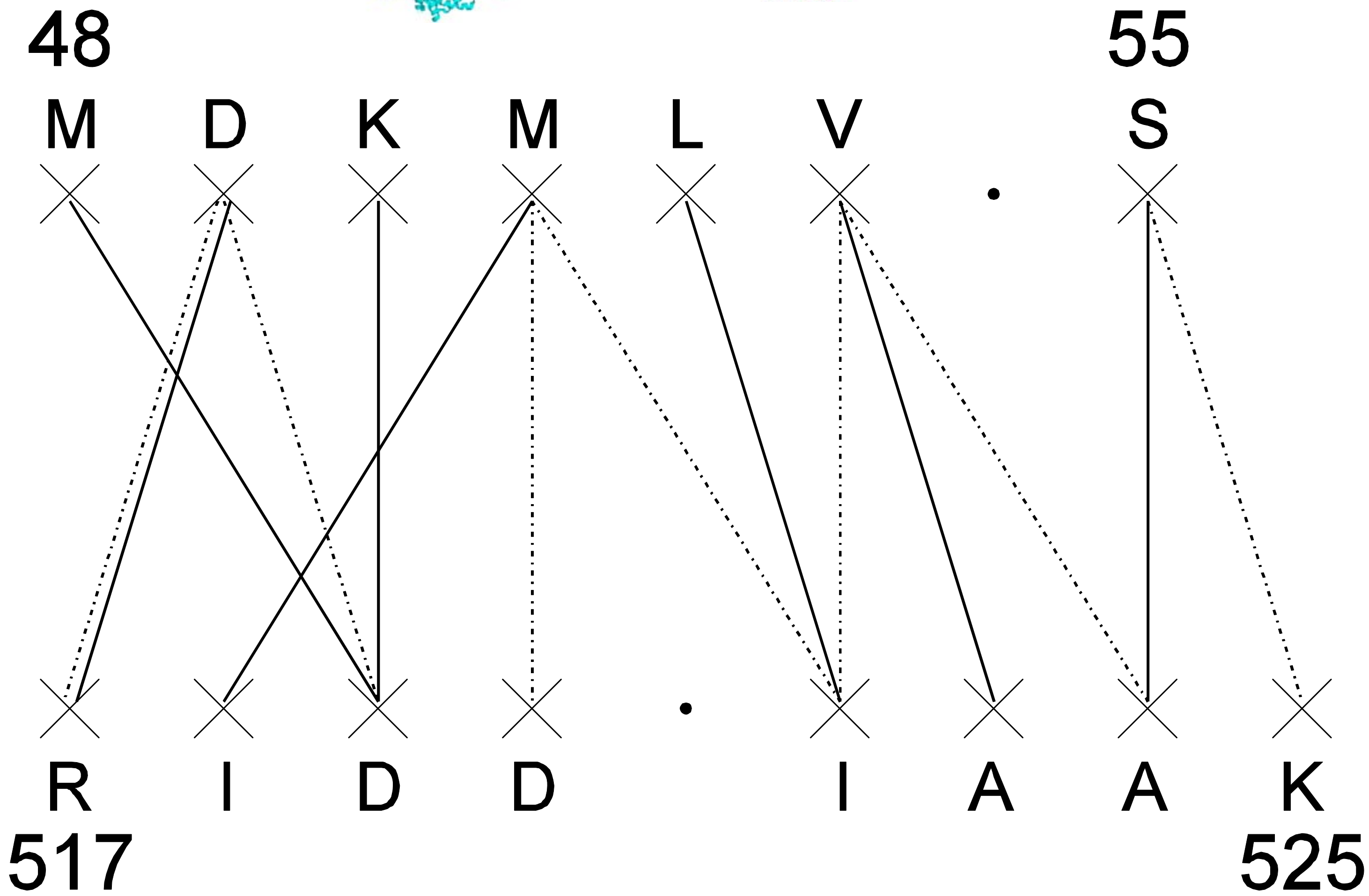

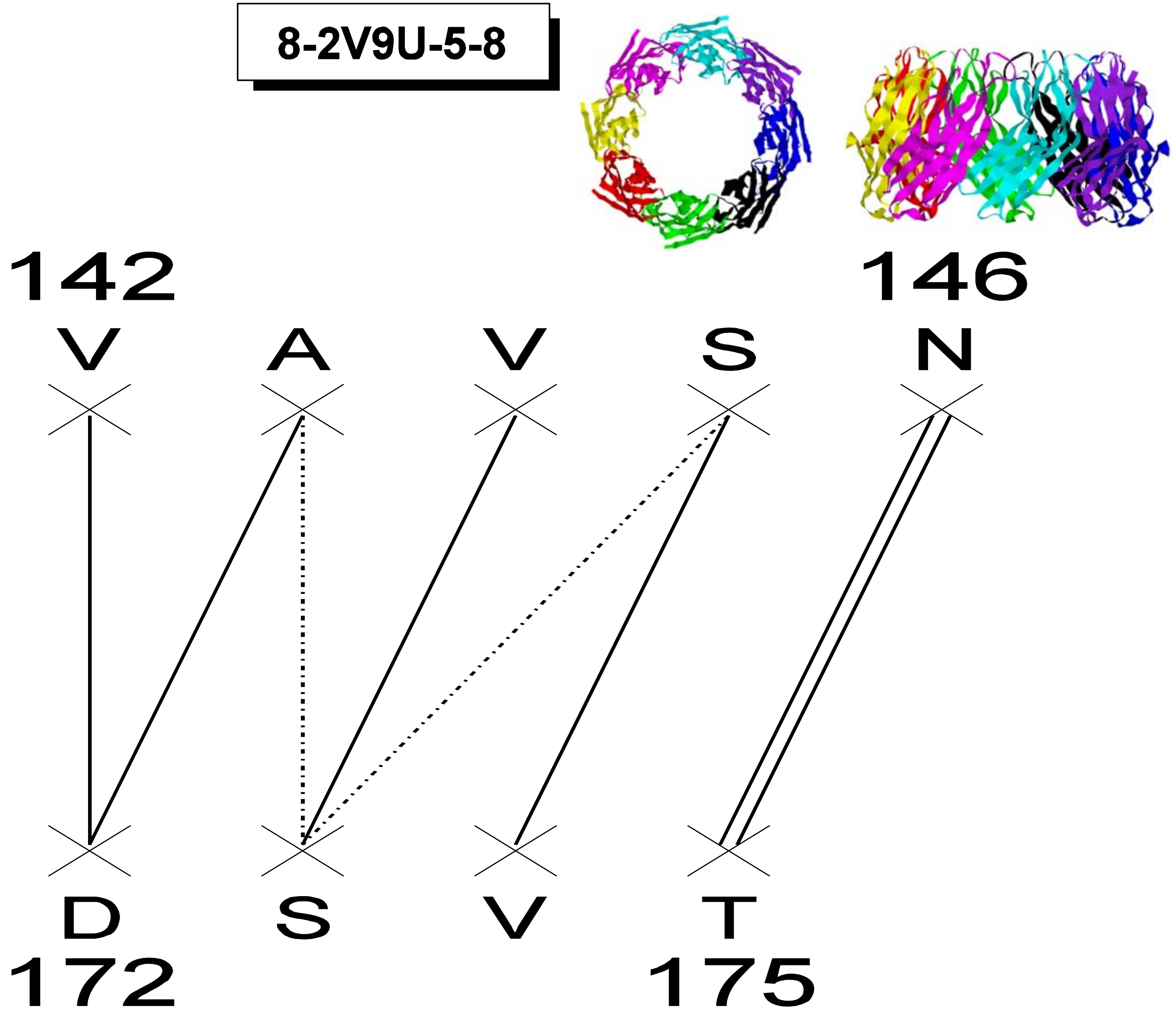
8-2V9U-5-8
142
146
V A V S N
D S V T
172
175
