# Supplementary material for: Beta-Strand Interfaces of Non-Dimeric Protein Oligomers Are Characterized by Scattered Charged Residue Patterns
Source: PLoS One. 2012 Apr 9;7(4):e32558. doi: 10.1371/journal.pone.0032558 (PMC3322119; doi:10.1371/journal.pone.0032558)
Supplement: Table S1 — Features of the protein oligomers of the dataset. (DOC) [file pone.0032558.s002.doc]

**Table S1.** Features of the protein oligomers of the dataset. ^a^ The superfamily has been obtained from SCOP (<http://scop.mrc-lmb.cam.ac.uk/scop/>). There is no SCOP entry when a question mark is indicated.

| **PDB** | **Stoichiometry** | **Organism** | **Function** | **Superfamily^a^** | **Whole chain** |  |
| --- | --- | --- | --- | --- | --- | --- |
| **1JN1** | 3 | Bacterium | Cyclodiphosphate synthase | 69765 | 157 |  |
| **1PM4** | 3 | Bacterium | Toxin | 101606 | 117 |  |
| **1SJN** | 3 | Bacterium | Hydrolase | 51283 | 131 |  |
| **1SNR** | 3 | Bacterium | Oxydoreductase | 49503 | 336 |  |
| **1T0A** | 3 | Bacterium | Cyclodiphosphate synthase | 69765 | 158 |  |
| **1Y13** | 3 | P. falciparum | Synthase | 55620 | 163 |  |
| **2BAZ** | 3 | Bacterium | Unknown | ? | 128 |  |
| **2BCM** | 3 | Bacterium | Adhesin | ? | 138 |  |
| **2BT9** | 3 | Bacterium | Lectin | ? | 90 |  |
| **2GVH** | 3 | Bacterium | Hydrolase | 54637 | 248 |  |
| **2I9D** | 3 | Bacteroid | Transferase | ? | 213 |  |
| **2JCA ^b^** | 3 | Bacterium | Transferase | ? | 123 |  |
| **2P90^c^** | 3 | Bacterium | Unknown | 159659 | 269 |  |
| **1J8D** | 4 | Bacterium | Hydrolase | 56784 | 179 |  |
| **1L3A** | 4 | PLANT | DNA-binding | 54447 | 166 |  |
| **1PVN** | 4 | Protozoa | Oxidoreductase | 51412 | 362 |  |
| **2A7R ^c^** | 4 | Homo sapiens | Oxidoreductase | ? | 338 |  |
| **2H5X** | 4 | Bacterium | DNA-binding | ? | 183 |  |
| **3BFO^c^** | 4 | Bacterium | Hydrolase | ? | 474 |  |
| **1B09** | 5 | Homo sapiens | Homo sapiens | 49899 | 206 |  |
| **2XSC** | 5 | Bacterium | Verotoxin | ? | 69 |  |
| **1EEI** | 5 | Bacterium | Toxin | 50203 | 103 |  |
| **1EFI** | 5 | Bacterium | Toxin | 50203 | 103 |  |
| **1FB1^c^** | 5 | Homo sapiens | Hydrolase | 51735 | 196 |  |
| **1HI9 ^c^** | 5 | Bacterium | Hydrolase | 63992 | 274 |  |
| **1NQU^c^** | 5 | Bacterium | Transferase | 52121 | 154 |  |
| **1SAC** | 5 | Homo sapiens | Amyloid | 49899 | 204 |  |
| **1WUR** | 5 | Bacterium | Hydrolase | 55620 | 185 |  |
| **2OJW** | 5 | Homo sapiens | Ligase | ? | 360 |  |
| **2RCF^c^** | 5 | Shell | Unknown | 159133 | 82 |  |
| **1U1S^c^** | 6 | Bacterium | RNA-binding | 50182 | 66 |  |
| **2BVC** | 6 | Bacterium | Synthetase | 54368 | 475 |  |
| **2GJV** | 6 | Bacterium | Unknown | 143749 | 136 |  |
| **2Z9H** | 6 | Bacterium | Structural | 159133 | 96 |  |
| **1HX5** | 7 | Bacterium | Cpn10 | 50129 | 82 |  |
| **1OEL** | 7 | Bacterium | Chaperone | 48592 | 524 |  |
| **1WNR** | 7 | Bacterium | Cpn10 | 50129 | 75 |  |
| **2RAQ** | 7 | Archea | Unknown | 160363 | 93 |  |
| **1Q3S** | 8 | Bacterium | Chaperone | 48592 | 517 |  |
| **2V9U** | 8 | Bacterium | Porin | ? | 132 |  |
