# Supplementary material for: Beta-Strand Interfaces of Non-Dimeric Protein Oligomers Are Characterized by Scattered Charged Residue Patterns
Source: PLoS One. 2012 Apr 9;7(4):e32558. doi: 10.1371/journal.pone.0032558 (PMC3322119; doi:10.1371/journal.pone.0032558)
Supplement: Table S2 — Features of the β-interfaces. (DOC) [file pone.0032558.s003.doc]

**Table S2.** Characteristics of the β-interfaces. ^a^The length is the sum of the amino acids of the two segments S1 and S2. ^b^The hot spots are the sum of the amino acids detected as ‘X’ by Gemini considering both S1 and S2. ^c^number of intermolecular interactions in the β-interface. ^d^total number of intermolecular interactions in the whole chain; ^e^Number of Interfacial regions in the whole chain; ^f^average number of intermolecular interactions in a chain (I_tot_/R). ^*^β-interfaces in which Gemini’s graphs have been manually annotated (see material and methods).

| **Name** | **Length^a^** | **Hot spots^b^** | **I_β_^c^** | **I_tot_ ^d^** | **R^e^** | **I_av_^f^** | **I_β_/I_av_** |
| --- | --- | --- | --- | --- | --- | --- | --- |
| **1JN1** | 26 | 12 | 9 | 34 | 7 | 4.9 | 1.9 |
| **1PM4** | 19 | 13 | 10 | 12 | 2 | 6.0 | 1.7 |
| **1SJN** | 17 | 14 | 12 | 47 | 11 | 4.3 | 2.8 |
| **1SNR** | 30 | 17 | 13 | 49 | 11 | 4.5 | 2.9 |
| **1T0A** | 25 | 15 | 12 | 27 | 5 | 5.4 | 2.2 |
| **1Y13** | 19 | 11 | 14 | 28 | 4 | 7.0 | 2.0 |
| **2BAZ** | 17 | 12 | 9 | 36 | 7 | 5.1 | 1.8 |
| **2BCM** | 13 | 11 | 11 | 23 | 3 | 7.7 | 1.4 |
| **2BT9** | 23 | 17 | 11 | 21 | 5 | 4.2 | 2.6 |
| **2GVH** | 21 | 18 | 16 | 34 | 5 | 6.8 | 2.4 |
| **2I9D** | 26 | 22 | 19 | 39 | 6 | 6.5 | 2.9 |
| **2JCA** | 26 | 18 | 16 | 19 | 2 | 9.5 | 1.7 |
| **2P90^c^** | 17 | 13 | 11 | 33 | 3 | 11.0 | 1.0 |
| **1J8D** | 14 | 13 | 10 | 1 | 4 | 6.8 | 1.5 |
| **1L3A** | 15 | 8 | 6 | 33 | 6 | 5.5 | 1.1 |
| **1PVN** | 7 | 6 | 5 | 41 | 8 | 5.1 | 1.0 |
| **2A7R ^*^** | 23 | 13 | 6 | 34 | 8 | 4.3 | 1.4 |
| **2H5X** | 11 | 10 | 8 | 35 | 9 | 3.9 | 2.1 |
| **3BFO^*^** | 19 | 17 | 14 | 36 | 6 | 6.0 | 2.3 |
| **1B09** | 16 | 7 | 4 | 14 | 5 | 2.8 | 1.4 |
| **2XSC** | 9 | 7 | 7 | 15 | 3 | 5.0 | 1.4 |
| **1EEI** | 17 | 12 | 10 | 31 | 6 | 5.2 | 1.9 |
| **1EFI** | 15 | 11 | 10 | 31 | 6 | 5.2 | 1.9 |
| **1FB1** | 26 | 17 | 13 | 31 | 5 | 6.2 | 2.1 |
| **1HI9 ^*^** | 17 | 13 | 12 | 19 | 2 | 9.5 | 1.3 |
| **1NQU^*^** | 10 | 7 | 7 | 36 | 6 | 6.0 | 1.2 |
| **1SAC** | 8 | 4 | 2 | 13 | 5 | 2.6 | 0.8 |
| **1WUR** | 19 | 13 | 11 | 31 | 4 | 7.8 | 1.4 |
| **2OJW** | 8 | 5 | 5 | 60 | 11 | 5.5 | 0.9 |
| **2RCF** | 14 | 12 | 9 | 21 | 4 | 5.3 | 1.7 |
| **1U1S^*^** | 12 | 10 | 10 | 21 | 3 | 7.0 | 1.3 |
| **2BVC** | 9 | 8 | 9 | 54 | 12 | 4.5 | 1.3 |
| **2GJV** | 17 | 12 | 10 | 24 | 6 | 4.0 | 2.5 |
| **2Z9H** | 20 | 15 | 11 | 20 | 3 | 6.7 | 1.7 |
| **1HX5** | 11 | 7 | 7 | 16 | 3 | 5.3 | 1.3 |
| **1OEL** | 16 | 13 | 11 | 36 | 8 | 4.5 | 2.4 |
| **1WNR** | 15 | 9 | 8 | 22 | 4 | 5.5 | 1.5 |
| **2RAQ** | 22 | 14 | 11 | 21 | 5 | 4.2 | 2.6 |
| **1Q3S** | 17 | 15 | 14 | 58 | 11 | 5.3 | 2.7 |
| **2V9U** | 9 | 9 | 8 | 21 | 7 | 3.0 | 2.7 |
| **Name** | **Length^a^** | **Hot spots^b^** | **I_β_^c^** | **I_tot_ ^d^** | **R^e^** | **I_av_^f^** | **I_β_/I_av_** |
