# Supplementary material for: Beta-Strand Interfaces of Non-Dimeric Protein Oligomers Are Characterized by Scattered Charged Residue Patterns
Source: PLoS One. 2012 Apr 9;7(4):e32558. doi: 10.1371/journal.pone.0032558 (PMC3322119; doi:10.1371/journal.pone.0032558)
Supplement: Table S3 — Properties of the two sub-graphs. (DOC) [file pone.0032558.s004.doc]

**Table S3.** Properties of the two sub-graphs

| **Name** | **I_β_** | **I_BB_** | **I_SC_** | **I_BB_/(I_BB_+I_SC_)** | **X_BB_** | **X_SC_** |
| --- | --- | --- | --- | --- | --- | --- |
| **1JN1** | 9 | 0 | 9 | 0 | 0 | 12 |
| **1PM4** | 10 | 3 | 7 | 0.3 | 6 | 9 |
| **1SJN** | 12 | 5 | 7 | 0.4 | 6 | 11 |
| **1SNR** | 13 | 7 | 6 | 0.5 | 10 | 10 |
| **1T0A** | 12 | 0 | 12 | 0.0 | 0 | 15 |
| **1Y13** | 14 | 8 | 6 | 0.6 | 9 | 9 |
| **2BAZ** | 9 | 5 | 4 | 0.6 | 6 | 8 |
| **2BCM** | 11 | 6 | 5 | 0.5 | 8 | 7 |
| **2BT9** | 11 | 1 | 9 | 0.1 | 2 | 15 |
| **2GVH** | 16 | 7 | 9 | 0.4 | 10 | 14 |
| **2I9D** | 19 | 6 | 13 | 0.3 | 9 | 18 |
| **2JCA ^b^** | 16 | 0 | 16 | 0.0 | 0 | 18 |
| **2P90^c^** | 11 | 5 | 6 | 0.5 | 6 | 8 |
| **1J8D** | 10 | 5 | 5 | 0.5 | 6 | 9 |
| **1L3A** | 6 | 4 | 2 | 0.7 | 5 | 4 |
| **1PVN** | 5 | 2 | 3 | 0.4 | 4 | 5 |
| **2A7R ^c^** | 6 | 3 | 3 | 0.5 | 5 | 6 |
| **2H5X** | 8 | 4 | 3 | 0.6 | 7 | 6 |
| **3BFO^c^** | 14 | 7 | 7 | 0.5 | 10 | 13 |
| **1B09** | 4 | 0 | 4 | 0.0 | 0 | 7 |
| **2XSC** | 7 | 0 | 7 | 0.0 | 0 | 7 |
| **1EEI** | 10 | 5 | 5 | 0.5 | 6 | 9 |
| **1EFI** | 10 | 6 | 4 | 0.6 | 8 | 8 |
| **1FB1^c^** | 13 | 3 | 10 | 0.2 | 4 | 14 |
| **1HI9 ^c^** | 12 | 5 | 6 | 0.5 | 6 | 9 |
| **1NQU^c^** | 7 | 5 | 2 | 0.7 | 7 | 6 |
| **1SAC** | 2 | 0 | 2 | 0.0 | 0 | 4 |
| **1WUR** | 11 | 5 | 5 | 0.5 | 8 | 8 |
| **2OJW** | 5 | 3 | 4 | 0.4 | 4 | 4 |
| **2RCF^c^** | 9 | 3 | 6 | 0.3 | 6 | 11 |
| **1U1S^c^** | 10 | 3 | 4 | 0.4 | 4 | 9 |
| **2BVC** | 9 | 2 | 4 | 0.6 | 2 | 6 |
| **2GJV** | 10 | 4 | 7 | 0.4 | 6 | 9 |
| **2Z9H** | 11 | 7 | 4 | 0.4 | 11 | 8 |
| **1HX5** | 7 | 3 | 5 | 0.5 | 4 | 7 |
| **1OEL** | 11 | 5 | 7 | 0.5 | 7 | 13 |
| **1WNR** | 8 | 4 | 4 | 0.5 | 6 | 6 |
| **2RAQ** | 11 | 6 | 5 | 0.3 | 8 | 10 |
| **1Q3S** | 14 | 7 | 7 | 0.4 | 10 | 13 |
| **2V9U** | 8 | 2 | 6 | 0.2 | 3 | 9 |
| **Name** | **I_β_** | **I_BB_** | **I_SC_** | **I_BB_/(I_BB_+I_SC_)** | **X_BB_** | **X_SC_** |
